# The impact of pulsatile vs. non-pulsatile perfusion in patients undergoing cardiopulmonary bypass: A comprehensive systematic review and meta-analysis of 33 randomized controlled trials

**DOI:** 10.1371/journal.pone.0333495

**Published:** 2025-10-14

**Authors:** Mohamed R. Abdelraouf, Abdelrahman Mahmoud, Ahmed Mazen Amin, Hazem Mohamed Salamah, Hassan Alshaker, Hazem Rezq, Ali Ashraf Salah Ahmed, Mohamed Ahmed Ali, Abdallah Saeed, Majd M. AlBarakat, Dina Ayman, Salem Elshenawy, Abdul Rhman Hassan, Basant E. Katamesh, Basel Abdelazeem

**Affiliations:** 1 Faculty of Medicine, Alexandria University, Alexandria, Egypt; 2 Faculty of Medicine, Minia University, Minia, Egypt; 3 Faculty of Medicine, Mansoura University, Mansoura, Egypt; 4 Faculty of Medicine, Zagazig University, Zagazig, Egypt; 5 Department of Internal Medicine, Allegheny Health Network, Pittsburgh, Pennsylvania, United States of America; 6 Faculty of Medicine, Al-Azhar University, Cairo, Egypt; 7 Qena Faculty of Medicine, South Valley University, Qena, Egypt; 8 Medical Research Group of Egypt, Negida Academy, Arlington, Massachusetts, United States of America; 9 Faculty of Medicine, Tanta University, Tanta, Egypt; 10 Faculty of Medicine, Jordan University of Science and Technology, Irbid, Jordan; 11 Faculty of Medicine, Beni suef university, Beni suef, Egypt; 12 General Internal Medicine, Mayo Clinic, Rochester, United States of America; 13 Cardiology Department, West Virginia University, Morgantown, West Virginia, United States of America; Children's Hospital of Los Angeles / Keck School of Medicine, UNITED STATES OF AMERICA

## Abstract

**Background:**

Pulsatile perfusion is a developing technique that attempts to mimic the natural pulsatile flow of blood during cardiopulmonary bypass (CBP).

**Purpose:**

This systematic review and meta-analysis was conducted to show the effects of pulsatile perfusion in CPB compared to non-pulsatile.

**Methods:**

Randomized control trials that evaluated the implementation of pulsatile perfusion during cardiopulmonary bypass surgery were identified by a literature search in the following electronic databases (PubMed, Web of Science, Scopus, CENTRAL, and Embase) published from inception up to February 2024.

**Results:**

The search yielded 33 trials of which three studies demonstrated a low risk of bias, 29 studies showed some concerns, and one study presented a high risk of bias overall. The total number of patients was 3174 patients. The analysis showed that pulsatile perfusion led to a significant decrease in creatinine level [MD = −0.14, 95% CI (−0.24, −.04), P < 0.004], lactate level [MD = −8.21, 95% CI (−13.16, −3.25), P < 0.001], hospital stay [MD = −1.38, 95% CI (−2.51, −0.25), P = 0.016], ICU stay [MD = −0.47, 95% CI (−0.82, −0.13), P = 0.007], intubation time [MD = −3.73, 95% CI (−5.42, −2.04), P < 0.001], and increase in creatinine clearance [MD = 10.08, 95% CI (3.36, 16.80), P < 0.003]. However, no significant difference between the two regimens was detected in estimated glomerular filtration rate (eGFR), alanine transferase (ALT) level, AST (aspartate transferase) level, Blood urea nitrogen (BUN) level, acute renal failure (ARF), and mortality rates.

**Conclusion:**

Pulsatile perfusion showed some positive effects on creatinine, creatinine clearance, lactate level, hospital stay, ICU stay, and intubation time. However, there was no difference between the two methods on BUN, ALT, AST, eGFR, ARF, and death. Most of the outcomes showed significant heterogeneity, which requires more robust RCTs to be conducted to increase the quality and the certainty of evidence.

## Introduction

Cardiopulmonary bypass (CPB) was invented in the mid-20th century, allowing for the performance of numerous heart surgeries, including those for coronary artery disease [1,2]. Despite recent advances in equipment and perfusion techniques, CPB has several drawbacks regarding blood perfusion to organs, particularly the brain and kidneys, due to induced vasoconstriction and blood redistribution away from these organs [1–3]. Research indicates that CPB triggers a systemic inflammatory response syndrome (SIRS) [4], leading to various postoperative complications such as myocardial dysfunction, respiratory failure, renal and neurological dysfunction, bleeding disorders, changes in liver function, and, potentially, multiple organ failure.

During CPB, two types of flows can be established: pulsatile flow, which mimics normal circulation, and non-pulsatile flow. A drawback of non-pulsatile flow is its reduced transmission of mechanical energy to the vascular wall, resulting in diminished endothelial shear stress. This decreased mechanical stimulation of arterial baroreceptors triggers a notable surge in sympathetic activity, causing further vasoconstriction and exacerbating peripheral blood flow impairment [5]. Additionally, the lower mechanical energy associated with non-pulsatile flow hampers the synthesis of shear-responsive endothelial-derived vasodilators like nitric oxide, contributing to progressive capillary collapse, microcirculatory shunting, and tissue hypoperfusion [5]. In theory, pulsatile flow is believed to circumvent the adverse effects of non-pulsatile flow on the endothelium.

Several studies have shown enhanced blood flow to the liver, kidneys, and stomach during cardiogenic shock when employing pulsatile bypass support [6,7]. Contrary to this, some studies have demonstrated that pulsatile flow does not exhibit superior effects compared to non-pulsatile flow [8]. The 2019 EACTS/EACTA/EBCP guidelines for CPB in adult cardiac surgery advised that patients at a high risk of renal and lung complications might benefit from pulsatile perfusion. However, the guidelines acknowledged the limited evidence supporting its effectiveness [9]. There have been ongoing concerns regarding the possibility of harmful effects associated with pulsatile perfusion modes. Some evidence suggests that pulsatile flow may elevate hemolysis levels in certain CPB circuits [10].

Although there is increasing evidence supporting pulsatile perfusion, the debate over its superiority compared to the non-pulsatile method in CPB persists. Previous systematic reviews on this subject included fewer than ten studies each, focused mainly on renal function or mortality, and did not explore subgroup differences. Moreover, they were published in 2014 and 2015, leaving a considerable body of newer research unaccounted for. Our review addresses these limitations by incorporating 33 randomized controlled trials up to 2024, assessing a broader set of clinical and biochemical outcomes, and performing subgroup analyses based on age group, surgery type, and pump type.

This systematic review and meta-analysis aim to evaluate and compare a range of clinical and biochemical outcomes in patients undergoing CPB with pulsatile versus non-pulsatile flow. Specifically, we assessed renal function (serum creatinine levels, creatinine clearance, estimated glomerular filtration rate [eGFR], blood urea nitrogen [BUN], and incidence of acute renal failure), liver function (alanine aminotransferase [ALT] and aspartate aminotransferase [AST]), lactate levels, intubation time, ICU stay, total hospital stay, use of inotropic agents, and overall mortality.

## Materials and methods

### 2.1. Protocol documentation

This study adopted the preferred reporting items of systematic reviews and meta-analysis (PRISMA) statement guidelines [11] in adherence to the Cochrane Handbook of Systematic Reviews [12]. The protocol for this meta-analysis was registered in PROSPERO with ID: **CRD42024529847.**

### 2.2. Data sources & search strategy

Two reviewers searched PubMed, Cochrane Central Register of Controlled Trials, Scopus, Web of Science, and EMBASE from inception up to February 2024 without redirections using the keywords “cardiopulmonary bypass,” “pulsatile flow,” and “pulsatile perfusion.” More details about the search strategy are outlined in (S1 Table).

### 2.3. Eligibility criteria

Randomized controlled trials (RCTs) that met the following PICO criteria were included in the meta-analysis: population (P): patients who underwent cardiopulmonary bypass (CPB); intervention (I): pulsatile flow; comparison (C): non-pulsatile flow; and outcomes (O): our primary outcome was serum creatinine levels, while secondary outcomes included ALT, AST, BUN, creatinine clearance, eGFR, lactate levels, ICU stay, hospital stay, intubation time, inotropic use, and the incidence of acute renal failure, and all-cause mortality.

Conference abstracts, observational studies, non-randomized studies, review articles, and animal studies were excluded.

### 2.4. Study selection

Study selection was done by using Covidence software to delete the duplicates. Three reviewers (M.R.A., A.M., and A.A.S.A) screened the titles/abstracts and then the full texts in accordance with the previously mentioned eligibility criteria. The authors resolved any disagreements during the screening or extraction via discussion, and if the dispute continued, a senior author (B.A) would be asked to resolve it.

### 2.5. Data extraction

Ten reviewers (M.R.A, A.M, A.A.S.A, A.M.A, M.A.A, A.S, M.M.A, D.A, S.E, A.H, and B.K) extracted the data using an Excel sheet encompassing study characteristics (study design, country, total participants, pediatrics or adults, operation type, and methods of pulsatility); baseline characteristics of the included studies’ population (number of patients in each group, age, gender, current smoking, diabetes mellitus, liver enzymes, and renal function markers), and study outcomes as previously described.

### 2.6. Risk of bias assessment

The assessors used the Cochrane Risk of Bias tool (ROB-2) to assess the risk of bias in the included RCTs [13]. We judged each study as “low risk,” “high risk,” or “some concerns”.

### 2.7. Statistical analysis

The analysis was done with R statistical software (v4.3.2). For dichotomous variables, we estimated the pooled risk ratio (RR) and 95% confidence interval (CI), and for continuous variables, we calculated mean differences with a 95% CI. The chi-square test and I^2^ were used to evaluate the statistical heterogeneity. When the heterogeneity was deemed significant (p 0.1 or I^2 ^> 60%), we employed a random effects model; otherwise, we used a fixed effects model. We performed a subgroup analysis based on the age group. Finally, a funnel plot was performed to check the possible publication bias via the Comprehensive Meta-analysis Software. During performing the sensitivity analysis, we used the leave-one method, in which we omit a separate study each time and check the reflection of this act on the overall heterogeneity, if the heterogeneity is resolved via omitting a certain study, we mention it and mention the CI and other related statistics after the omission, if not, we mention that heterogeneity could not be resolved via sensitivity analysis.

## Results

### 3.1. Search results

**Fig 1:**
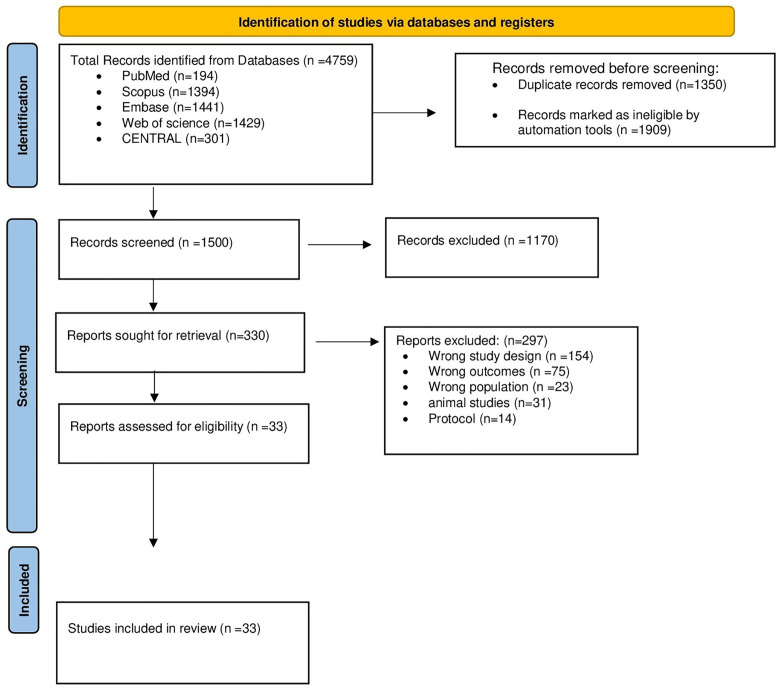
PRISMA flow chart of the screening process.

The search yielded 4759 studies across different databases, with 1500 records remaining for title and abstract screening after omitting 1350 duplicates, and 1909 were considered ineligible by Covidence automation tools. After screening abstracts and titles, 1170 records were excluded, leaving 330 studies eligible for full-text screening. Of them, 297 were excluded due to the reasons mentioned in **Fig 1**, and 33 studies were included in this review. The PRISMA flow diagram search, selection, and exclusion reasons are outlined in (Fig 1**).**

### 3.2. Characteristics of Included Studies

A total of 33 RCTs [14–46] were included in this review. Six of these studies explored interventions in the pediatric population [15–18,44,46]. The remaining twenty-seven studies focused on adult patients. A complete overview and baseline characteristics of all included RCTs are presented in (Tables 1 and 2).

**Table 1:** Characteristics of included studies.

Study ID	Study design	Country	Total participants	Pediatrics or Adults	Operation type	Methods of pulsatility
**Akçevin et al. 2010 [15]**	RCT	United states	289	Pediatrics	Surgery for repair of congenital heart disease	Roller pump
**Alkan et al. 2006 [16]**	RCT	Turkey	50	Pediatrics	Surgery for repair of congenital heart disease	Roller pump
**Alkan et al. 2007 [17]**	RCT	Turkey	215	Pediatrics	Surgery for repair of congenital heart disease	Roller pump
**Alkan et al. 2013 [18]**	RCT	USA	89	Pediatrics	Surgery for repair of congenital heart disease	Roller pump
**Amouzegar et al. 2017 [19]**	RCT	Iran	70	Adults	CABG	Roller pump
**Badner et al. 1992 [20]**	RCT	Canada	100	Adults	CABG	Roller pump
**Borulu et al. 2020 [21]**	RCT	Turkey	40	Adults	CABG	Roller pump
**Dodonov et al. 2021 [22]**	RCT	Italy	52	Adults	Aortic valve replacement	Centrifugal pump
**Driessen et al. 1995 [23]**	RCT	Belgium	38	Adults	CABG	Centrifugal pump
**Engels et al. 2014 [24]**	RCT	Italy	37	Adults	Aortic valve replacement	Centrifugal pump
**Graßler et al. 2019 [25]**	RCT	Germany	40	Adults	CABG	N/A
**Gu et al. 2011 [26]**	RCT	Netherlands	32	Adults	CABG	Centrifugal pump
**Jiang et al. 2021 [27]**	RCT	China	60	Adults	Valve disease	Roller pump
**Kocakulak et al. 2005 [28]**	RCT	Turkey	40	Adults	Variable cardiac surgeries	Roller pump
**Louagie et al. 1992 [29]**	RCT	Belgium	100	Adults	CABG	Roller pump
**Mali et al. 2021 [30]**	RCT	Iran	50	Adults	CABG	Roller pump
**Mohammadzadeh et al. 2013 [31]**	RCT	Iran	80	Adults	Variable cardiac surgeries	Roller pump
**Murkin et al. 1995 [32]**	RCT	Canada	316	Adults	CABG	Roller pump
**O’Neil et al. 2012 [33]**	RCT	Canada	20	Adults	Variable cardiac surgeries	Roller pump
**O’Neil et al. 2018 [34]**	RCT	Canada	20	Adults	CABG OR valve repair	Roller pump
**Onorati et al. 2007 [35]**	RCT	Italy	100	Adults	CABG	Intra-aortic ballon pump
**Onorati et al. 2009 a [36]**	RCT	Italy	158	Adults	CABG	Intra-aortic ballon pump
**Onorati et al. 2009 b [37]**	RCT	Italy	80	Adults	CABG	Intra-aortic ballon pump
**Poswal et al. 2004 [38]**	RCT	India	100	Adults	CABG	Roller pump
**Serraino et al. 2012 [39]**	RCT	Italy	501	Adults	CABG	Roller pump
**Sezai et al. 2005 [40]**	RCT	Japan	24	Adults	CABG	Roller pump
**Shahandashti et al. 2023 [41]**	RCT	Iran	90	Adults	CABG	Roller pump
**Tarcan et al. 2004 [42]**	RCT	Turkey	19	Adults	CABG	Roller pump
**Ulus et al. 2023 [43]**	RCT	Turkey	12	Adults	CABG	Roller pump
**Ündar et al. 2022 [44]**	RCT	USA	159	Pediatrics	Surgery for repair of congenital heart disease	Roller pump
**Zavareh et al. 2018 [45]**	RCT	Iran	68	Adults	CABG	N/A
**Zhao et al. 2011 [46]**	RCT	China	40	Pediatrics	Tetralogy of Fallot	Roller pump

**(RCT: randomized controlled trial; N/A: not available; CABG: coronary artery bypass graft).**

**Table 2:** Baseline Characteristics of Included Patients.

Study ID	Number of patients in each group		Age (Years), Mean (SD)		Gender (Male), N. (%)		Current smoking, N. (%)		Diabetes mellitus, N. (%)		Liver enzymes, Mean (SD)				Renal function markers, Mean (SD)			
	Pulsatile	Non-pulsatile	Pulsatile	Non-pulsatile	Pulsatile	Non-pulsatile	Pulsatile	Non-pulsatile	Pulsatile	Non-pulsatile	ALT (IU/L)		AST (IU/L)		Creatinine		BUN	
											Pulsatile	Non-pulsatile	Pulsatile	Non-pulsatile	Pulsatile	Non-pulsatile	Pulsatile	Non-pulsatile
**Adademir et al. 2012 [14]**	42	43	61.3 (10.5)	57.5 (9.0)	31 (73.8)	28 (65.1)	23 (54.8)	22 (48.8)	10 (23.8)	13 (30.2)	N/A	N/A	N/A	N/A	0.94 (0.13)	0.94 (0.17)	18.36 (7.73)	17.36 (8.63)
**Akçevin et al. 2010 [15]**	208	81	N/A	N/A	96	27	N/A	N/A	N/A	N/A	N/A	N/A	N/A	N/A	N/A	N/A	N/A	N/A
**Alkan et al. 2006 [16]**	25	25	N/A	N/A	16 (64)	17 (68)	N/A	N/A	N/A	N/A	12.76 (7.8)	11.68 (3.84)	21.2 (15.5)	18.32 (7.20)	0.24 (0.12)	0.31 (0.31)	N/A	N/A
**Alkan et al. 2007 [17]**	151	64	N/A	N/A	69 (45.7)	21(32.8)	N/A	N/A	N/A	N/A	14.4 (3.7)	14.68 (3.8)	21.8 (9.41)	22.3 (8.20)	0.28 (0.16)	0.26 (0.11)	N/A	N/A
**Alkan et al. 2013 [18]**	58	31	N/A	N/A	28 (48.3)	17 (54.8)	N/A	N/A	N/A	N/A	12.76 (7.8)	11.68 (3.84)	21.2 (15.5)	18.32 (7.20)	0.24 (0.12)	0.31 (0.31)	N/A	N/A
**Amouzegar et al. 2017 [19]**	36	36	61.67 (8.94)	62.00 (7.39)	26 (72.2)	29 (80.6)	N/A	N/A	10 (27.8)	19 (52.8)	N/A	N/A	N/A	N/A	1.18 (0.25)	1.36 (0.53)	17.72 (5.81)	20.86 (9.75)
**Badner et al. 1992 [20]**	49	51	61.07 (11.48)	60.23 (11.3)	42 (85.7)	44 (88)	N/A	N/A	N/A	N/A	N/A	N/A	N/A	N/A	1.14 (0.4)	1.2 (0.35)	16.5 (5.6)	17.4 (6.4)
**Borulu et al. 2020 [21]**	20	20	60.45 (7.79)	57.40 (7.59)	17 (85)	17 (85)	N/A	N/A	3 (15)	5 (25)	N/A	N/A	N/A	N/A	N/A	N/A	N/A	N/A
**Dodonov et al. 2021 [22]**	27	25	80 (3.1)	80 (3.4)	11 (41)	14 (56)	N/A	N/A	7 (26)	5 (22)	N/A	N/A	N/A	N/A	1.03 (0.24)	1.12 (0.35)	N/A	N/A
**Driessen et al. 1995 [23]**	19	19	63 (7)	63 (9)	15 (78.9)	16 (84.2)	10 (52.6)	6 (31.6)	N/A	N/A	N/A	N/A	N/A	N/A	N/A	N/A	N/A	N/A
**Engels et al. 2014 [24]**	18	19	80 (3)	79 (3)	9 (45)	11 (58)	N/A	N/A	6 (33)	3 (18)	N/A	N/A	N/A	N/A	N/A	N/A	N/A	N/A
**Graßler et al. 2019 [25]**	21	19	64.5 (10.3)	66.2 (8.8)	18 (86)	16 (84)	N/A	N/A	7 (33)	7 (37)	N/A	N/A	N/A	N/A	N/A	N/A	N/A	N/A
**Gu et al. 2011 [26]**	16	16	69 (7)	63 (11)	15 (94)	12 (75)	N/A	N/A	N/A	N/A	N/A	N/A	N/A	N/A	N/A	N/A	N/A	N/A
**Jiang et al. 2021 [27]**	30	30	56 (4.1)	58 (9.2)	14 (46.7)	12 (40)	N/A	N/A	N/A	N/A	N/A	N/A	N/A	N/A	N/A	N/A	189.65 (58.17)	193.41 (61.22)
**Kocakulak et al. 2005 [28]**	20	20	54.3 (15.8)	53.1 (11.2)	16 (80)	15 (75)	N/A	N/A	N/A	N/A	N/A	N/A	N/A	N/A	1.36 (0.8)	1.61 (1.4)	N/A	N/A
**Louagie et al. 1992 [29]**	50	50	62.8 (1.0)	58.9 (1.4)	44 (88)	44 (88)	N/A	N/A	N/A	N/A	18.1 (2.2)	19.3 (2.3)	14 (1)	16 (1)	1.1 (0.0)	1.1 (0.0)	16.1 (0.65)	15.77 (0.65)
**Mali et al. 2021 [30]**	25	25	59.04 (11.50)	57.52 (10.51)	14 (56)	15 (60)	14 (56)	10 (40)	12 (48)	10 (40)	N/A	N/A	N/A	N/A	1.12 (0.35)	0.98 (0.22)	20.08 (9.10)	15.08 (3.82)
**Mohammadzadeh et al. 2013 [31]**	40	40	61.4 (12.3)	60.1 (8.7)	20 (50)	21 (52.5)	N/A	N/A	N/A	N/A	N/A	N/A	N/A	N/A	1.12 (0.31)	1.20 (0.31)	N/A	N/A
**Murkin et al. 1995 [32]**	158	158	60.55 (2.5)	61.3 (2.5)	136 (86.1)	128 (81)	N/A	N/A	28 (17.7)	30 (19)	N/A	N/A	N/A	N/A	N/A	N/A	N/A	N/A
**O’Neil et al. 2012 [33]**	10	10	75.3 (7.0)	69.7 (11.8)	5 (50)	7 (70)	N/A	N/A	4 (40)	2 (20)	N/A	N/A	N/A	N/A	N/A	N/A	N/A	N/A
**O’Neil et al. 2018 [34]**	10	10	72.4 (14.5)	74.6 (9.9)	5 (50)	7 (70)	N/A	N/A	2 (20)	2 (20)	N/A	N/A	N/A	N/A	N/A	N/A	N/A	N/A
**Onorati et al. 2007 [35]**	50	50	67.6 (9.7)	68.3 (9.5)	46 (92)	47(94)	N/A	N/A	28 (56)	31 (62)	N/A	N/A	N/A	N/A	1.23 (0.38)	1.24 (0.37)		
**Onorati et al. 2009 a [36]**	87	71	69.4 (0.89)	67.5 (1.07)	82 (94.3)	65 (91.5)	37(42.5)	22 (31)	48 (55.2)	29 (40.8)	17.8 (1.07)	21.3 (1.56)	21.4 (0.85)	23.9 (1.32)	1.1 (0.03)	1.1 (0.04)	N/A	N/A
**Onorati et al. 2009 b [37]**	40	40	75.5 (4.8)	74.6 (4.2)	32 (80)	36 (90)	17 (42.5)	21 (52.5)	17 (42.5)	23 (57.5)	29.8 (6.24)	35.94 (7.81)	39.57 (5.11)	41.25 (8.13)	1.08 (0.48)	1.10 (0.53)	N/A	N/A
**Poswal et al. 2004 [38]**	50	50	54.5 (8.6)	56.4 (7.7)	N/A	N/A	N/A	N/A	N/A	N/A	N/A	N/A	N/A	N/A	1.11 (0.18)	1.05 (0.21)	28.54 (7.6)	27.3(9.3)
**Serraino et al. 2012 [39]**	231	270	69.7 (0.95)	68.2 (1.12)	176 (76.2)	213 (78.9)	97 (42.0)	110 (40.7)	121 (52.4)	132 (48.9)	19.2 (1.23)	20.8 (1.74)	22.1 (0.94)	24.1 (1.27)	1.0 (0.1)	1.1 (0.2)	N/A	N/A
**Sezai et al. 2005 [40]**	12	12	66.7 (6.1)	64.6 (6.3)	10 (83)	10 (83)	3 (25)	3 (25)	N/A	N/A	N/A	N/A	N/A	N/A	N/A	N/A	N/A	N/A
**Shahandashti et al. 2023 [41]**	58	29	59.34 (5.72)	61.38 (4.14)	42 (72.4)	22 (75.9)	10 (17.2)	4 (13.8)	24 (41.3)	15 (51.7)	20.36 (9.51)	20.97 (10.07)	19.48 (7.01)	19.76 (8.35)	1.09 (0.25)	1.11 (0.19)	18.38 (7.6)	17.78 (5.36)
**Tarcan et al. 2004 [42]**	10	9	50.8 (8.6)	52.3 (12.6)	10	9	N/A	N/A	0	0	N/A	N/A	N/A	N/A	N/A	N/A	N/A	N/A
**Ulus et al. 2023 [43]**	6	6	66.0 (8.5)	59.7 (8.0)	6 (100)	6 (100)	N/A	N/A	N/A	N/A	13.6 (0.5)	25.7 (19.6)	13.3 (3.0)	21.1 (11.9)	0.96 (0.11)	0.84 (0.17)	33.5 (12.5)	36.4 (10.5)
**Ündar et al. 2022 [44]**	83	76	2.51 (0.34)	3.31 (0.43)	46 (55.4)	35 (46.1)	N/A	N/A	N/A	N/A	N/A	N/A	N/A	N/A	N/A	N/A	N/A	N/A
**Zavareh et al. 2018 [45]**	35	33	61.89 (8.98)	62.06 (7.64)	25 (71.4)	27 (81.8)	N/A	N/A	10 (28.6)	16 (48.5)	23.37 (12.79)	25.19 (13.68)	26.49 (10.44)	31.25 (16.44)	N/A	N/A	N/A	N/A
**Zhao et al. 2011 [46]**	20	20	1.37 (0.37)	1.29 (0.32)	N/A	N/A	N/A	N/A	N/A	N/A	N/A	N/A	N/A	N/A	N/A	N/A	N/A	N/A

**(SD: standard deviation; N: number; ALT: alanine transferase; AST: aspartate transferase; N/A: not available; BUN: blood urea nitrogen; IU/L: international unit per liter).**

### 3.3. Risk of bias and quality assessment

**Fig 2:**
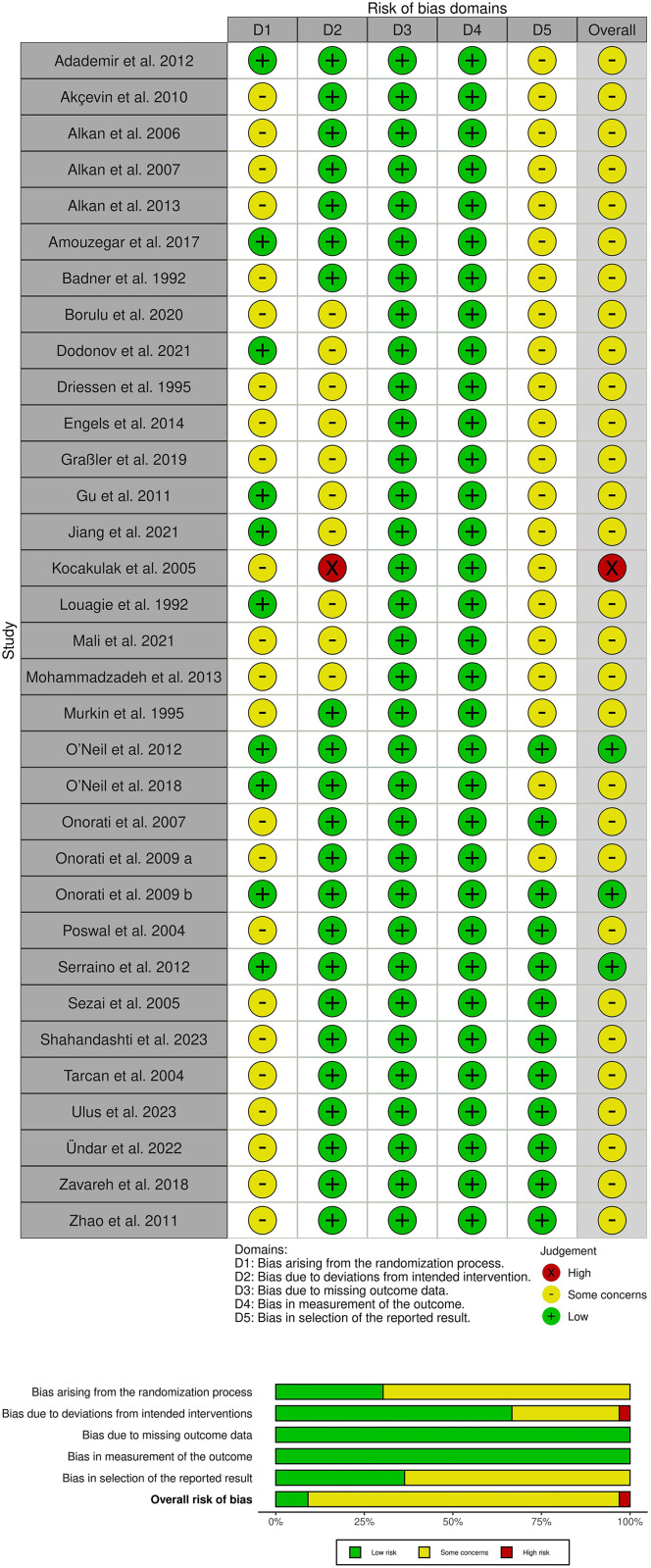
Summary of risk of bias. (A- review authors’ judgments about each risk of bias item for each included study, B- review authors’ judgments about each risk of bias item presented as percentages across all included studies).

Three studies [33,37,39] showed an overall low risk of bias. While twenty-nine studies [14–27,29–32,34–36,38,40–46] showed overall some concerns. On the other hand, one study [28] yielded an overall high risk. More detailed information can be obtained from (Fig 2**).** The quality of evidence is illustrated via GRADE instructions (Table 3**).**

**Table 3:** GRADE evidence profile.

Certainty assessment							Certainty
№ of studies	Study design	Risk of bias	Inconsistency	Indirectness	Imprecision	Other considerations

**Alanine transaminase**							
11	Randomized trials	Not serious	Very serious^a^	Serious^b^	Not serious	None	⨁○○○ Very low
**Aspartate transaminase**							
11	Randomized trials	Not serious	Very serious^a^	Not serious	Not serious	None	⨁⨁○○ Low
**BUN**							
12	Randomized trials	Not serious	Serious^c^	Not serious	Not serious	None	⨁⨁⨁○ Moderate
**Creatinine**							
20	Randomized trials	Not serious	Very serious^a^	Not serious	Not serious	None	⨁⨁○○ Low
**Hospital stay (days)**							
15	Randomized trials	Not serious	Very serious^a^	Serious^b^	Not serious	None	⨁○○○ Very low
**Acute renal failure**							
6	Randomized trials	Not serious	Not serious	Not serious	Serious^d^	None	⨁⨁⨁○ Moderate
**ICU stay days**							
14	Randomized trials	Not serious	Very serious^a^	Serious^b^	Not serious	None	⨁○○○ Very low
**Creatinine clearance**							
8	Randomized trials	Not serious	Very serious^a^	Not serious	Not serious	None	⨁⨁○○ Low
**eGFR**							
4	Randomized trials	Not serious	Very serious^a^	Not serious	Serious^d^	None	⨁○○○ Very low
**Inotropic use**							
6	Randomized trials	Not serious	Not serious	Not serious	Not serious	None	⨁⨁⨁⨁ High
**Intubation time (hours)**							
16	Randomized trials	Not serious	Very serious^a^	Not serious	Not serious	Publication bias strongly suspected^f^	⨁○○○ Very low
**Lactate**							
11	Randomized trials	Not serious	Very serious^a^	Not serious	Not serious	None	⨁⨁○○ Low
**Mortality**							
6	Randomized trials	Not serious	Not serious	Not serious	Not serious	None	⨁⨁⨁⨁ High

**CI: confidence interval, BUN; blood urea nitrogen, ICU; intensive care unit, eGFR; estimated glomerular filtration rate.**

**Explanations:**

**a. Very high heterogeneity**

**b. Significant difference between different age subgroups**

**c. High heterogeneity**

**d. Wide CI**

**e. small sample size**

**f. Egger test < 0.05**

### 3.4. Renal functions

#### 3.4.1. Creatinine level.

**Fig 3:**
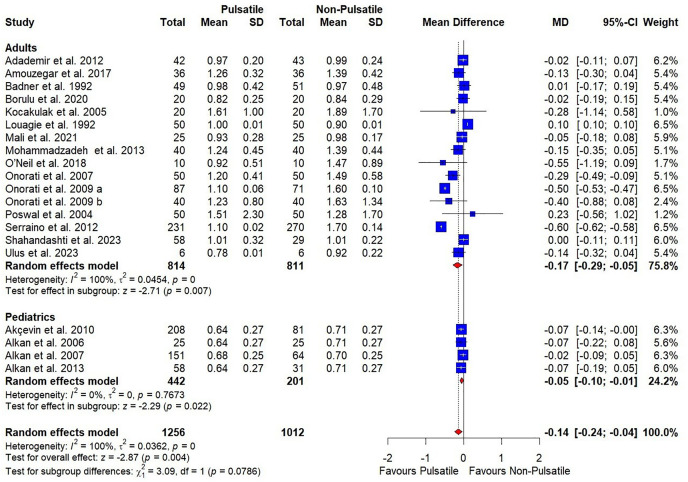
Forest plots illustrate the mean difference in creatinine level with subgroup analysis based on patients’ age.

Low-certainty evidence showed that pulsatile perfusion significantly decreased creatinine levels compared to the non-pulsatile perfusion [MD = −0.14, 95% CI (−0.24, −.04), P = 0.004] (Table 3, Fig 3**).** The pooled analysis was heterogeneous (I^2^ = 100%, P = 0), and which sensitivity analysis could not resolve.

Subgroup analysis based on age showed no difference between adults and pediatric patients (P = 0.08) and failed to resolve the heterogeneity (Fig 3**).**

Subgroup analysis based on surgery types showed no difference between any of the subgroups including CABG and congenital heart disease surgeries and didn’t resolve the heterogeneity (P = 0.16) (S1 Fig**).**

Subgroup analysis based on pump types showed that pulsatile perfusion significantly decreased creatinine levels compared to the non-pulsatile perfusion in both intra-aortic balloon pump and roller pump; [MD = −0.42, 95% CI (−0.59, −0.26), P < 0.001] and [MD = −0.10, 95% CI (−0.19, −0.00), P = 0.04], respectively, with a significant superiority in intra-aortic balloon pump group (P = 0007) (S2 Fig**).**

#### 3.4.2. Creatinine clearance.

**Fig 4:**
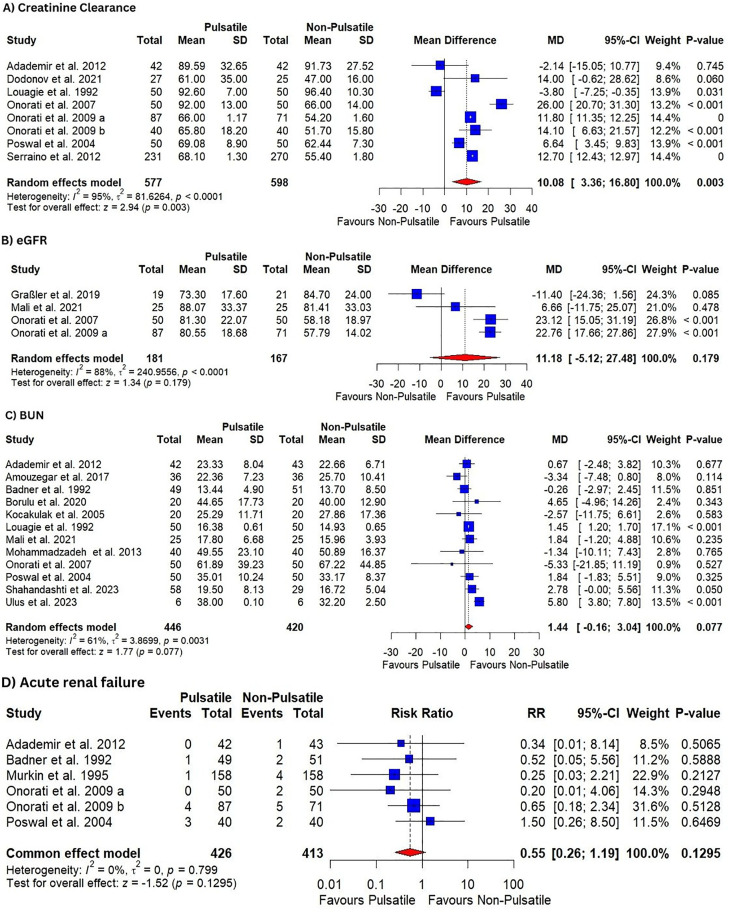
Forest plots illustrate the mean difference in; (A) creatinine clearance, (B) eGFR, (C) BUN, and (D) shows relative risk in ARF.

Low-certainty evidence showed that pulsatile perfusion significantly increased creatinine clearance compared to the non-pulsatile perfusion [MD = 10.08, 95% CI (3.36, 16.80), P = 0.003] (Table 3, Fig 4**).** The pooled analysis was heterogeneous (I^2^ = 95%, P < 0.0001), which sensitivity analysis could not resolve. All the included studies were in the adult population.

Subgroup analysis based on the type of surgery showed no significant change regarding the results or heterogeneity (S3 Fig**).** However, subgroup analysis based on type of pump showed that pulsatile perfusion significantly increased creatinine clearance compared to the non-pulsatile perfusion in studies that used intra-aortic balloon pump [MD = 17.17, 95% CI (8.37, 25.96), P < 0.001], but not for roller pump [MD = 4.09.17, 95% CI (−3.90, 12.08), P = 0.3]. The results were heterogenous in both subgroups (I^2^ = 93%, P < 0.0001), (I^2^ = 97%, P < 0.0001), respectively (S4 Fig**).**

#### 3.4.3. eGFR.

Very low-certainty evidence showed that there was no difference in pulsatile perfusion compared to the non-pulsatile perfusion in eGFR level [MD = 11.18, 95% CI (−5.12, 27.48), P = 0.179] (Table 3, Fig 4**).** The pooled analysis was heterogeneous (I^2^ = 88%, P < 0.0001), which was best resolved by omitting the Graßler et al. 2019 study, (I^2^ = 29%), with a significant effect on the results [MD = 22.02, 95% CI (17.82, 26.22), P < 0.01] (S5 Fig**).** All the included studies were in the adult population using GABG surgery. Subgroup analysis based on the pump type showed that pulsatile perfusion significantly increased eGFR compared to the non-pulsatile perfusion in intra-aortic balloon pump [MD = 22.86, 95% CI (18.55, 27.18), P < 0.001] with results being homogenous (I^2^ = 0%, P = 0.94) (S6 Fig**).**

#### 3.4.4. BUN.

Moderate-certainty evidence showed that there was no difference in pulsatile perfusion compared to the non-pulsatile perfusion in BUN level [MD = 1.44, 95% CI (−0.16, 3.04), P = 0.08] (Table 3, Fig 4). The pooled analysis was heterogeneous (I^2^ = 61%, P < 0.0031), which was best resolved by omitting the Ulus et al. 2023 study, (I^2^ = 1%), with a significant effect on the results [MD = 1.43, 95% CI (1.18, 1.67), P < 0.01] (S7 Fig). All the included studies were in the adult population. Subgroup analysis based on the type of surgery showed no significant difference between CABG and other cardiac surgeries (P = 0.29) (S8 Fig). Subgroup analysis based on the pump type showed no difference between intra-aortic balloon pump and roller pump (P = 0.42) (S9 Fig)

#### 3.4.5. Acute renal failure.

Moderate-certainty evidence showed that there was no difference in pulsatile perfusion compared to the non-pulsatile perfusion in ARF incidence [RR = 0.55, 95% CI (0.26, 1.19), P = 0.13] (Table 3, Fig 4**).** The pooled analysis was homogenous (I^2^ = 0%, P = 0.8); all the included studies were in the adult population. Subgroup analysis based on pump type didn’t show significant difference between the two groups and the results were homogenous across all the subgroups (S10 Fig)

### 3.5. Time-related outcomes

#### 3.5.1. Hospital stay.

**Fig 5:**
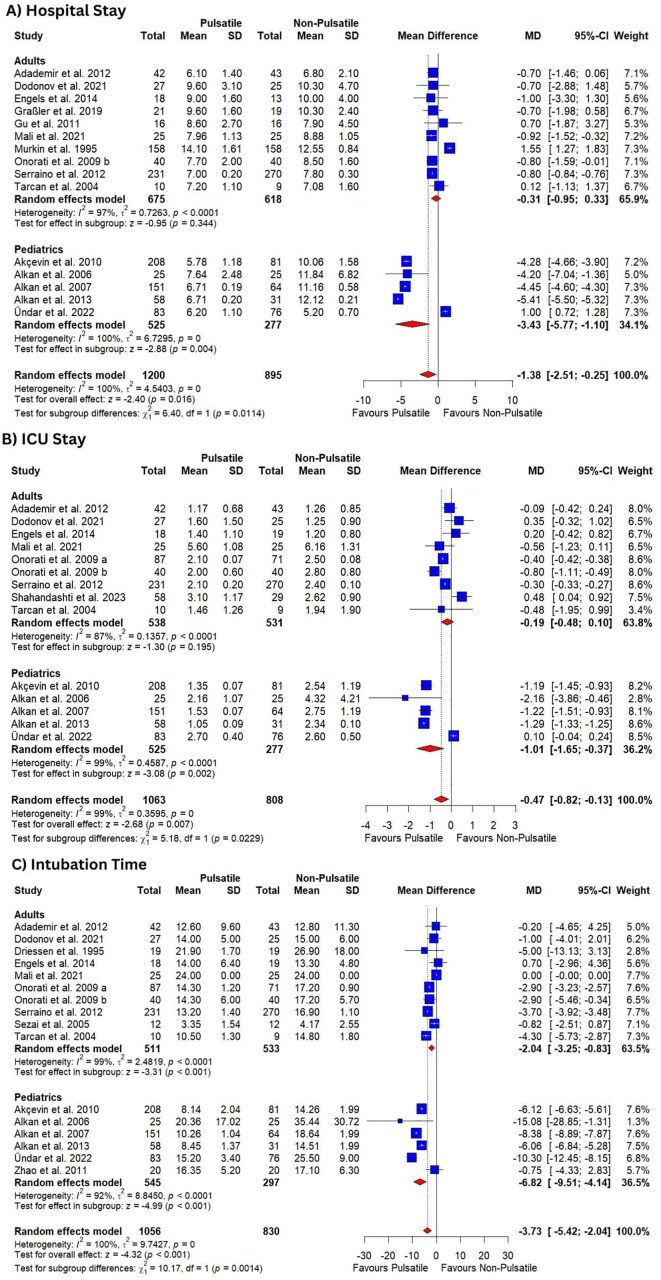
Forest plots illustrate the mean difference in; (A) hospital stay, (B) ICU stay, (C) intubation time; with subgroup analysis.

Very low-certainty evidence showed that pulsatile perfusion significantly decreased hospital stay compared to the non-pulsatile perfusion [MD = −1.38, 95% CI (−2.51, −0.25), P = 0.016] (Table 3, Fig 5**).** The pooled analysis was heterogeneous (I^2^ = 100%, P = 0), which sensitivity analysis could not resolve.

Subgroup analysis based on age showed that pulsatile perfusion significantly decreased hospital stay compared to non-pulsatile perfusion in pediatric patients [MD = −3.43, 95% CI (−5.77, −1.10), P = 0.004]. On the other hand, there was no difference in the adults’ group [MD = −0.31, 95% CI (−0.95, 0.33), P = 0.34].

Subgroup analysis based on the type of surgery revealed that surgeries for repair of congenital heart disease was the only surgery in which pulsatile perfusion significantly decreased hospital stay compared to the non-pulsatile perfusion [MD = −4.70, 95% CI (−5.36, −4.05), P < 0.001]. The result was heterogenous (I^2^ = 98%, P < 0.0001) (S11 Fig**).**

Subgroup based on type of pump showed that centrifugal pump was associated with no significant difference between pulsatile perfusion compared to the non-pulsatile perfusion in hospital stay [MD = −0.42, 95% CI (−1.77, 0.93), P = 0.54] with result being homogenous (I^2^ = 0%, P = 0.59), while roller was associated with significantly shorter hospital stay in pulsatile perfusion group [MD = −1.76, 95% CI (−3.34, −0.18)] with heterogenous results (I^2^ = 100%, P = 0) (S12 Fig**).**

#### 3.5.2. ICU stay.

Very low-certainty evidence showed that pulsatile perfusion reduced ICU stay compared to the non-pulsatile perfusion [MD = −0.47, 95% CI (−0.82, −0.13), P = 0.007] (Table 3, Fig 5**).** The pooled analysis was heterogeneous (I^2^ = 99%, P = 0), which sensitivity analysis could not resolve.

In adults, subgroup analysis showed that there was no difference in pulsatile perfusion compared to the non-pulsatile perfusion in ICU stay [MD = −0.19, 95% CI (−0.48, 0.10), P = 0.195] (Fig 5**).** The pooled analysis was heterogeneous (I^2^ = 87%, P < 0.0001).

However, the effect of pulsatile perfusion was significant in pediatrics [MD = −1.01, 95% CI (−1.65, −0.37), P = 0.002] (Fig 5**).** The pooled analysis was heterogeneous (I^2^ = 99%, P < 0.0001). Further subgroup analysis based on the type of surgery revealed that surgeries for repair of congenital heart disease was the only surgery in which pulsatile perfusion significantly decreased ICU stay compared to the non-pulsatile perfusion [MD = −1.29, 95% CI (−1.33, −1.25), P < 0.001]. The result was homogenous (I^2^ = 0%, P = 0.62) (S13 Fig**).** Meanwhile, subgroup analysis based on type of pump showed no difference between pulsatile perfusion with centrifugal pump compared to the non-pulsatile perfusion in ICU stay [MD = 0.27, 95% CI (−0.19, 0.72), P < 0.001] with result being homogenous (I^2^ = 0%, P = 0.74), while roller and intra-aortic balloon pumps were associated with significantly shorter ICU stay in pulsatile perfusion group with heterogenous results (S14 Fig**).**

#### 3.5.3. Intubation time.

Very low-certainty evidence showed that pulsatile perfusion significantly decreased intubation time compared to the non-pulsatile perfusion [MD = −3.73, 95% CI (−5.42, −2.04), P < 0.001] (Table 3, Fig 5**).** The pooled analysis was heterogeneous (I^2^ = 100%, P = 0), which sensitivity analysis could not resolve.

In adults, subgroup analysis revealed that pulsatile perfusion significantly decreased intubation time compared to the non-pulsatile perfusion [MD = −2.04, 95% CI (−3.25, −.083), P = 0.001] (Fig 5), the pooled analysis was heterogeneous (I^2^ = 99%, P < 0.0001), which sensitivity analysis could not resolve. Furthermore, the effect of pulsatile perfusion was significant in pediatrics [MD = −6.82, 95% CI (−9.51, −4.14), P = 0.022] (Fig 5**),** the pooled analysis was heterogeneous (I^2^ = 92%, P < 0.0001).

Further subgroup analysis based on the type of surgery revealed that aortic valve replacement was the only surgery that showed no significant difference between the two groups [MD = −0.31, 95% CI (−2.64, 2.01), P = 0.79] (S15 Fig**).** Meanwhile, centrifugal pump was the only type to show no significant difference between pulsatile perfusion compared to the non-pulsatile perfusion in intubation time [MD = −0.67, 95% CI (−2.91, 1.57), P = 0.57] with result being homogenous (I^2^ = 0%, P = 0.43) (S16 Fig**).**

### 3.6. Liver function tests

#### 3.6.1. Alanine aminotransferase (ALT) level:.

Very low-certainty evidence showed that there was no difference between pulsatile and non-pulsatile perfusion in ALT level [MD = −3.40, 95% CI (−7.25, 0.44), P = 0.083**] (**Table 3, S17 Fig**).** The pooled analysis was heterogeneous (I^2^ = 99%, P < 0.0001), which sensitivity analysis could not resolve. However, in adults, pulsatile perfusion significantly decreased ALT levels compared to the non-pulsatile perfusion [MD = −5.81, 95% CI (−11.14, −0.47), P = 0.033] (S17 Fig**).** The pooled analysis was heterogeneous (I^2^ = 99%, P < 0.0001), which sensitivity analysis could not resolve. In pediatric patients, however, there was no significant difference in ALT levels between the two groups [MD = 0.90, 95% CI (−1.19, 2.98), P = 0.399] (S17 Fig**),** the pooled analysis was homogenous (I^2^ = 0%, P = 0.998). Subgroup analysis based on surgery types and pump types partially resolved the heterogeneity. Pulsatile perfusion significantly decreased ALT levels in CABG surgeries and with intra-aortic balloon pump compared to non-pulsatile perfusion; [MD = −5.81, 95% CI (−11.14, −0.47), P < 0.0001] and [MD = −13.79, 95% CI (−27.49, −0.10), P = 0.048], respectively. The pooled analysis was heterogenous; (I^2^ = 99%, P < 0.0001) and (I^2^ = 96%, P < 0.0001), respectively (S18, S19 Fig**).**

#### 3.6.2. Aspartate Aminotransferase (AST) level.

Low-certainty evidence showed that there was no difference in pulsatile perfusion compared to the non-pulsatile perfusion in AST level [MD = −0.66, 95% CI (−10.52, 9.19), P = 0.895] (Table 3, S17 Fig**).** The pooled analysis was heterogeneous (I^2^ = 98%, P < 0.0001), which sensitivity analysis could not resolve.

In adults, subgroup analysis showed that there was no difference in pulsatile perfusion compared to the non-pulsatile perfusion in AST level [MD = −4.74, 95% CI (−17.90, 8.41), P = 0.480] (S17 Fig**).** The pooled analysis was heterogeneous (I^2^ = 99%, P < 0.0001), which sensitivity analysis could not resolve. However, pulsatile perfusion marginally increased AST levels compared to the non-pulsatile method in pediatrics [MD = 9.03, 95% CI (0.07, 17.99), P = 0.048] (S17 Fig**),** the pooled analysis was homogenous (I^2^ = 0%, P = 0.965). Further subgroup analysis based on the type of pump showed no significant change in both groups regarding the results or heterogeneity (S20 Fig**).** However, subgroup analysis based on surgery type showed that pulsatile perfusion increased AST levels compared to the non-pulsatile method in surgeries for repair of congenital heart disease [MD = 9.03, 95% CI (0.07, 17.99), P = 0.048]. The pooled analysis was homogenous (I^2^ = 0%, P = 0.96) (S21 Fig**).**

### 3.7. Other outcomes

#### 3.7.1. Lactate level:.

Low-certainty evidence showed that pulsatile perfusion significantly decreased lactate levels compared to the non-pulsatile perfusion [MD = −8.21, 95% CI (−13.16, −3.25), P = 0.001] (Table 3, S22 Fig**).** The pooled analysis was heterogeneous (I^2^ = 99%, P = 0), which sensitivity analysis could not resolve.

In adults, subgroup analysis revealed that pulsatile perfusion significantly decreased lactate levels compared to the non-pulsatile perfusion [MD = −6.14, 95% CI (−11.01, −1.28), P = 0.013] (S22 Fig**),** the pooled analysis was heterogeneous (I^2^ = 99%, P < 0.0001), which sensitivity analysis could not resolve.

Furthermore, pulsatile perfusion significantly decreased lactate levels compared to the non-pulsatile perfusion in pediatrics [MD = −11.03, 95% CI (−21.44, −0.62), P = 0.038] (S22 Fig**),** the pooled analysis was heterogeneous (I^2^ = 100%, P < 0.0001).

Subgroup analysis based on the type of surgery or pumps showed no significant change regarding the results or heterogeneity (S23 Fig) and (S24 Fig**),** retrospectively.

#### 3.7.2. Mortality.

High-certainty evidence showed no difference in mortality between pulsatile and non-pulsatile perfusion [RR = 0.69, 95% CI (0.34, 1.41), P = 0.30] (Table 3, S22 Fig). The pooled analysis was Homogeneous (I^2^ = 0%, P = 0.30). Subgroup analysis based on the type of surgery and pump didn’t show a significant difference between the two groups, and the results were homogenous across all the subgroups (S25 Fig) and (S26 Fig), retrospectively.

#### 3.7.3. Inotropic use.

High-certainty evidence showed that there was no difference in pulsatile perfusion compared to the non-pulsatile perfusion in inotropic use [RR = 1.09, 95% CI (0.91, 1.30), P = 0.37] (Table 3, S22 Fig**).** The pooled analysis was homogenous (I^2^ = 30%, P = 0.21). Subgroup analysis based on the type of surgery and pump didn’t show significant difference between the two groups and the results were homogenous across all the subgroups (S27, S28 Fig).

### 3.8. Funnel plots

We performed several funnel plots to check for publication bias for several outcomes including, creatinine level outcome (Egger test = p-value = 0.1632), BUN level outcome (Egger test = p-value = 0.9268), ALT level outcome (Egger test = p-value = 0.5918), AST level outcome (Egger test = p-value = 0.4669), hospital stay outcome (Egger test = p-value = 0.7717), ICU stay outcome (Egger test = p-value = 0.8143), intubation time outcome (Egger test = p-value = 0.0072), and lactate level outcome (Egger test = p-value = 0.3818) (S29-S36 Fig**).**

## Discussion

Our study presents a comprehensive systematic review and meta-analysis aimed at exploring differences in outcomes among patients undergoing CPB with either pulsatile or non-pulsatile flow. Our results revealed that pulsatile perfusion was associated with significantly reduced creatinine and lactate levels and increased creatinine clearance. It also shortened hospital, and ICU stays compared to non-pulsatile perfusion. However, there were no observable differences between the two groups in glomerular filtration rate (GFR), blood urea nitrogen (BUN) levels, incidence of acute renal failure, inotropic usage, or mortality rates. In adults, subgroup analysis showed no significant difference except for ALT level, lactate level, and intubation time, which were significantly decreased. However, in pediatric patients, pulsatile perfusion significantly reduced hospital stays, ICU stays, lactate levels, and intubation time, and increased AST levels.

Our kidneys are very sensitive to low pulse pressure. Renin secretion increases as a result, causing elevated systemic venous resistance and redistribution of intra-renal blood flow. This is why non-pulsatile perfusion could aggravate renal function [47–49]. These mechanisms are supported by our own findings and align with prior studies. We found—with low certainty of evidence—that pulsatile perfusion significantly reduced creatinine levels and increased creatinine clearance. Other renal function parameters were not statistically significant in our analysis, with very low certainty of evidence for eGFR and moderate certainty for both BUN and acute renal failure. The presence of substantial heterogeneity and low certainty of evidence in most renal outcomes highlights the complexity of drawing firm conclusions about the superiority of pulsatile over non-pulsatile perfusion. The heterogeneity in eGFR outcome was resolved by omitting Graßler et al. (2019), likely due to key differences in their study design. Unlike other trials (e.g., Onorati 2009, Mali 2021), which used conventional CPB in higher-risk patients, Graßler et al. employed minimal invasive ECMO (MiECC) in low-risk elective CABG patients, potentially masking pulsatility’s benefits. Additionally, Graßler measured eGFR only at discharge, missing early postoperative renal changes detected in studies with serial assessments. Nevertheless, our findings are consistent with two prior meta-analyses [50,51] that specifically investigated the effect of pulsatile perfusion on renal function, both of which reported significantly increased creatinine clearance. Notably, Nam et al. [51], which included only adult patients, also found reduced creatinine levels and fewer acute renal failure events.

Yan et al. [52] conducted a comprehensive meta-analysis including 32 studies and 2,568 patients to investigate the effects of pulsatile flow on postoperative recovery in adult cardiac surgery with CPB. They concluded that hospital and ICU stays were significantly shorter in the pulsatile perfusion group. In contrast, a 2024 RCT by Patel et al. [53], which focused solely on pediatric patients undergoing cyanotic and acyanotic congenital heart surgery, found no significant difference between groups in hospital and ICU length of stay or intubation time.

Interestingly, the type of surgery performed during CPB significantly influenced renal outcomes and ICU/hospital length of stay. Based on our subgroup analysis, pulsatile perfusion significantly lowered creatinine levels and lactate levels, shortened both ICU and hospital stays, and increased AST levels in patients undergoing surgery for congenital heart disease. These findings align with experimental and physiological studies suggesting that pediatric patients, particularly those undergoing congenital repairs, may be more susceptible to the adverse effects of non-physiological flow and may benefit more from the hemodynamic mimicry provided by pulsatile perfusion [54–56]. On the other hand, other surgical groups—such as those undergoing CABG—showed significant improvements in creatinine levels, lactate levels, mortality, inotropic use, and ALT levels, and the results were often heterogeneous. This variability may stem from underlying comorbidities or technical differences in the application of pulsatile flow in adult surgeries [57,58].

In addition, the type of pump also influences renal and hepatic function. Several studies have shown that IABP-generated pulsatile flow is more consistently associated with renal, hepatic, and pulmonary benefits than other forms of pulse delivery, likely due to higher levels of shear-derived hemodynamic energy and less loss across the CPB circuit [35,39]. Our subgroup analysis supports these findings, showing that patients who received IABP-based pulsatile perfusion exhibited significantly improved creatinine clearance, eGFR, as well as reduced ICU stay, and intubation time, creatinine, lactate, and ALT levels compared to those treated with roller or centrifugal pumps. This may be partly explained by IABP’s ability to enhance forward flow and more effectively mimic physiological pulse pressure, thereby improving end-organ perfusion and reducing ischemic injury. Moreover, the enhanced shear stress generated by IABP pulsatility has been shown to stimulate the endothelial glycocalyx and nitric oxide production, both of which contribute to improved vascular tone and organ perfusion [5].

As for other outcomes, our analysis revealed that pulsatile perfusion significantly lowered lactate levels compared to non-pulsatile perfusion, although this was based on low-certainty evidence and characterized by severe heterogeneity that persisted despite sensitivity analyses. There were no differences, by contrast, between groups for mortality or need for inotropic support, based upon high-certainty evidence. These findings further reinforce that caution must be exercised upon interpreting these results and reflect how difficult it is to define one approach to perfusion as clinically superior to the other.

Our study has some limitations. The pooled results exhibited substantial heterogeneity across most renal outcomes, with the exception of ARF. This variability is likely driven by differences in study design, patient populations (e.g., pediatric vs. adult), perfusion methods, and clinical settings. Variations in the definition of pulsatility, perfusion techniques, types of surgery, and postoperative management protocols may further contribute to the inconsistent findings.

Clinically, this heterogeneity limits the generalizability of our results and cautions against a one-size-fits-all approach to adopting pulsatile perfusion. The variability observed may reflect the need to tailor perfusion strategies to specific patient populations or clinical contexts. Additionally, although pulsatile flow demonstrated promising benefits, the observed heterogeneity suggests that these effects are likely context dependent.

## Conclusion

Although pulsatile perfusion reduced creatinine levels and significantly increased creatinine clearance, the other renal functional parameters of the two groups were constant. Lactate levels were low in the pulsatile group. Additionally, pulsatile flow significantly reduced hospital and ICU stay and intubation time without affecting mortality rates. On the other hand, ALT and AST were still unchanged between pulsatile and non-pulsatile flow. These findings suggest that pulsatile perfusion holds promise for improving patient outcomes, especially in high-risk groups like those with pre-existing kidney dysfunction. However, the absence of significant differences in mortality suggests that these benefits may not universally translate to long-term clinical gains. This study provides a foundation for future efforts, bridging the gap between physiological benefits and practical applications in cardiac surgery.

## Supporting information

S1 File PRISMA checklist: A checklist that contains the preferred reporting items for systematic reviews and meta-analyses. (DOCX)

S2 File Data Extraction Sheet: A sheet that contains the data that we extracted from the included studies. (XLSX)

S3 File Risk of Bias Assessment (ROB 2): A document that contains our detailed risk of bias assessment using Cochrane’s risk of bias tool 2. (DOCX)

S4 File Screening Sheet: A sheet that contains the full screening process that we used to include or exclude any study. (XLSX)

S1 Table Search strategy. (DOCX)

S1 Fig Subgroup analysis of Creatinine level based on surgery type. (JPG)

S2 Fig Subgroup analysis of Creatinine level based on pump types. (JPG)

S3 Fig Subgroup analysis of Creatinine clearance based on surgery type. (JPG)

S4 Fig Subgroup analysis of Creatinine clearance based on pump type. (JPG)

S5 Fig Sensitivity analysis for eGFR. (JPG)

S6 Fig Subgroup analysis of eGFR based on pump type. (JPG)

S7 Fig Sensitivity analysis for BUN. (JPG)

S8 Fig Subgroup analysis of BUN based on surgery type. (JPG)

S9 Fig Subgroup analysis of BUN based on pump type. (JPG)

S10 Fig Subgroup analysis of Acute renal failure based on pump type. (JPG)

S11 Fig Subgroup analysis of Hospital Stay based on surgery type. (JPG)

S12 Fig Subgroup analysis of Hospital Stay based on pump type. (JPG)

S13 Fig Subgroup analysis of ICU Stay based on surgery type. (JPG)

S14 Fig Subgroup analysis of ICU Stay based on pump type. (JPG)

S15 Fig Subgroup analysis of Intubation time based on surgery type. (JPG)

S16 Fig Subgroup analysis of Intubation time based on pump type. (JPG)

S17 Fig Forest plot of liver function tests. (JPG)

S18 Fig Subgroup analysis of ALT levels based on surgery type. (JPG)

S19 Fig Subgroup analysis of ALT levels based on pump type. (JPG)

S20 Fig Subgroup analysis of AST levels based on pump type. (JPG)

S21 Fig Subgroup analysis of AST levels based on surgery type. (JPG)

S22 Fig Forest plot of other outcomes (Lactate, Mortality, and Inotropic use). (JPG)

S23 Fig Subgroup analysis of Lactate level based on surgery type. (JPG)

S24 Fig Subgroup analysis of Lactate level based on pump type. (JPG)

S25 Fig Subgroup analysis of mortality rate based on surgery type. (JPG)

S26 Fig Subgroup analysis of mortality based on pump type. (JPG)

S27 Fig Subgroup analysis of Inotropic use based on surgery type. (JPG)

S28 Fig Subgroup analysis of Inotropic use based on pump type. (JPG)

S29 Fig Funnel plot of Creatinine level outcome (Egger test = p-value = 0.1632). (JPG)

S30 Fig Funnel plot of BUN level outcome (Egger test = p-value = 0.9268). (JPG)

S31 Fig Funnel plot of ALT level outcome (Egger test = p-value = 0.5918). (JPG)

S32 Fig Funnel plot of AST level outcome (Egger test = p-value = 0.4669). (JPG)

S33 Fig Funnel plot of hospital stay outcome (Egger test = p-value = 0.7717). (JPG)

S34 Fig Funnel plot of ICU stay outcome (Egger test = p-value = 0.8143). (JPG)

S35 Fig Funnel plot of intubation time outcome (Egger test = p-value = 0.0072). (JPG)

S36 Fig Funnel plot of lactate level outcome (Egger test = p-value = 0.3818). (JPG)
