# Supplementary material for: The impact of pulsatile vs. non-pulsatile perfusion in patients undergoing cardiopulmonary bypass: A comprehensive systematic review and meta-analysis of 33 randomized controlled trials
Source: PLoS One. 2025 Oct 14;20(10):e0333495. doi: 10.1371/journal.pone.0333495 (PMC12520390; doi:10.1371/journal.pone.0333495)
Supplement: S3 File — (DOCX) [file pone.0333495.s003.docx]

| **Unique ID** | 1 | **Study ID** | Adademir2012 | **Assessor** | Ali Ashraf & Salem Elshenawy |
| --- | --- | --- | --- | --- | --- |
| **Ref or Label** |  | **Aim** | assignment to intervention (the 'intention-to-treat' effect) |  |  |
| **Experimental** | Pulsatile perfusion (Group P) | **Comparator** | non-pulsatile perfusion (Group NP) | **Source** | Journal article(s) |
| **Outcome** | IL-18 levels-Urinary NGAL levels | **Results** | IL-18 levels measured at 12 h after CPB were significantly lower in Group P, compared to Group NP (p<0.05). Urinary NGAL levels measured at 2 and 12 h were higher in Group NP. | **Weight** | 1 |
| **Domain** | **Signalling question** | | | **Response** | **Comments** |
| **Bias arising from the randomization process** | 1.1 Was the allocation sequence random? | | | Y | Eligible patients were randomized to receive pulsatile  (Group P, n = 42) and non-pulsatile (Group NP, n = 43) flow  CPB by lottery, drawing from sealed envelopes containing  the group assignment. |
|  | 1.2 Was the allocation sequence concealed until participants were enrolled and assigned to interventions? | | | Y |  |
|  | 1.3 Did baseline differences between intervention groups suggest a problem with the randomization process? | | | N |  |
|  | **Risk of bias judgement** | | | **Low** |  |
| **Bias due to deviations from intended interventions** | 2.1.Were participants aware of their assigned intervention during the trial? | | | N | Eligible patients were randomized to receive pulsatile  (Group P, n = 42) and non-pulsatile (Group NP, n = 43) flow  CPB by lottery, drawing from sealed envelopes containing  the group assignment. |
|  | 2.2.Were carers and people delivering the interventions aware of participants' assigned intervention during the trial? | | | NI |  |
|  | 2.3. If Y/PY/NI to 2.1 or 2.2: Were there deviations from the intended intervention that arose because of the experimental context? | | | N |  |
|  | 2.4 If Y/PY to 2.3: Were these deviations likely to have affected the outcome? | | | NA |  |
|  | 2.5. If Y/PY/NI to 2.4: Were these deviations from intended intervention balanced between groups? | | | NA |  |
|  | 2.6 Was an appropriate analysis used to estimate the effect of assignment to intervention? | | | PY | Statistical analyses were performed using SPSS 16.0 package program. The data were expressed as mean ± standard deviation and percentage. Chi-square test was used to analyze qualitative data and t-test was used for quantitative variables in independent groups. One-way  ANOVA test with Bonferroni correction was used in intra group comparison of continuous variables, and the independent samples t-test was used in comparison of renal functions between the groups. The level of statistical significance was set at p<0.05. |
|  | 2.7 If N/PN/NI to 2.6: Was there potential for a substantial impact (on the result) of the failure to analyse participants in the group to which they were randomized? | | | NA |  |
|  | **Risk of bias judgement** | | | **Low** |  |
| **Bias due to missing outcome data** | 3.1 Were data for this outcome available for all, or nearly all, participants randomized? | | | Y | 85 consecutive patients with normal preoperative renal function were prospectively enrolled in the study |
|  | 3.2 If N/PN/NI to 3.1: Is there evidence that result was not biased by missing outcome data? | | | NA |  |
|  | 3.3 If N/PN to 3.2: Could missingness in the outcome depend on its true value? | | | NA |  |
|  | 3.4 If Y/PY/NI to 3.3: Is it likely that missingness in the outcome depended on its true value? | | | NA |  |
|  | **Risk of bias judgement** | | | **Low** |  |
| **Bias in measurement of the outcome** | 4.1 Was the method of measuring the outcome inappropriate? | | | N | Results: There was no significant difference between the groups in terms of perioperative renal function tests. IL-18 levels measured at 12 h after CPB were significantly lower in Group P, compared to Group NP (p<0.05). Urinary NGAL levels measured at 2 and 12 h were higher in Group NP; however, the difference was insignificant. |
|  | 4.2 Could measurement or ascertainment of the outcome have differed between intervention groups? | | | N |  |
|  | 4.3 Were outcome assessors aware of the intervention received by study participants? | | | N |  |
|  | 4.4 If Y/PY/NI to 4.3: Could assessment of the outcome have been influenced by knowledge of intervention received? | | | NA |  |
|  | 4.5 If Y/PY/NI to 4.4: Is it likely that assessment of the outcome was influenced by knowledge of intervention received? | | | NA |  |
|  | **Risk of bias judgement** | | | **Low** |  |
| **Bias in selection of the reported result** | 5.1 Were the data that produced this result analysed in accordance with a pre-specified analysis plan that was finalized before unblinded outcome data were available for analysis? | | | NI |  |
|  | 5.2 ... multiple eligible outcome measurements (e.g. scales, definitions, time points) within the outcome domain? | | | N |  |
|  | 5.3 ... multiple eligible analyses of the data? | | | N |  |
|  | **Risk of bias judgement** | | | **Some concerns** |  |
| **Overall bias** | **Risk of bias judgement** | | | **Some concerns** |  |
|  |  |  |  |  |  |
|  |  |  |  |  |  |
| **Unique ID** | 2 | **Study ID** | Akçevin2010 | **Assessor** | Ali Ashraf & Salem Elshenawy |
| **Ref or Label** |  | **Aim** | assignment to intervention (the 'intention-to-treat' effect) |  |  |
| **Experimental** | pulsatile perfusion group | **Comparator** | nonpulsatile perfusion group | **Source** | Journal article(s) |
| **Outcome** | Study parameters included total drainage, mean urine output in the intensive care unit (ICU), intubation time, duration of ICU and hospital stay, the need for inotropic support, pre- and postoperative enzyme levels(ALT [alanine aminotransaminase] and AST [aspartate aminotransaminase]), c-reactive protein, lactate, albumin, blood count (leukocytes, hematocrit, platelets), creatinine levels, and thyroid hormones (thyroid stimulating hormone [TSH], FT3 [free triiodothyronine], FT4 [free thyroxine]). | **Results** | Group P, compared to Group NP, required significantly less postoperative inotropic support (number of agents 1.6 0.08 vs. 2 0.12, P = 0.0015), shorter intubation period, higher urine output in ICU, and a shorter duration of ICU and hospital stay. Although there were no significant differences in creatinine or enzyme levels, blood counts (white blood cells, hematocrit, and platelets), or drainage. | **Weight** | 1 |
| **Domain** | **Signalling question** | | | **Response** | **Comments** |
| **Bias arising from the randomization process** | 1.1 Was the allocation sequence random? | | | Y | As a result, 289 children undergoing open heart surgery for repair of congenital heart disease fulfilling the inclusion criteria were included in this prospective study and randomly assigned to either the pulsatile perfusion group (Group P, n = 208) or the nonpulsatile perfusion group (Group NP, n = 81). |
|  | 1.2 Was the allocation sequence concealed until participants were enrolled and assigned to interventions? | | | NI |  |
|  | 1.3 Did baseline differences between intervention groups suggest a problem with the randomization process? | | | N |  |
|  | **Risk of bias judgement** | | | **Some concerns** |  |
| **Bias due to deviations from intended interventions** | 2.1.Were participants aware of their assigned intervention during the trial? | | | NI |  |
|  | 2.2.Were carers and people delivering the interventions aware of participants' assigned intervention during the trial? | | | NI |  |
|  | 2.3. If Y/PY/NI to 2.1 or 2.2: Were there deviations from the intended intervention that arose because of the experimental context? | | | N |  |
|  | 2.4 If Y/PY to 2.3: Were these deviations likely to have affected the outcome? | | | NA |  |
|  | 2.5. If Y/PY/NI to 2.4: Were these deviations from intended intervention balanced between groups? | | | NA |  |
|  | 2.6 Was an appropriate analysis used to estimate the effect of assignment to intervention? | | | PY | Results are presented as means standard error of means. In statistical comparisons of two groups (P and NP), independent samples t-test either for equal variances or nonequal variances were used when the distribution was normal. For the non-Gaussian distributions, Mann–Whitney U-test was applied. For the comparison of categorical data, chi-square test was used. Statistical significance level was considered as P < 0.05. All analyses were performed by using SPSS for Windows (version 11.5) statistical software package (Chicago, IL, USA). |
|  | 2.7 If N/PN/NI to 2.6: Was there potential for a substantial impact (on the result) of the failure to analyse participants in the group to which they were randomized? | | | NA |  |
|  | **Risk of bias judgement** | | | **Low** |  |
| **Bias due to missing outcome data** | 3.1 Were data for this outcome available for all, or nearly all, participants randomized? | | | Y | Group P, compared to Group NP, required significantly less postoperative inotropic support (number of agents 1.6 0.08 vs. 2 0.12, P = 0.0015), shorter intubation period, higher urine output in ICU, and a shorter duration of ICU and hospital stay (Table 3). Although there were no significant differences in creatinine or enzyme levels, blood counts (white blood cells, hematocrit, and platelets), or drainage amounts between two groups, lower lactate levels and higher albumin levels were observed in Group P |
|  | 3.2 If N/PN/NI to 3.1: Is there evidence that result was not biased by missing outcome data? | | | NA |  |
|  | 3.3 If N/PN to 3.2: Could missingness in the outcome depend on its true value? | | | NA |  |
|  | 3.4 If Y/PY/NI to 3.3: Is it likely that missingness in the outcome depended on its true value? | | | NA |  |
|  | **Risk of bias judgement** | | | **Low** |  |
| **Bias in measurement of the outcome** | 4.1 Was the method of measuring the outcome inappropriate? | | | N | Although there were no significant differences in creatinine or enzyme levels, blood counts (white blood cells, hematocrit, and platelets), or drainage amounts between two groups, lower lactate levels and higher albumin levels were observed in Group P |
|  | 4.2 Could measurement or ascertainment of the outcome have differed between intervention groups? | | | N |  |
|  | 4.3 Were outcome assessors aware of the intervention received by study participants? | | | NI |  |
|  | 4.4 If Y/PY/NI to 4.3: Could assessment of the outcome have been influenced by knowledge of intervention received? | | | N |  |
|  | 4.5 If Y/PY/NI to 4.4: Is it likely that assessment of the outcome was influenced by knowledge of intervention received? | | | NA |  |
|  | **Risk of bias judgement** | | | **Low** |  |
| **Bias in selection of the reported result** | 5.1 Were the data that produced this result analysed in accordance with a pre-specified analysis plan that was finalized before unblinded outcome data were available for analysis? | | | NI |  |
|  | 5.2 ... multiple eligible outcome measurements (e.g. scales, definitions, time points) within the outcome domain? | | | N |  |
|  | 5.3 ... multiple eligible analyses of the data? | | | N |  |
|  | **Risk of bias judgement** | | | **Some concerns** |  |
| **Overall bias** | **Risk of bias judgement** | | | **Some concerns** |  |
|  |  |  |  |  |  |
|  |  |  |  |  |  |
| **Unique ID** | 3 | **Study ID** | ALKAN2006 | **Assessor** | Ali Ashraf & Salem Elshenawy |
| **Ref or Label** |  | **Aim** | assignment to intervention (the 'intention-to-treat' effect) |  |  |
| **Experimental** | pulsatile perfusion group | **Comparator** | non-pulsatile perfusion group | **Source** | Journal article(s) |
| **Outcome** | Study parameters included intubation time, duration of intensive care unit (ICU) stay and hospital stay, need for inotropic support, preoperative and postoperative enzymes, creatinine, C-reactive protein, blood count, mean urine output, and total drainage | **Results** | Group P, compared with group NP, had significantly less inotropic support (number of agents, 1.48 ± 1.05 versus 2.44 ± 1.03, p =0.0015; dopamine, 6.48 ± 3.27 versus 10.3 ± 4.8 g/kg per minute, p =0.0023; dobutamine, 3.12 ± 6.55 versus 8.03 ± 9.1 g/kg per minute, p = 0.034), shorter intubation period (20.36 ± 17.02 versus 35.44 ± 30.72 hours, p = 0.038), and shorter duration of ICU stay (2.16 ± 1.07 versus 4.32 ± 4.21 days, p = 0.028) and hospital stay (7.64 ± 2.48 versus 11.84 ± 6.82 days, p = 0.007). There were no significant differences in creatinine, enzyme levels, or drainage amounts between the two groups. Higher urine output during CPB (553.6 ± 150.89 versus 465.8 ± 151.23 ml/d, p =0.045) and during the ICU period (658.8 ± 210.99 versus 528,2 ± 224.71 ml/d, p = 0.039) was observed in group P compared with group NP. | **Weight** | 1 |
| **Domain** | **Signalling question** | | | **Response** | **Comments** |
| **Bias arising from the randomization process** | 1.1 Was the allocation sequence random? | | | Y | Fifty consecutive pediatric patients undergoing open heart surgery for repair of congenital heart disease were prospectively entered into the study and randomly assigned to either the pulsatile perfusion group (group P) or the nonpulsatile perfusion group (group NP) |
|  | 1.2 Was the allocation sequence concealed until participants were enrolled and assigned to interventions? | | | NI |  |
|  | 1.3 Did baseline differences between intervention groups suggest a problem with the randomization process? | | | N |  |
|  | **Risk of bias judgement** | | | **Some concerns** |  |
| **Bias due to deviations from intended interventions** | 2.1.Were participants aware of their assigned intervention during the trial? | | | NI |  |
|  | 2.2.Were carers and people delivering the interventions aware of participants' assigned intervention during the trial? | | | NI |  |
|  | 2.3. If Y/PY/NI to 2.1 or 2.2: Were there deviations from the intended intervention that arose because of the experimental context? | | | N |  |
|  | 2.4 If Y/PY to 2.3: Were these deviations likely to have affected the outcome? | | | NA |  |
|  | 2.5. If Y/PY/NI to 2.4: Were these deviations from intended intervention balanced between groups? | | | NA |  |
|  | 2.6 Was an appropriate analysis used to estimate the effect of assignment to intervention? | | | PY | Results are presented as mean ± standard error of mean. In statistical comparisons of the two groups (P and NP), an independent-samples t test either for equal variances or nonequal variances was used when the distribution was normal. For the non-gaussian distributions, the Mann-Whitney U test was applied. For the comparison of categorical data, the chi-square test was used. A value of p < 0.05 was considered statistically significant. All analyses were performed by using SPPS for windows (version 11.5) statistical software package. The risk categories of surgical procedures were collapsed into four groups, with risk categories 4 to 6 being considered as category 4 and determined as the high-risk category |
|  | 2.7 If N/PN/NI to 2.6: Was there potential for a substantial impact (on the result) of the failure to analyse participants in the group to which they were randomized? | | | NA |  |
|  | **Risk of bias judgement** | | | **Low** |  |
| **Bias due to missing outcome data** | 3.1 Were data for this outcome available for all, or nearly all, participants randomized? | | | Y | Group P, compared with group NP, had significantly less inotropic support (number of agents, 1.48 ± 1.05 versus 2.44 ± 1.03, p =0.0015; dopamine, 6.48 ± 3.27 versus 10.3 ± 4.8 g/kg per minute, p =0.0023; dobutamine, 3.12 ± 6.55 versus 8.03 ± 9.1 g/kg per minute, p = 0.034), shorter intubation period (20.36 ± 17.02 versus 35.44 ± 30.72 hours, p = 0.038), and shorter duration of ICU stay (2.16 ± 1.07 versus 4.32 ± 4.21 days, p = 0.028) and hospital stay (7.64 ± 2.48 versus 11.84 ± 6.82 days, p = 0.007). There were no significant differences in creatinine, enzyme levels, or drainage amounts between the two groups. Higher urine output during CPB (553.6 ± 150.89 versus 465.8 ± 151.23 ml/d, p =0.045) and during the ICU period (658.8 ± 210.99 versus 528,2 ± 224.71 ml/d, p = 0.039) was observed in group P compared with group NP. |
|  | 3.2 If N/PN/NI to 3.1: Is there evidence that result was not biased by missing outcome data? | | | NA |  |
|  | 3.3 If N/PN to 3.2: Could missingness in the outcome depend on its true value? | | | NA |  |
|  | 3.4 If Y/PY/NI to 3.3: Is it likely that missingness in the outcome depended on its true value? | | | NA |  |
|  | **Risk of bias judgement** | | | **Low** |  |
| **Bias in measurement of the outcome** | 4.1 Was the method of measuring the outcome inappropriate? | | | N | Group P, compared with group NP, had significantly less inotropic support (number of agents, 1.48 ± 1.05 versus 2.44 ± 1.03, p =0.0015; dopamine, 6.48 ± 3.27 versus 10.3 ± 4.8 g/kg per minute, p =0.0023; dobutamine, 3.12 ± 6.55 versus 8.03 ± 9.1 g/kg per minute, p = 0.034), shorter intubation period (20.36 ± 17.02 versus 35.44 ± 30.72 hours, p = 0.038), and shorter duration of ICU stay (2.16 ± 1.07 versus 4.32 ± 4.21 days, p = 0.028) and hospital stay (7.64 ± 2.48 versus 11.84 ± 6.82 days, p = 0.007). There were no significant differences in creatinine, enzyme levels, or drainage amounts between the two groups. Higher urine output during CPB (553.6 ± 150.89 versus 465.8 ± 151.23 ml/d, p =0.045) and during the ICU period (658.8 ± 210.99 versus 528,2 ± 224.71 ml/d, p = 0.039) was observed in group P compared with group NP. |
|  | 4.2 Could measurement or ascertainment of the outcome have differed between intervention groups? | | | N |  |
|  | 4.3 Were outcome assessors aware of the intervention received by study participants? | | | NI |  |
|  | 4.4 If Y/PY/NI to 4.3: Could assessment of the outcome have been influenced by knowledge of intervention received? | | | N |  |
|  | 4.5 If Y/PY/NI to 4.4: Is it likely that assessment of the outcome was influenced by knowledge of intervention received? | | | NA |  |
|  | **Risk of bias judgement** | | | **Low** |  |
| **Bias in selection of the reported result** | 5.1 Were the data that produced this result analysed in accordance with a pre-specified analysis plan that was finalized before unblinded outcome data were available for analysis? | | | NI |  |
|  | 5.2 ... multiple eligible outcome measurements (e.g. scales, definitions, time points) within the outcome domain? | | | N |  |
|  | 5.3 ... multiple eligible analyses of the data? | | | N |  |
|  | **Risk of bias judgement** | | | **Some concerns** |  |
| **Overall bias** | **Risk of bias judgement** | | | **Some concerns** |  |
|  |  |  |  |  |  |
|  |  |  |  |  |  |
| **Unique ID** | 4 | **Study ID** | ALKAN2007 | **Assessor** | Ali Ashraf & Salem Elshenawy |
| **Ref or Label** |  | **Aim** | assignment to intervention (the 'intention-to-treat' effect) |  |  |
| **Experimental** | pulsatile perfusion group | **Comparator** | Nonpulsatile perfusion group | **Source** | Journal article(s) |
| **Outcome** | inotropic support, adrenalin, shorter intubation period, duration of intensive care unit (ICU), hospital stay, creatinine, enzyme levels, drainage amounts between two groups, lactate levels, albumin levels, urine output during ICU period | **Results** | Group P, compared with group NP, had significantly less inotropic support (number of agents 1.4 ± 0.07 vs. 2 ± 0.12, p = 0.0012; dopamine 7.14 ± 0.28 vs. 9.04 ± 0.42 g 32 kg 1 min 1 , p = 0.00025; dobutamine 4.12 ± 0.3 vs. 5.3 ± 0.6 g 32 kg 1 min 1 , p = 0.036), adrenalin (0.026 ± 0.005 vs. 0.046 ± 0.005 g 32 kg 1 min 1 , p = 0.021), shorter intubation period (10.26 ± 1.04 vs. 18.64 ± 1.99 hours, p = 0.021), shorter duration of intensive care unit (ICU) (1.53 ± 0.07 vs. 2.75 ± 1.19 days, p = 0.012), and hospital stay (6.71 ± 0.19 vs. 11.16 ± 0.58 days, p =0.002). Although there were no significant differences in either creatinine, enzyme levels, and drainage amounts between two groups, lower lactate levels 16.27 ± 2.02 vs. 24.66 ± 3.05 mg/dl, p = 0.00034), higher albumin levels (3.15 ± 0.03 vs. 2.95 ± 0.06 mg/dl, p = 0.046), and higher urine output (602.82 ± 21.5 vs. 505.55 ±34.2 ml/d, p = 0.016) during ICU period was observed in group P compared with group NP, respectively. | **Weight** | 1 |
| **Domain** | **Signalling question** | | | **Response** | **Comments** |
| **Bias arising from the randomization process** | 1.1 Was the allocation sequence random? | | | Y | Consecutive pediatric patients (n = 215) undergoing open heart surgery for repair of congenital heart disease were prospectively entered into the study and randomly assigned to either the pulsatile perfusion group (group P, n = 151) or the NP group (n = 64). |
|  | 1.2 Was the allocation sequence concealed until participants were enrolled and assigned to interventions? | | | NI |  |
|  | 1.3 Did baseline differences between intervention groups suggest a problem with the randomization process? | | | N |  |
|  | **Risk of bias judgement** | | | **Some concerns** |  |
| **Bias due to deviations from intended interventions** | 2.1.Were participants aware of their assigned intervention during the trial? | | | NI |  |
|  | 2.2.Were carers and people delivering the interventions aware of participants' assigned intervention during the trial? | | | NI |  |
|  | 2.3. If Y/PY/NI to 2.1 or 2.2: Were there deviations from the intended intervention that arose because of the experimental context? | | | N |  |
|  | 2.4 If Y/PY to 2.3: Were these deviations likely to have affected the outcome? | | | NA |  |
|  | 2.5. If Y/PY/NI to 2.4: Were these deviations from intended intervention balanced between groups? | | | NA |  |
|  | 2.6 Was an appropriate analysis used to estimate the effect of assignment to intervention? | | | PY | Results are presented as means ± standard error of means (SEMs). In statisitical comparisons of two groups (P and NP), independent samples t test either for equal variances or nonequal variances was used when the distribution was normal. For the nongaussian distributions, Mann-Whitney U test was applied. For the comparison of categorical data, chi-squared test was used. Statistical significance level was considered as p= 0.05. All analyses were performed by using SPPS for windows (version 11.5; SPPS Inc., Chicago, IL) statistical software package. |
|  | 2.7 If N/PN/NI to 2.6: Was there potential for a substantial impact (on the result) of the failure to analyse participants in the group to which they were randomized? | | | NA |  |
|  | **Risk of bias judgement** | | | **Low** |  |
| **Bias due to missing outcome data** | 3.1 Were data for this outcome available for all, or nearly all, participants randomized? | | | Y | Group P, compared with group NP, had significantly less inotropic support (number of agents 1.4 ± 0.07 vs. 2 ± 0.12, p = 0.0012; dopamine 7.14 ± 0.28 vs. 9.04 ± 0.42 g 32 kg 1 min 1 , p = 0.00025; dobutamine 4.12 ± 0.3 vs. 5.3 ± 0.6 g 32 kg 1 min 1 , p = 0.036), adrenalin (0.026 ± 0.005 vs. 0.046 ± 0.005 g 32 kg 1 min 1 , p = 0.021), shorter intubation period (10.26 ± 1.04 vs. 18.64 ± 1.99 hours, p = 0.021), shorter duration of intensive care unit (ICU) (1.53 ± 0.07 vs. 2.75 ± 1.19 days, p = 0.012), and hospital stay (6.71 ± 0.19 vs. 11.16 ± 0.58 days, p =0.002). Although there were no significant differences in either creatinine, enzyme levels, and drainage amounts between two groups, lower lactate levels 16.27 ± 2.02 vs. 24.66 ± 3.05 mg/dl, p = 0.00034), higher albumin levels (3.15 ± 0.03 vs. 2.95 ± 0.06 mg/dl, p = 0.046), and higher urine output (602.82 ± 21.5 vs. 505.55 ±34.2 ml/d, p = 0.016) during ICU period was observed in group P compared with group NP, respectively. |
|  | 3.2 If N/PN/NI to 3.1: Is there evidence that result was not biased by missing outcome data? | | | NA |  |
|  | 3.3 If N/PN to 3.2: Could missingness in the outcome depend on its true value? | | | NA |  |
|  | 3.4 If Y/PY/NI to 3.3: Is it likely that missingness in the outcome depended on its true value? | | | NA |  |
|  | **Risk of bias judgement** | | | **Low** |  |
| **Bias in measurement of the outcome** | 4.1 Was the method of measuring the outcome inappropriate? | | | N | Group P, compared with group NP, had significantly less inotropic support (number of agents 1.4 ± 0.07 vs. 2 ± 0.12, p = 0.0012; dopamine 7.14 ± 0.28 vs. 9.04 ± 0.42 g 32 kg 1 min 1 , p = 0.00025; dobutamine 4.12 ± 0.3 vs. 5.3 ± 0.6 g 32 kg 1 min 1 , p = 0.036), adrenalin (0.026 ± 0.005 vs. 0.046 ± 0.005 g 32 kg 1 min 1 , p = 0.021), shorter intubation period (10.26 ± 1.04 vs. 18.64 ± 1.99 hours, p = 0.021), shorter duration of intensive care unit (ICU) (1.53 ± 0.07 vs. 2.75 ± 1.19 days, p = 0.012), and hospital stay (6.71 ± 0.19 vs. 11.16 ± 0.58 days, p =0.002). Although there were no significant differences in either creatinine, enzyme levels, and drainage amounts between two groups, lower lactate levels 16.27 ± 2.02 vs. 24.66 ± 3.05 mg/dl, p = 0.00034), higher albumin levels (3.15 ± 0.03 vs. 2.95 ± 0.06 mg/dl, p = 0.046), and higher urine output (602.82 ± 21.5 vs. 505.55 ±34.2 ml/d, p = 0.016) during ICU period was observed in group P compared with group NP, respectively. |
|  | 4.2 Could measurement or ascertainment of the outcome have differed between intervention groups? | | | N |  |
|  | 4.3 Were outcome assessors aware of the intervention received by study participants? | | | NI |  |
|  | 4.4 If Y/PY/NI to 4.3: Could assessment of the outcome have been influenced by knowledge of intervention received? | | | PN |  |
|  | 4.5 If Y/PY/NI to 4.4: Is it likely that assessment of the outcome was influenced by knowledge of intervention received? | | | NA |  |
|  | **Risk of bias judgement** | | | **Low** |  |
| **Bias in selection of the reported result** | 5.1 Were the data that produced this result analysed in accordance with a pre-specified analysis plan that was finalized before unblinded outcome data were available for analysis? | | | NI |  |
|  | 5.2 ... multiple eligible outcome measurements (e.g. scales, definitions, time points) within the outcome domain? | | | N |  |
|  | 5.3 ... multiple eligible analyses of the data? | | | N |  |
|  | **Risk of bias judgement** | | | **Some concerns** |  |
| **Overall bias** | **Risk of bias judgement** | | | **Some concerns** |  |
|  |  |  |  |  |  |
|  |  |  |  |  |  |
| **Unique ID** | 5 | **Study ID** | ALKAN2013 | **Assessor** | Ali Ashraf & Salem Elshenawy |
| **Ref or Label** |  | **Aim** | assignment to intervention (the 'intention-to-treat' effect) |  |  |
| **Experimental** | pulsatile perfusion group | **Comparator** | Nonpulsatile perfusion group | **Source** | Journal article(s) |
| **Outcome** | The pulsatile group needed significantly less inotropic support (P < 0.05) and had lower lactate levels (P < 0.001), higher urine output (P < 0.01), and higher albumin levels (P < 0.05). In addition, the pulsatile group had less ICU (P < 0.01) and hospital stays (P < 0.001). We conclude that the use of pulsatile flow is a better option and should be considered for repair of the complex congenital heart defects. | **Results** | Group P, compared to Group NP, had significantly less inotropic support (number of agents 1.2 ± 0.1 vs. 1.73 ± 0.12, P = 0.0041; dopamine 6.02 ± 0.47 vs. 7.48 ± 0.49 mg/kg/min, P = 0.044; adrenalin 0.015 ± 0.004 vs. 0.038 ± 0.004 mg/kg/min, P = 0.025), less intubation time (8.45 ± 1.37 vs. 14.51 ± 1.99 h, P = 0.0034), less duration of ICU (1.05 ± 0.09 vs. 2.34 ± 0.10 days, P = 0.012) and hospital stay (6.71 ± 0.2 vs. 12.12 ± 0.21 days, P = 0.0028). | **Weight** | 1 |
| **Domain** | **Signalling question** | | | **Response** | **Comments** |
| **Bias arising from the randomization process** | 1.1 Was the allocation sequence random? | | | Y | Total of 89 consecutive pediatric patients undergoing open-heart surgery for repair of transposition of great arteries (TGA) and ventricular septal defect(VSD) were prospectively entered into the study and were randomly assigned to either the pulsatile perfusion group (Group P, n = 58) or the nonpulsatile perfusion group (Group NP, n = 31). |
|  | 1.2 Was the allocation sequence concealed until participants were enrolled and assigned to interventions? | | | NI |  |
|  | 1.3 Did baseline differences between intervention groups suggest a problem with the randomization process? | | | N |  |
|  | **Risk of bias judgement** | | | **Some concerns** |  |
| **Bias due to deviations from intended interventions** | 2.1.Were participants aware of their assigned intervention during the trial? | | | NI |  |
|  | 2.2.Were carers and people delivering the interventions aware of participants' assigned intervention during the trial? | | | NI |  |
|  | 2.3. If Y/PY/NI to 2.1 or 2.2: Were there deviations from the intended intervention that arose because of the experimental context? | | | N |  |
|  | 2.4 If Y/PY to 2.3: Were these deviations likely to have affected the outcome? | | | NA |  |
|  | 2.5. If Y/PY/NI to 2.4: Were these deviations from intended intervention balanced between groups? | | | NA |  |
|  | 2.6 Was an appropriate analysis used to estimate the effect of assignment to intervention? | | | PY | Group P, compared to Group NP, had significantly less inotropic support (number of agents 1.2 ± 0.1 vs. 1.73 ± 0.12, P = 0.0041; dopamine 6.02 ± 0.47 vs. 7.48 ± 0.49 mg/kg/min, P = 0.044; adrenalin 0.015 ± 0.004 vs. 0.038 ± 0.004 mg/kg/min, P = 0.025), less intubation time (8.45 ± 1.37 vs. 14.51 ± 1.99 h, P = 0.0034), less duration of ICU (1.05 ± 0.09 vs. 2.34 ± 0.10 days, P = 0.012) and hospital stay (6.71 ± 0.2 vs. 12.12 ± 0.21 days, P = 0.0028). |
|  | 2.7 If N/PN/NI to 2.6: Was there potential for a substantial impact (on the result) of the failure to analyse participants in the group to which they were randomized? | | | NA |  |
|  | **Risk of bias judgement** | | | **Low** |  |
| **Bias due to missing outcome data** | 3.1 Were data for this outcome available for all, or nearly all, participants randomized? | | | Y | Group P, compared to Group NP, had significantly less inotropic support (number of agents 1.2 ± 0.1 vs. 1.73 ± 0.12, P = 0.0041; dopamine 6.02 ± 0.47 vs. 7.48 ± 0.49 mg/kg/min, P = 0.044; adrenalin 0.015 ± 0.004 vs. 0.038 ± 0.004 mg/kg/min, P = 0.025), less intubation time (8.45 ± 1.37 vs. 14.51 ± 1.99 h, P = 0.0034), less duration of ICU (1.05 ± 0.09 vs. 2.34 ± 0.10 days, P = 0.012) and hospital stay (6.71 ± 0.2 vs. 12.12 ± 0.21 days, P = 0.0028). |
|  | 3.2 If N/PN/NI to 3.1: Is there evidence that result was not biased by missing outcome data? | | | NA |  |
|  | 3.3 If N/PN to 3.2: Could missingness in the outcome depend on its true value? | | | NA |  |
|  | 3.4 If Y/PY/NI to 3.3: Is it likely that missingness in the outcome depended on its true value? | | | NA |  |
|  | **Risk of bias judgement** | | | **Low** |  |
| **Bias in measurement of the outcome** | 4.1 Was the method of measuring the outcome inappropriate? | | | N | Group P, compared to Group NP, had significantly less inotropic support (number of agents 1.2 ± 0.1 vs. 1.73 ± 0.12, P = 0.0041; dopamine 6.02 ± 0.47 vs. 7.48 ± 0.49 mg/kg/min, P = 0.044; adrenalin 0.015 ± 0.004 vs. 0.038 ± 0.004 mg/kg/min, P = 0.025), less intubation time (8.45 ± 1.37 vs. 14.51 ± 1.99 h, P = 0.0034), less duration of ICU (1.05 ± 0.09 vs. 2.34 ± 0.10 days, P = 0.012) and hospital stay (6.71 ± 0.2 vs. 12.12 ± 0.21 days, P = 0.0028). |
|  | 4.2 Could measurement or ascertainment of the outcome have differed between intervention groups? | | | N |  |
|  | 4.3 Were outcome assessors aware of the intervention received by study participants? | | | NI |  |
|  | 4.4 If Y/PY/NI to 4.3: Could assessment of the outcome have been influenced by knowledge of intervention received? | | | PN |  |
|  | 4.5 If Y/PY/NI to 4.4: Is it likely that assessment of the outcome was influenced by knowledge of intervention received? | | | NA |  |
|  | **Risk of bias judgement** | | | **Low** |  |
| **Bias in selection of the reported result** | 5.1 Were the data that produced this result analysed in accordance with a pre-specified analysis plan that was finalized before unblinded outcome data were available for analysis? | | | NI |  |
|  | 5.2 ... multiple eligible outcome measurements (e.g. scales, definitions, time points) within the outcome domain? | | | N |  |
|  | 5.3 ... multiple eligible analyses of the data? | | | N |  |
|  | **Risk of bias judgement** | | | **Some concerns** |  |
| **Overall bias** | **Risk of bias judgement** | | | **Some concerns** |  |
|  |  |  |  |  |  |
|  |  |  |  |  |  |
| **Unique ID** | 6 | **Study ID** | Amouzegar2017 | **Assessor** | Ali Ashraf & Salem Elshenawy |
| **Ref or Label** |  | **Aim** | assignment to intervention (the 'intention-to-treat' effect) |  |  |
| **Experimental** | pulsatile | **Comparator** | Non-pulsatile | **Source** | Journal article(s) |
| **Outcome** | BUN and creatinine | **Results** | Both case and control groups had significantly increased BUN and creatinine. Increased creatinine on the first and second postoperative day was significantly higher in the control group. Furthermore, BUN increased on the second day in the control group and was significantly higher than the case group | **Weight** | 1 |
| **Domain** | **Signalling question** | | | **Response** | **Comments** |
| **Bias arising from the randomization process** | 1.1 Was the allocation sequence random? | | | Y | The same surgical team operated on all of the patients, who were randomly divided to 2 groups. Randomization was stratified using a random Table number. |
|  | 1.2 Was the allocation sequence concealed until participants were enrolled and assigned to interventions? | | | Y |  |
|  | 1.3 Did baseline differences between intervention groups suggest a problem with the randomization process? | | | N |  |
|  | **Risk of bias judgement** | | | **Low** |  |
| **Bias due to deviations from intended interventions** | 2.1.Were participants aware of their assigned intervention during the trial? | | | NI |  |
|  | 2.2.Were carers and people delivering the interventions aware of participants' assigned intervention during the trial? | | | NI |  |
|  | 2.3. If Y/PY/NI to 2.1 or 2.2: Were there deviations from the intended intervention that arose because of the experimental context? | | | N |  |
|  | 2.4 If Y/PY to 2.3: Were these deviations likely to have affected the outcome? | | | NA |  |
|  | 2.5. If Y/PY/NI to 2.4: Were these deviations from intended intervention balanced between groups? | | | NA |  |
|  | 2.6 Was an appropriate analysis used to estimate the effect of assignment to intervention? | | | PY | Descriptive statistics such as mean, standard deviation (SD), and percentages were calculated for the presented data. The Kolmogorov-Smirnov test was used for normality of the data. Chi-square test, repeated measure, analysis of variance (ANOVA), and t test were used for determining the association between variables. The SPSS 17 software was used to analyze the data. The significance level for all tests was considered less than 0.05. |
|  | 2.7 If N/PN/NI to 2.6: Was there potential for a substantial impact (on the result) of the failure to analyse participants in the group to which they were randomized? | | | NA |  |
|  | **Risk of bias judgement** | | | **Low** |  |
| **Bias due to missing outcome data** | 3.1 Were data for this outcome available for all, or nearly all, participants randomized? | | | Y | Seventy-two patients were evaluated for study participation. There was no significant difference between the 2 groups regarding gender, age, body mass index (BMI), DM, and hypertension |
|  | 3.2 If N/PN/NI to 3.1: Is there evidence that result was not biased by missing outcome data? | | | NA |  |
|  | 3.3 If N/PN to 3.2: Could missingness in the outcome depend on its true value? | | | NA |  |
|  | 3.4 If Y/PY/NI to 3.3: Is it likely that missingness in the outcome depended on its true value? | | | NA |  |
|  | **Risk of bias judgement** | | | **Low** |  |
| **Bias in measurement of the outcome** | 4.1 Was the method of measuring the outcome inappropriate? | | | N | The researchers assessed preoperative renal function of each patient by examining serum creatinine, BUN levels, and urine output. |
|  | 4.2 Could measurement or ascertainment of the outcome have differed between intervention groups? | | | N |  |
|  | 4.3 Were outcome assessors aware of the intervention received by study participants? | | | NI |  |
|  | 4.4 If Y/PY/NI to 4.3: Could assessment of the outcome have been influenced by knowledge of intervention received? | | | PN |  |
|  | 4.5 If Y/PY/NI to 4.4: Is it likely that assessment of the outcome was influenced by knowledge of intervention received? | | | NA |  |
|  | **Risk of bias judgement** | | | **Low** |  |
| **Bias in selection of the reported result** | 5.1 Were the data that produced this result analysed in accordance with a pre-specified analysis plan that was finalized before unblinded outcome data were available for analysis? | | | NI |  |
|  | 5.2 ... multiple eligible outcome measurements (e.g. scales, definitions, time points) within the outcome domain? | | | N |  |
|  | 5.3 ... multiple eligible analyses of the data? | | | N |  |
|  | **Risk of bias judgement** | | | **Some concerns** |  |
| **Overall bias** | **Risk of bias judgement** | | | **Some concerns** |  |
|  |  |  |  |  |  |
|  |  |  |  |  |  |
| **Unique ID** | 7 | **Study ID** | Badner1992 | **Assessor** | Ali Ashraf & Salem Elshenawy |
| **Ref or Label** |  | **Aim** | assignment to intervention (the 'intention-to-treat' effect) |  |  |
| **Experimental** | pulsatile | **Comparator** | Non-pulsatile | **Source** | Journal article(s) |
| **Outcome** | The renal effects of pulsatile (pulse pressure 18.0 ± 1.5 mm Hg [mean * SEMI) or nonpulsatile perfusion(mean pulse pressure 1.9 ± 0.4 mm Hg) during either a-stat (mean Paco, 41.2 ± 0.9 mm Hg measured at 37°C) or pH-stat (mean Paco, 60.6 ± 1.7 mm Hg measured at 37°C) pH management of hypothermic cardiopulmonary bypass (CPB) were studied in 100 patients undergoing elective coronary artery bypass surgery. Mean urine output, fractional excretion of sodium and potassium, and renal failure index all increased during the study period; however, there was no difference among the four different CPB management groups. Mean postoperative creatinine and blood urea nitrogen values decreased compared with preoperative values | **Results** | The renal effects of pulsatile (pulse pressure 18.0 ± 1.5 mm Hg [mean * SEMI) or nonpulsatile perfusion(mean pulse pressure 1.9 ± 0.4 mm Hg) during either a-stat (mean Paco, 41.2 ± 0.9 mm Hg measured at 37°C) or pH-stat (mean Paco, 60.6 ± 1.7 mm Hg measured at 37°C) pH management of hypothermic cardiopulmonary bypass (CPB) were studied in 100 patients undergoing elective coronary artery bypass surgery. Mean urine output, fractional excretion of sodium and potassium, and renal failure index all increased during the study period; however, there was no difference among the four different CPB management groups. Mean postoperative creatinine and blood urea nitrogen values decreased compared with preoperative values | **Weight** | 1 |
| **Domain** | **Signalling question** | | | **Response** | **Comments** |
| **Bias arising from the randomization process** | 1.1 Was the allocation sequence random? | | | Y | The study was begun after institutional approval and  written, informed consent were obtained. As part of  an ongoing study evaluating neuropsychologic outcomes, patients undergoing elective CAB surgery  were randomly assigned to one of four treatment  groups to receive either pulsatile or nonpulsatile  perfusion and either pH-stat or a-stat pH martagement during hypothermic CPB. This randomization  was stratified according to surgeon (n = 4), to account  for differences in technique. |
|  | 1.2 Was the allocation sequence concealed until participants were enrolled and assigned to interventions? | | | NI |  |
|  | 1.3 Did baseline differences between intervention groups suggest a problem with the randomization process? | | | N |  |
|  | **Risk of bias judgement** | | | **Some concerns** |  |
| **Bias due to deviations from intended interventions** | 2.1.Were participants aware of their assigned intervention during the trial? | | | NI | The study was begun after institutional approval and written, informed consent were obtained. As part of an ongoing study evaluating neuropsychologic outcomes, patients undergoing elective CAB surgery  were randomly assigned to one of four treatment groups to receive either pulsatile or nonpulsatile perfusion and either pH-stat or a-stat pH martagement during hypothermic CPB. This randomization was stratified according to surgeon (n = 4), to account for differences in technique. |
|  | 2.2.Were carers and people delivering the interventions aware of participants' assigned intervention during the trial? | | | Y |  |
|  | 2.3. If Y/PY/NI to 2.1 or 2.2: Were there deviations from the intended intervention that arose because of the experimental context? | | | N |  |
|  | 2.4 If Y/PY to 2.3: Were these deviations likely to have affected the outcome? | | | NA |  |
|  | 2.5. If Y/PY/NI to 2.4: Were these deviations from intended intervention balanced between groups? | | | NA |  |
|  | 2.6 Was an appropriate analysis used to estimate the effect of assignment to intervention? | | | PY | Demographic data were compared using one-way analysis of variance with 2 analysis for comparison of gender distribution. Intraoperative variables were analyzed with one-way analysis of variance, whereas measurements of renal function were analyzed using repeated measures analysis of variance with StudentNewman-Keuls post hoc testing when differences were found. A P value <0.05 was considered statistically significant |
|  | 2.7 If N/PN/NI to 2.6: Was there potential for a substantial impact (on the result) of the failure to analyse participants in the group to which they were randomized? | | | NA |  |
|  | **Risk of bias judgement** | | | **Low** |  |
| **Bias due to missing outcome data** | 3.1 Were data for this outcome available for all, or nearly all, participants randomized? | | | PY | One hundred six patients undergoing elective CAB  surgery were enrolled in the study. Six patients  received diuretics intraoperatively and were excluded  from data analysis, leaving 100 patients. Their demographic data are listed in Table 1. There were no  statistically significant differences among the four  groups with respect to patient age, height, weight,  gender distribution, number of vessels grafted, or  duration of CPB. There was also no difference in the  intraoperative use of dopamine among the four groups |
|  | 3.2 If N/PN/NI to 3.1: Is there evidence that result was not biased by missing outcome data? | | | NA |  |
|  | 3.3 If N/PN to 3.2: Could missingness in the outcome depend on its true value? | | | NA |  |
|  | 3.4 If Y/PY/NI to 3.3: Is it likely that missingness in the outcome depended on its true value? | | | NA |  |
|  | **Risk of bias judgement** | | | **Low** |  |
| **Bias in measurement of the outcome** | 4.1 Was the method of measuring the outcome inappropriate? | | | N | The renal effects of pulsatile (pulse pressure 18.0 ± 1.5 mm Hg [mean * SEMI) or nonpulsatile perfusion(mean pulse pressure 1.9 ± 0.4 mm Hg) during either a-stat (mean Paco, 41.2 ± 0.9 mm Hg measured at 37°C) or pH-stat (mean Paco, 60.6 ± 1.7 mm Hg measured at 37°C) pH management of hypothermic cardiopulmonary bypass (CPB) were studied in 100 patients undergoing elective coronary artery bypass surgery. Mean urine output, fractional excretion of sodium and potassium, and renal failure index all increased during the study period; however, there was no difference among the four different CPB management groups. Mean postoperative creatinine and blood urea nitrogen values decreased compared with preoperative values |
|  | 4.2 Could measurement or ascertainment of the outcome have differed between intervention groups? | | | N |  |
|  | 4.3 Were outcome assessors aware of the intervention received by study participants? | | | NI |  |
|  | 4.4 If Y/PY/NI to 4.3: Could assessment of the outcome have been influenced by knowledge of intervention received? | | | N |  |
|  | 4.5 If Y/PY/NI to 4.4: Is it likely that assessment of the outcome was influenced by knowledge of intervention received? | | | NA |  |
|  | **Risk of bias judgement** | | | **Low** |  |
| **Bias in selection of the reported result** | 5.1 Were the data that produced this result analysed in accordance with a pre-specified analysis plan that was finalized before unblinded outcome data were available for analysis? | | | NI |  |
|  | 5.2 ... multiple eligible outcome measurements (e.g. scales, definitions, time points) within the outcome domain? | | | N |  |
|  | 5.3 ... multiple eligible analyses of the data? | | | N |  |
|  | **Risk of bias judgement** | | | **Some concerns** |  |
| **Overall bias** | **Risk of bias judgement** | | | **Some concerns** |  |

| **Unique ID** | Borulu et al. 2020 | | **Study ID** | | Borulu et al. 2020 | | |  | Ahmed Mazen Amin & Mohamed Ahmed Ali |  |  |
| --- | --- | --- | --- | --- | --- | --- | --- | --- | --- | --- | --- |
| **Ref or Label** | Borulu et al. 2020 | | **Aim** | | assignment to intervention (the 'intention-to-treat' effect) | | |  |  |  |  |
| **Domain** | **Signalling question** | | | | | | | **Response** | **Comments** |  |  |
| **Bias arising from the randomization process** | 1.1 Was the allocation sequence random? | | | | | | | Y | Patients were randomized into two groups by flipping a coin. |  |  |
|  | 1.2 Was the allocation sequence concealed until participants were enrolled and assigned to interventions? | | | | | | | NI |  |  |  |
|  | 1.3 Did baseline differences between intervention groups suggest a problem with the randomization process? | | | | | | | PN | No significant imbalances. |  |  |
|  | **Risk of bias judgement** | | | | | | | **Some concerns** |  |  |  |
| **Bias due to deviations from intended interventions** | 2.1. Were participants aware of their assigned intervention during the trial? | | | | | | | NI |  |  |  |
|  | 2.2. Were carers and people delivering the interventions aware of participants' assigned intervention during the trial? | | | | | | | NI |  |  |  |
|  | 2.3. If Y/PY/NI to 2.1 or 2.2: Were there deviations from the intended intervention that arose because of the experimental context? | | | | | | | PN | No apparent deviations were reported. |  |  |
|  | 2.4 If Y/PY to 2.3: Were these deviations likely to have affected the outcome? | | | | | | | NA |  |  |  |
|  | 2.5. If Y/PY/NI to 2.4: Were these deviations from the intended intervention balanced between groups? | | | | | | | NA |  |  |  |
|  | 2.6 Was an appropriate analysis used to estimate the effect of assignment to intervention? | | | | | | | N | No appropriate analysis was used. |  |  |
|  | 2.7 If N/PN/NI to 2.6: Was there potential for a substantial impact (on the result) of the failure to analyze participants in the group to which they were randomized? | | | | | | | PN |  |  |  |
|  | **Risk of bias judgement** | | | | | | | **Some concerns** |  |  |  |
| **Bias due to missing outcome data** | 3.1 Were data for this outcome available for all, or nearly all, participants randomized? | | | | | | | PY | The data for this outcome was available for nearly all participants who were randomized. |  |  |
|  | 3.2 If N/PN/NI to 3.1: Is there evidence that result was not biased by missing outcome data? | | | | | | | NA |  |  |  |
|  | 3.3 If N/PN to 3.2: Could missingness in the outcome depend on its true value? | | | | | | | NA |  |  |  |
|  | 3.4 If Y/PY/NI to 3.3: Is it likely that missingness in the outcome depended on its true value? | | | | | | | NA |  |  |  |
|  | **Risk of bias judgement** | | | | | | | **Low** |  |  |  |
| **Bias in measurement of the outcome** | 4.1 Was the method of measuring the outcome inappropriate? | | | | | | | PN | The methods used for measuring the outcome were appropriate. |  |  |
|  | 4.2 Could measurement or ascertainment of the outcome have differed between intervention groups? | | | | | | | PN | No difference in measurement was detected between the groups. |  |  |
|  | 4.3 Were outcome assessors aware of the intervention received by study participants? | | | | | | | NI |  |  |  |
|  | 4.4 If Y/PY/NI to 4.3: Could assessment of the outcome have been influenced by knowledge of intervention received? | | | | | | | PN |  |  |  |
|  | 4.5 If Y/PY/NI to 4.4: Is it likely that assessment of the outcome was influenced by knowledge of intervention received? | | | | | | | NA |  |  |  |
|  | **Risk of bias judgement** | | | | | | | **Low** |  |  |  |
| **Bias in selection of the reported result** | 5.1 Were the data that produced this result analysed in accordance with a pre-specified analysis plan that was finalized before unblinded outcome data were available for analysis? | | | | | | | NI |  |  |  |
|  | 5.2 ... multiple eligible outcome measurements (e.g. scales, definitions, time points) within the outcome domain? | | | | | | | PN | No multiple outcomes measurements. |  |  |
|  | 5.3 ... multiple eligible analyses of the data? | | | | | | | PN | No multiple analyses. |  |  |
|  | **Risk of bias judgement** | | | | | | | **Some concerns** |  |  |  |
| **Overall bias** | **Risk of bias judgement** | | | | | | | **Some concerns** |  |  |  |
|  |  | |  | |  | | |  |  |  |  |
|  |  | |  | |  | | |  |  |  |  |
| **Unique ID** | Dodonov et al. 2021 | | **Study ID** | | Dodonov et al. 2021 | | |  |  |  |  |
| **Ref or Label** | Dodonov et al. 2021 | | **Aim** | | assignment to intervention (the 'intention-to-treat' effect) | | |  |  |  |  |
| **Domain** | **Signalling question** | | | | | | | **Response** | **Comments** |  |  |
| **Bias arising from the randomization process** | 1.1 Was the allocation sequence random? | | | | | | | Y | All patients signed an informed consent, and the study protocol was approved by the University Ethical Committee (UEC). All patients were randomized by the Research Randomizer Program (http://www.randomizer.org; last accessed date: 22/02/2010) and divided into two perfusion-type groups. |  |  |
|  | 1.2 Was the allocation sequence concealed until participants were enrolled and assigned to interventions? | | | | | | | Y |  |  |  |
|  | 1.3 Did baseline differences between intervention groups suggest a problem with the randomization process? | | | | | | | N | There are no significant imbalances |  |  |
|  | **Risk of bias judgement** | | | | | | | **Low** |  |  |  |
| **Bias due to deviations from intended interventions** | 2.1. Were participants aware of their assigned intervention during the trial? | | | | | | | NI |  |  |  |
|  | 2.2. Were carers and people delivering the interventions aware of participants' assigned intervention during the trial? | | | | | | | NI |  |  |  |
|  | 2.3. If Y/PY/NI to 2.1 or 2.2: Were there deviations from the intended intervention that arose because of the experimental context? | | | | | | | PN | No apparent deviations were reported. |  |  |
|  | 2.4 If Y/PY to 2.3: Were these deviations likely to have affected the outcome? | | | | | | | NA |  |  |  |
|  | 2.5. If Y/PY/NI to 2.4: Were these deviations from the intended intervention balanced between groups? | | | | | | | NA |  |  |  |
|  | 2.6 Was an appropriate analysis used to estimate the effect of assignment to intervention? | | | | | | | PN | No appropriate analysis was used. |  |  |
|  | 2.7 If N/PN/NI to 2.6: Was there potential for a substantial impact (on the result) of the failure to analyse participants in the group to which they were randomized? | | | | | | | PN |  |  |  |
|  | **Risk of bias judgement** | | | | | | | **Some concerns** |  |  |  |
| **Bias due to missing outcome data** | 3.1 Were data for this outcome available for all, or nearly all, participants randomized? | | | | | | | PY | The data for this outcome was available for nearly all participants who were randomized. |  |  |
|  | 3.2 If N/PN/NI to 3.1: Is there evidence that result was not biased by missing outcome data? | | | | | | | NA |  |  |  |
|  | 3.3 If N/PN to 3.2: Could missingness in the outcome depend on its true value? | | | | | | | NA |  |  |  |
|  | 3.4 If Y/PY/NI to 3.3: Is it likely that missingness in the outcome depended on its true value? | | | | | | | NA |  |  |  |
|  | **Risk of bias judgement** | | | | | | | **Low** |  |  |  |
| **Bias in measurement of the outcome** | 4.1 Was the method of measuring the outcome inappropriate? | | | | | | | PN | The methods used for measuring the outcome were appropriate. |  |  |
|  | 4.2 Could measurement or ascertainment of the outcome have differed between intervention groups? | | | | | | | PN | No difference in measurement was detected between the groups. |  |  |
|  | 4.3 Were outcome assessors aware of the intervention received by study participants? | | | | | | | NI |  |  |  |
|  | 4.4 If Y/PY/NI to 4.3: Could assessment of the outcome have been influenced by knowledge of intervention received? | | | | | | | PN |  |  |  |
|  | 4.5 If Y/PY/NI to 4.4: Is it likely that assessment of the outcome was influenced by knowledge of intervention received? | | | | | | | NA |  |  |  |
|  | **Risk of bias judgement** | | | | | | | **Low** |  |  |  |
| **Bias in selection of the reported result** | 5.1 Were the data that produced this result analysed in accordance with a pre-specified analysis plan that was finalized before unblinded outcome data were available for analysis? | | | | | | | NI |  |  |  |
|  | 5.2 ... multiple eligible outcome measurements (e.g. scales, definitions, time points) within the outcome domain? | | | | | | | PN | No multiple outcomes measurements. |  |  |
|  | 5.3 ... multiple eligible analyses of the data? | | | | | | | PN | No multiple analyses. |  |  |
|  | **Risk of bias judgement** | | | | | | | **Some concerns** |  |  |  |
| **Overall bias** | **Risk of bias judgement** | | | | | | | **Some concerns** |  |  |  |
|  |  | |  | |  | | |  |  |  |  |
|  |  | |  | |  | | |  |  |  |  |
| **Unique ID** | Driessen et al. 1995 | | **Study ID** | | Driessen et al. 1995 | | |  | Ahmed Mazen Amin & Mohamed Ahmed Ali |  |  |
| **Ref or Label** | Driessen et al. 1995 | | **Aim** | | assignment to intervention (the 'intention-to-treat' effect) | | |  |  |  |  |
| **Domain** | **Signalling question** | | | | | | | **Response** | **Comments** |  |  |
| **Bias arising from the randomization process** | 1.1 Was the allocation sequence random? | | | | | | | NI | Patients were randomly divided into two groups of 19 patients. |  |  |
|  | 1.2 Was the allocation sequence concealed until participants were enrolled and assigned to interventions? | | | | | | | NI |  |  |  |
|  | 1.3 Did baseline differences between intervention groups suggest a problem with the randomization process? | | | | | | | PN | There are no significant imbalances |  |  |
|  | **Risk of bias judgement** | | | | | | | **Some concerns** |  |  |  |
| **Bias due to deviations from intended interventions** | 2.1.Were participants aware of their assigned intervention during the trial? | | | | | | | NI |  |  |  |
|  | 2.2.Were carers and people delivering the interventions aware of participants' assigned intervention during the trial? | | | | | | | NI |  |  |  |
|  | 2.3. If Y/PY/NI to 2.1 or 2.2: Were there deviations from the intended intervention that arose because of the experimental context? | | | | | | | PN | No apparent deviations were reported. |  |  |
|  | 2.4 If Y/PY to 2.3: Were these deviations likely to have affected the outcome? | | | | | | | NA |  |  |  |
|  | 2.5. If Y/PY/NI to 2.4: Were these deviations from intended intervention balanced between groups? | | | | | | | NA |  |  |  |
|  | 2.6 Was an appropriate analysis used to estimate the effect of assignment to intervention? | | | | | | | PN | No appropriate analysis was used. |  |  |
|  | 2.7 If N/PN/NI to 2.6: Was there potential for a substantial impact (on the result) of the failure to analyse participants in the group to which they were randomized? | | | | | | | PN |  |  |  |
|  | **Risk of bias judgement** | | | | | | | **Some concerns** |  |  |  |
| **Bias due to missing outcome data** | 3.1 Were data for this outcome available for all, or nearly all, participants randomized? | | | | | | | PY | The data for this outcome was available for nearly all participants who were randomized. |  |  |
|  | 3.2 If N/PN/NI to 3.1: Is there evidence that result was not biased by missing outcome data? | | | | | | | NA |  |  |  |
|  | 3.3 If N/PN to 3.2: Could missingness in the outcome depend on its true value? | | | | | | | NA |  |  |  |
|  | 3.4 If Y/PY/NI to 3.3: Is it likely that missingness in the outcome depended on its true value? | | | | | | | NA |  |  |  |
|  | **Risk of bias judgement** | | | | | | | **Low** |  |  |  |
| **Bias in measurement of the outcome** | 4.1 Was the method of measuring the outcome inappropriate? | | | | | | | PN | The methods used for measuring the outcome were appropriate. |  |  |
|  | 4.2 Could measurement or ascertainment of the outcome have differed between intervention groups? | | | | | | | PN | No difference in measurement was detected between the groups. |  |  |
|  | 4.3 Were outcome assessors aware of the intervention received by study participants? | | | | | | | NI |  |  |  |
|  | 4.4 If Y/PY/NI to 4.3: Could assessment of the outcome have been influenced by knowledge of intervention received? | | | | | | | PN |  |  |  |
|  | 4.5 If Y/PY/NI to 4.4: Is it likely that assessment of the outcome was influenced by knowledge of intervention received? | | | | | | | NA |  |  |  |
|  | **Risk of bias judgement** | | | | | | | **Low** |  |  |  |
| **Bias in selection of the reported result** | 5.1 Were the data that produced this result analysed in accordance with a pre-specified analysis plan that was finalized before unblinded outcome data were available for analysis? | | | | | | | NI |  |  |  |
|  | 5.2 ... multiple eligible outcome measurements (e.g. scales, definitions, time points) within the outcome domain? | | | | | | | PN | No multiple outcomes measurements. |  |  |
|  | 5.3 ... multiple eligible analyses of the data? | | | | | | | PN | No multiple analyses. |  |  |
|  | **Risk of bias judgement** | | | | | | | **Some concerns** |  |  |  |
| **Overall bias** | **Risk of bias judgement** | | | | | | | **Some concerns** |  |  |  |
|  |  | |  | |  | | |  |  |  |  |
|  |  | |  | |  | | |  |  |  |  |
| **Unique ID** | Engels et al. 2014 | | **Study ID** | | Engels et al. 2014 | | |  | Ahmed Mazen Amin & Mohamed Ahmed Ali |  |  |
| **Ref or Label** | Engels et al. 2014 | | **Aim** | | assignment to intervention (the 'intention-to-treat' effect) | | |  |  |  |  |
| **Domain** | **Signalling question** | | | | | | | **Response** | **Comments** |  |  |
| **Bias arising from the randomization process** | 1.1 Was the allocation sequence random? | | | | | | | NI | The topic of pulsatile flow is one with much controversy. In this study, 37 patients were randomly assigned to either perfusion with the centrifugal pump in pulsatile mode or in continuous mode. |  |  |
|  | 1.2 Was the allocation sequence concealed until participants were enrolled and assigned to interventions? | | | | | | | NI |  |  |  |
|  | 1.3 Did baseline differences between intervention groups suggest a problem with the randomization process? | | | | | | | PN | There are no significant imbalances |  |  |
|  | **Risk of bias judgement** | | | | | | | **Some concerns** |  |  |  |
| **Bias due to deviations from intended interventions** | 2.1.Were participants aware of their assigned intervention during the trial? | | | | | | | NI |  |  |  |
|  | 2.2.Were carers and people delivering the interventions aware of participants' assigned intervention during the trial? | | | | | | | NI |  |  |  |
|  | 2.3. If Y/PY/NI to 2.1 or 2.2: Were there deviations from the intended intervention that arose because of the experimental context? | | | | | | | PN | No apparent deviations were reported. |  |  |
|  | 2.4 If Y/PY to 2.3: Were these deviations likely to have affected the outcome? | | | | | | | NA |  |  |  |
|  | 2.5. If Y/PY/NI to 2.4: Were these deviations from intended intervention balanced between groups? | | | | | | | NA |  |  |  |
|  | 2.6 Was an appropriate analysis used to estimate the effect of assignment to intervention? | | | | | | | PN | No appropriate analysis was used. |  |  |
|  | 2.7 If N/PN/NI to 2.6: Was there potential for a substantial impact (on the result) of the failure to analyse participants in the group to which they were randomized? | | | | | | | PN |  |  |  |
|  | **Risk of bias judgement** | | | | | | | **Some concerns** |  |  |  |
| **Bias due to missing outcome data** | 3.1 Were data for this outcome available for all, or nearly all, participants randomized? | | | | | | | PY | The data for this outcome was available for nearly all participants who were randomized. |  |  |
|  | 3.2 If N/PN/NI to 3.1: Is there evidence that result was not biased by missing outcome data? | | | | | | | NA |  |  |  |
|  | 3.3 If N/PN to 3.2: Could missingness in the outcome depend on its true value? | | | | | | | NA |  |  |  |
|  | 3.4 If Y/PY/NI to 3.3: Is it likely that missingness in the outcome depended on its true value? | | | | | | | NA |  |  |  |
|  | **Risk of bias judgement** | | | | | | | **Low** |  |  |  |
| **Bias in measurement of the outcome** | 4.1 Was the method of measuring the outcome inappropriate? | | | | | | | PN | The methods used for measuring the outcome were appropriate. |  |  |
|  | 4.2 Could measurement or ascertainment of the outcome have differed between intervention groups? | | | | | | | PN | No difference in measurement was detected between the groups. |  |  |
|  | 4.3 Were outcome assessors aware of the intervention received by study participants? | | | | | | | NI |  |  |  |
|  | 4.4 If Y/PY/NI to 4.3: Could assessment of the outcome have been influenced by knowledge of intervention received? | | | | | | | PN |  |  |  |
|  | 4.5 If Y/PY/NI to 4.4: Is it likely that assessment of the outcome was influenced by knowledge of intervention received? | | | | | | | NA |  |  |  |
|  | **Risk of bias judgement** | | | | | | | **Low** |  |  |  |
| **Bias in selection of the reported result** | 5.1 Were the data that produced this result analysed in accordance with a pre-specified analysis plan that was finalized before unblinded outcome data were available for analysis? | | | | | | | NI |  |  |  |
|  | 5.2 ... multiple eligible outcome measurements (e.g. scales, definitions, time points) within the outcome domain? | | | | | | | PN | No multiple outcomes measurements. |  |  |
|  | 5.3 ... multiple eligible analyses of the data? | | | | | | | PN | No multiple analyses. |  |  |
|  | **Risk of bias judgement** | | | | | | | **Some concerns** |  |  |  |
| **Overall bias** | **Risk of bias judgement** | | | | | | | **Some concerns** |  |  |  |
|  |  | |  | |  | | |  |  |  |  |
|  |  | |  | |  | | |  |  |  |  |
| **Unique ID** | Graßler et al. 2019 | | **Study ID** | | Graßler et al. 2019 | | | **Assessor** | Ahmed Mazen Amin & Mohamed Ahmed Ali |  |  |
| **Ref or Label** | Graßler et al. 2019 | | **Aim** | | assignment to intervention (the 'intention-to-treat' effect) | | |  |  |  |  |
| **Domain** | **Signalling question** | | | | | | | **Response** | **Comments** |  |  |
| **Bias arising from the randomization process** | 1.1 Was the allocation sequence random? | | | | | | | NI | Patients were randomly allocated to a pulsatile perfusion group (n = 16) or a nonpulsatile perfusion group (n = 16)." |  |  |
|  | 1.2 Was the allocation sequence concealed until participants were enrolled and assigned to interventions? | | | | | | | NI |  |  |  |
|  | 1.3 Did baseline differences between intervention groups suggest a problem with the randomization process? | | | | | | | PN | There are no significant imbalances |  |  |
|  | **Risk of bias judgement** | | | | | | | **Some concerns** |  |  |  |
| **Bias due to deviations from intended interventions** | 2.1.Were participants aware of their assigned intervention during the trial? | | | | | | | NI |  |  |  |
|  | 2.2.Were carers and people delivering the interventions aware of participants' assigned intervention during the trial? | | | | | | | NI |  |  |  |
|  | 2.3. If Y/PY/NI to 2.1 or 2.2: Were there deviations from the intended intervention that arose because of the experimental context? | | | | | | | PN | No apparent deviations were reported. |  |  |
|  | 2.4 If Y/PY to 2.3: Were these deviations likely to have affected the outcome? | | | | | | | NA |  |  |  |
|  | 2.5. If Y/PY/NI to 2.4: Were these deviations from intended intervention balanced between groups? | | | | | | | NA |  |  |  |
|  | 2.6 Was an appropriate analysis used to estimate the effect of assignment to intervention? | | | | | | | PN | No appropriate analysis was used. |  |  |
|  | 2.7 If N/PN/NI to 2.6: Was there potential for a substantial impact (on the result) of the failure to analyse participants in the group to which they were randomized? | | | | | | | PN |  |  |  |
|  | **Risk of bias judgement** | | | | | | | **Some concerns** |  |  |  |
| **Bias due to missing outcome data** | 3.1 Were data for this outcome available for all, or nearly all, participants randomized? | | | | | | | PY | The data for this outcome was available for nearly all participants who were randomized. |  |  |
|  | 3.2 If N/PN/NI to 3.1: Is there evidence that result was not biased by missing outcome data? | | | | | | | NA |  |  |  |
|  | 3.3 If N/PN to 3.2: Could missingness in the outcome depend on its true value? | | | | | | | NA |  |  |  |
|  | 3.4 If Y/PY/NI to 3.3: Is it likely that missingness in the outcome depended on its true value? | | | | | | | NA |  |  |  |
|  | **Risk of bias judgement** | | | | | | | **Low** |  |  |  |
| **Bias in measurement of the outcome** | 4.1 Was the method of measuring the outcome inappropriate? | | | | | | | PN | The methods used for measuring the outcome were appropriate. |  |  |
|  | 4.2 Could measurement or ascertainment of the outcome have differed between intervention groups? | | | | | | | PN | No difference in measurement was detected between the groups. |  |  |
|  | 4.3 Were outcome assessors aware of the intervention received by study participants? | | | | | | | NI |  |  |  |
|  | 4.4 If Y/PY/NI to 4.3: Could assessment of the outcome have been influenced by knowledge of intervention received? | | | | | | | PN |  |  |  |
|  | 4.5 If Y/PY/NI to 4.4: Is it likely that assessment of the outcome was influenced by knowledge of intervention received? | | | | | | | NA |  |  |  |
|  | **Risk of bias judgement** | | | | | | | **Low** |  |  |  |
| **Bias in selection of the reported result** | 5.1 Were the data that produced this result analysed in accordance with a pre-specified analysis plan that was finalized before unblinded outcome data were available for analysis? | | | | | | | NI |  |  |  |
|  | 5.2 ... multiple eligible outcome measurements (e.g. scales, definitions, time points) within the outcome domain? | | | | | | | PN | No multiple outcomes measurements. |  |  |
|  | 5.3 ... multiple eligible analyses of the data? | | | | | | | PN | No multiple analyses. |  |  |
|  | **Risk of bias judgement** | | | | | | | **Some concerns** |  |  |  |
| **Overall bias** | **Risk of bias judgement** | | | | | | | **Some concerns** |  |  |  |
|  |  | |  | |  | | |  |  |  |  |
|  |  | |  | |  | | |  |  |  |  |
| **Unique ID** | Gu et al. 2011 | | **Study ID** | | Gu et al. 2011 | | | **Assessor** | Ahmed Mazen Amin & Mohamed Ahmed Ali |  |  |
| **Ref or Label** | Gu et al. 2011 | | **Aim** | | assignment to intervention (the 'intention-to-treat' effect) | | |  |  |  |  |
| **Domain** | **Signalling question** | | | | | | | **Response** | **Comments** |  |  |
| **Bias arising from the randomization process** | 1.1 Was the allocation sequence random? | | | | | | | Y | Treatments were assigned in a block-random design to achieve similar group sizes. Random sequences were generated in the Institute of Clinical Epidemiology and Biostatistics, University of Ulm, Germany, using the software R (19). |  |  |
|  | 1.2 Was the allocation sequence concealed until participants were enrolled and assigned to interventions? | | | | | | | Y |  |  |  |
|  | 1.3 Did baseline differences between intervention groups suggest a problem with the randomization process? | | | | | | | N | There are no significant imbalances |  |  |
|  | **Risk of bias judgement** | | | | | | | **Low** |  |  |  |
| **Bias due to deviations from intended interventions** | 2.1.Were participants aware of their assigned intervention during the trial? | | | | | | | NI |  |  |  |
|  | 2.2.Were carers and people delivering the interventions aware of participants' assigned intervention during the trial? | | | | | | | NI |  |  |  |
|  | 2.3. If Y/PY/NI to 2.1 or 2.2: Were there deviations from the intended intervention that arose because of the experimental context? | | | | | | | PN | No apparent deviations were reported. |  |  |
|  | 2.4 If Y/PY to 2.3: Were these deviations likely to have affected the outcome? | | | | | | | NA |  |  |  |
|  | 2.5. If Y/PY/NI to 2.4: Were these deviations from intended intervention balanced between groups? | | | | | | | NA |  |  |  |
|  | 2.6 Was an appropriate analysis used to estimate the effect of assignment to intervention? | | | | | | | N | No appropriate analysis was used. |  |  |
|  | 2.7 If N/PN/NI to 2.6: Was there potential for a substantial impact (on the result) of the failure to analyse participants in the group to which they were randomized? | | | | | | | PN |  |  |  |
|  | **Risk of bias judgement** | | | | | | | **Some concerns** |  |  |  |
| **Bias due to missing outcome data** | 3.1 Were data for this outcome available for all, or nearly all, participants randomized? | | | | | | | PY | The data for this outcome was available for nearly all participants who were randomized. |  |  |
|  | 3.2 If N/PN/NI to 3.1: Is there evidence that result was not biased by missing outcome data? | | | | | | | NA |  |  |  |
|  | 3.3 If N/PN to 3.2: Could missingness in the outcome depend on its true value? | | | | | | | NA |  |  |  |
|  | 3.4 If Y/PY/NI to 3.3: Is it likely that missingness in the outcome depended on its true value? | | | | | | | NA |  |  |  |
|  | **Risk of bias judgement** | | | | | | | **Low** |  |  |  |
| **Bias in measurement of the outcome** | 4.1 Was the method of measuring the outcome inappropriate? | | | | | | | PN | The methods used for measuring the outcome were appropriate. |  |  |
|  | 4.2 Could measurement or ascertainment of the outcome have differed between intervention groups? | | | | | | | PN | No difference in measurement was detected between the groups. |  |  |
|  | 4.3 Were outcome assessors aware of the intervention received by study participants? | | | | | | | NI |  |  |  |
|  | 4.4 If Y/PY/NI to 4.3: Could assessment of the outcome have been influenced by knowledge of intervention received? | | | | | | | PN |  |  |  |
|  | 4.5 If Y/PY/NI to 4.4: Is it likely that assessment of the outcome was influenced by knowledge of intervention received? | | | | | | | NA |  |  |  |
|  | **Risk of bias judgement** | | | | | | | **Low** |  |  |  |
| **Bias in selection of the reported result** | 5.1 Were the data that produced this result analysed in accordance with a pre-specified analysis plan that was finalized before unblinded outcome data were available for analysis? | | | | | | | NI |  |  |  |
|  | 5.2 ... multiple eligible outcome measurements (e.g. scales, definitions, time points) within the outcome domain? | | | | | | | PN | No multiple outcomes measurements. |  |  |
|  | 5.3 ... multiple eligible analyses of the data? | | | | | | | PN | No multiple analyses. |  |  |
|  | **Risk of bias judgement** | | | | | | | **Some concerns** |  |  |  |
| **Overall bias** | **Risk of bias judgement** | | | | | | | **Some concerns** |  |  |  |
| **Unique ID** | Jiang et al. 2021 | | | **Study ID** | | Jiang et al. 2021 | | |  | Abdallah Saeed & Majd M. AlBarakat |  |
| **Ref or Label** | Jiang et al. 2021 | | | **Aim** | | assignment to intervention (the 'intention-to-treat' effect) | | |  |  |  |
| **Domain** | **Signalling question** | | | | | | | | **Response** | **Comments** |  |
| **Bias arising from the randomization process** | 1.1 Was the allocation sequence random? | | | | | | | | Y |  |  |
|  | 1.2 Was the allocation sequence concealed until participants were enrolled and assigned to interventions? | | | | | | | | Y |  |  |
|  | 1.3 Did baseline differences between intervention groups suggest a problem with the randomization process? | | | | | | | | N | There are no significant imbalances |  |
|  | **Risk of bias judgement** | | | | | | | | **Low** |  |  |
| **Bias due to deviations from intended interventions** | 2.1. Were participants aware of their assigned intervention during the trial? | | | | | | | | NI |  |  |
|  | 2.2. Were carers and people delivering the interventions aware of participants' assigned intervention during the trial? | | | | | | | | PY |  |  |
|  | 2.3. If Y/PY/NI to 2.1 or 2.2: Were there deviations from the intended intervention that arose because of the experimental context? | | | | | | | | NI |  |  |
|  | 2.4 If Y/PY to 2.3: Were these deviations likely to have affected the outcome? | | | | | | | | NA |  |  |
|  | 2.5. If Y/PY/NI to 2.4: Were these deviations from the intended intervention balanced between groups? | | | | | | | | NA |  |  |
|  | 2.6 Was an appropriate analysis used to estimate the effect of assignment to intervention? | | | | | | | | PY | Appropriate analysis was used. |  |
|  | 2.7 If N/PN/NI to 2.6: Was there potential for a substantial impact (on the result) of the failure to analyze participants in the group to which they were randomized? | | | | | | | | NA |  |  |
|  | **Risk of bias judgement** | | | | | | | | **Some concerns** |  |  |
| **Bias due to missing outcome data** | 3.1 Were data for this outcome available for all, or nearly all, participants randomized? | | | | | | | | PY | The data for this outcome was available for nearly all participants who were randomized. |  |
|  | 3.2 If N/PN/NI to 3.1: Is there evidence that result was not biased by missing outcome data? | | | | | | | | NA |  |  |
|  | 3.3 If N/PN to 3.2: Could missingness in the outcome depend on its true value? | | | | | | | | NA |  |  |
|  | 3.4 If Y/PY/NI to 3.3: Is it likely that missingness in the outcome depended on its true value? | | | | | | | | NA |  |  |
|  | **Risk of bias judgement** | | | | | | | | **Low** |  |  |
| **Bias in measurement of the outcome** | 4.1 Was the method of measuring the outcome inappropriate? | | | | | | | | N | The methods used for measuring the outcome were appropriate. |  |
|  | 4.2 Could measurement or ascertainment of the outcome have differed between intervention groups? | | | | | | | | N | No difference in measurement was detected between the groups. |  |
|  | 4.3 Were outcome assessors aware of the intervention received by study participants? | | | | | | | | NI |  |  |
|  | 4.4 If Y/PY/NI to 4.3: Could assessment of the outcome have been influenced by knowledge of intervention received? | | | | | | | | N |  |  |
|  | 4.5 If Y/PY/NI to 4.4: Is it likely that assessment of the outcome was influenced by knowledge of intervention received? | | | | | | | | NA |  |  |
|  | **Risk of bias judgement** | | | | | | | | **Low** |  |  |
| **Bias in selection of the reported result** | 5.1 Were the data that produced this result analysed in accordance with a pre-specified analysis plan that was finalized before unblinded outcome data were available for analysis? | | | | | | | | NI |  |  |
|  | 5.2 ... multiple eligible outcome measurements (e.g. scales, definitions, time points) within the outcome domain? | | | | | | | | PN |  |  |
|  | 5.3 ... multiple eligible analyses of the data? | | | | | | | | PN |  |  |
|  | **Risk of bias judgement** | | | | | | | | **Some concerns** |  |  |
| **Overall bias** | **Risk of bias judgement** | | | | | | | | **Some concerns** |  |  |
|  |  | | |  | |  | | |  |  |  |
|  |  | | |  | |  | | |  |  |  |
| **Unique ID** | Kocakulak et al. 2005 | | | **Study ID** | | Kocakulak et al. 2005 | | |  | Abdallah Saeed& Majd M. AlBarakat |  |
| **Ref or Label** | Kocakulak et al. 2005 | | | **Aim** | | assignment to intervention (the 'intention-to-treat' effect) | | |  |  |  |
| **Domain** | **Signalling question** | | | | | | | | **Response** | **Comments** |  |
| **Bias arising from the randomization process** | 1.1 Was the allocation sequence random? | | | | | | | | NI |  |  |
|  | 1.2 Was the allocation sequence concealed until participants were enrolled and assigned to interventions? | | | | | | | | NI |  |  |
|  | 1.3 Did baseline differences between intervention groups suggest a problem with the randomization process? | | | | | | | | N | There are no significant imbalances |  |
|  | **Risk of bias judgement** | | | | | | | | **Some concerns** |  |  |
| **Bias due to deviations from intended interventions** | 2.1. Were participants aware of their assigned intervention during the trial? | | | | | | | | NI |  |  |
|  | 2.2. Were carers and people delivering the interventions aware of participants' assigned intervention during the trial? | | | | | | | | NI |  |  |
|  | 2.3. If Y/PY/NI to 2.1 or 2.2: Were there deviations from the intended intervention that arose because of the experimental context? | | | | | | | | NI |  |  |
|  | 2.4 If Y/PY to 2.3: Were these deviations likely to have affected the outcome? | | | | | | | | NA |  |  |
|  | 2.5. If Y/PY/NI to 2.4: Were these deviations from the intended intervention balanced between groups? | | | | | | | | NA |  |  |
|  | 2.6 Was an appropriate analysis used to estimate the effect of assignment to intervention? | | | | | | | | NI |  |  |
|  | 2.7 If N/PN/NI to 2.6: Was there potential for a substantial impact (on the result) of the failure to analyse participants in the group to which they were randomized? | | | | | | | | NI |  |  |
|  | **Risk of bias judgement** | | | | | | | | **High** |  |  |
| **Bias due to missing outcome data** | 3.1 Were data for this outcome available for all, or nearly all, participants randomized? | | | | | | | | PY | The data for this outcome was available for nearly all participants who were randomized. |  |
|  | 3.2 If N/PN/NI to 3.1: Is there evidence that result was not biased by missing outcome data? | | | | | | | | NA |  |  |
|  | 3.3 If N/PN to 3.2: Could missingness in the outcome depend on its true value? | | | | | | | | NA |  |  |
|  | 3.4 If Y/PY/NI to 3.3: Is it likely that missingness in the outcome depended on its true value? | | | | | | | | NA |  |  |
|  | **Risk of bias judgement** | | | | | | | | **Low** |  |  |
| **Bias in measurement of the outcome** | 4.1 Was the method of measuring the outcome inappropriate? | | | | | | | | PN | The methods used for measuring the outcome were appropriate. |  |
|  | 4.2 Could measurement or ascertainment of the outcome have differed between intervention groups? | | | | | | | | N | No difference in measurement was detected between the groups. |  |
|  | 4.3 Were outcome assessors aware of the intervention received by study participants? | | | | | | | | NI |  |  |
|  | 4.4 If Y/PY/NI to 4.3: Could assessment of the outcome have been influenced by knowledge of intervention received? | | | | | | | | N |  |  |
|  | 4.5 If Y/PY/NI to 4.4: Is it likely that assessment of the outcome was influenced by knowledge of intervention received? | | | | | | | | NA |  |  |
|  | **Risk of bias judgement** | | | | | | | | **Low** |  |  |
| **Bias in selection of the reported result** | 5.1 Were the data that produced this result analysed in accordance with a pre-specified analysis plan that was finalized before unblinded outcome data were available for analysis? | | | | | | | | NI |  |  |
|  | 5.2 ... multiple eligible outcome measurements (e.g. scales, definitions, time points) within the outcome domain? | | | | | | | | PN | No multiple outcomes measurements. |  |
|  | 5.3 ... multiple eligible analyses of the data? | | | | | | | | PN | No multiple analyses. |  |
|  | **Risk of bias judgement** | | | | | | | | **Some concerns** |  |  |
| **Overall bias** | **Risk of bias judgement** | | | | | | | | **Some concerns** |  |  |
|  |  | | |  | |  | | |  |  |  |
|  |  | | |  | |  | | |  |  |  |
| **Unique ID** | Louagie et al. 1992 | | | **Study ID** | | Louagie et al. 1992 | | |  | Abdallah Saeed& Majd M. AlBarakat |  |
| **Ref or Label** | Louagie et al. 1992 | | | **Aim** | | assignment to intervention (the 'intention-to-treat' effect) | | |  |  |  |
| **Domain** | **Signalling question** | | | | | | | | **Response** | **Comments** |  |
| **Bias arising from the randomization process** | 1.1 Was the allocation sequence random? | | | | | | | | PY |  |  |
|  | 1.2 Was the allocation sequence concealed until participants were enrolled and assigned to interventions? | | | | | | | | Y |  |  |
|  | 1.3 Did baseline differences between intervention groups suggest a problem with the randomization process? | | | | | | | | N | There are no significant imbalances |  |
|  | **Risk of bias judgement** | | | | | | | | **Low** |  |  |
| **Bias due to deviations from intended interventions** | 2.1.Were participants aware of their assigned intervention during the trial? | | | | | | | | NI |  |  |
|  | 2.2.Were carers and people delivering the interventions aware of participants' assigned intervention during the trial? | | | | | | | | PY |  |  |
|  | 2.3. If Y/PY/NI to 2.1 or 2.2: Were there deviations from the intended intervention that arose because of the experimental context? | | | | | | | | NI |  |  |
|  | 2.4 If Y/PY to 2.3: Were these deviations likely to have affected the outcome? | | | | | | | | NA |  |  |
|  | 2.5. If Y/PY/NI to 2.4: Were these deviations from intended intervention balanced between groups? | | | | | | | | NA |  |  |
|  | 2.6 Was an appropriate analysis used to estimate the effect of assignment to intervention? | | | | | | | | PY | Appropriate analysis was used. |  |
|  | 2.7 If N/PN/NI to 2.6: Was there potential for a substantial impact (on the result) of the failure to analyse participants in the group to which they were randomized? | | | | | | | | NA |  |  |
|  | **Risk of bias judgement** | | | | | | | | **Some concerns** |  |  |
| **Bias due to missing outcome data** | 3.1 Were data for this outcome available for all, or nearly all, participants randomized? | | | | | | | | PY | The data for this outcome was available for nearly all participants who were randomized. |  |
|  | 3.2 If N/PN/NI to 3.1: Is there evidence that result was not biased by missing outcome data? | | | | | | | | NA |  |  |
|  | 3.3 If N/PN to 3.2: Could missingness in the outcome depend on its true value? | | | | | | | | NA |  |  |
|  | 3.4 If Y/PY/NI to 3.3: Is it likely that missingness in the outcome depended on its true value? | | | | | | | | NA |  |  |
|  | **Risk of bias judgement** | | | | | | | | **Low** |  |  |
| **Bias in measurement of the outcome** | 4.1 Was the method of measuring the outcome inappropriate? | | | | | | | | PN | The methods used for measuring the outcome were appropriate. |  |
|  | 4.2 Could measurement or ascertainment of the outcome have differed between intervention groups? | | | | | | | | PN | No difference in measurement was detected between the groups. |  |
|  | 4.3 Were outcome assessors aware of the intervention received by study participants? | | | | | | | | NI |  |  |
|  | 4.4 If Y/PY/NI to 4.3: Could assessment of the outcome have been influenced by knowledge of intervention received? | | | | | | | | N |  |  |
|  | 4.5 If Y/PY/NI to 4.4: Is it likely that assessment of the outcome was influenced by knowledge of intervention received? | | | | | | | | NA |  |  |
|  | **Risk of bias judgement** | | | | | | | | **Low** |  |  |
| **Bias in selection of the reported result** | 5.1 Were the data that produced this result analysed in accordance with a pre-specified analysis plan that was finalized before unblinded outcome data were available for analysis? | | | | | | | | NI |  |  |
|  | 5.2 ... multiple eligible outcome measurements (e.g. scales, definitions, time points) within the outcome domain? | | | | | | | | PN | No multiple outcomes measurements. |  |
|  | 5.3 ... multiple eligible analyses of the data? | | | | | | | | PN | No multiple analyses. |  |
|  | **Risk of bias judgement** | | | | | | | | **Some concerns** |  |  |
| **Overall bias** | **Risk of bias judgement** | | | | | | | | **Some concerns** |  |  |
|  |  | | |  | |  | | |  |  |  |
|  |  | | |  | |  | | |  |  |  |
| **Unique ID** | Mali et al. 2021 | | | **Study ID** | | Mali et al. 2021 | | |  | Abdallah Saeed & Majd M. AlBarakat |  |
| **Ref or Label** | Mali et al. 2021 | | | **Aim** | | assignment to intervention (the 'intention-to-treat' effect) | | |  |  |  |
| **Domain** | **Signalling question** | | | | | | | | **Response** | **Comments** |  |
| **Bias arising from the randomization process** | 1.1 Was the allocation sequence random? | | | | | | | | PY |  |  |
|  | 1.2 Was the allocation sequence concealed until participants were enrolled and assigned to interventions? | | | | | | | | NI |  |  |
|  | 1.3 Did baseline differences between intervention groups suggest a problem with the randomization process? | | | | | | | | N | There are no significant imbalances |  |
|  | **Risk of bias judgement** | | | | | | | | **Some concerns** |  |  |
| **Bias due to deviations from intended interventions** | 2.1.Were participants aware of their assigned intervention during the trial? | | | | | | | | NI |  |  |
|  | 2.2.Were carers and people delivering the interventions aware of participants' assigned intervention during the trial? | | | | | | | | PY |  |  |
|  | 2.3. If Y/PY/NI to 2.1 or 2.2: Were there deviations from the intended intervention that arose because of the experimental context? | | | | | | | | NI |  |  |
|  | 2.4 If Y/PY to 2.3: Were these deviations likely to have affected the outcome? | | | | | | | | NA |  |  |
|  | 2.5. If Y/PY/NI to 2.4: Were these deviations from intended intervention balanced between groups? | | | | | | | | NA |  |  |
|  | 2.6 Was an appropriate analysis used to estimate the effect of assignment to intervention? | | | | | | | | PY |  |  |
|  | 2.7 If N/PN/NI to 2.6: Was there potential for a substantial impact (on the result) of the failure to analyse participants in the group to which they were randomized? | | | | | | | | NA |  |  |
|  | **Risk of bias judgement** | | | | | | | | **Some concerns** |  |  |
| **Bias due to missing outcome data** | 3.1 Were data for this outcome available for all, or nearly all, participants randomized? | | | | | | | | PY | The data for this outcome was available for nearly all participants who were randomized. |  |
|  | 3.2 If N/PN/NI to 3.1: Is there evidence that result was not biased by missing outcome data? | | | | | | | | NA |  |  |
|  | 3.3 If N/PN to 3.2: Could missingness in the outcome depend on its true value? | | | | | | | | NA |  |  |
|  | 3.4 If Y/PY/NI to 3.3: Is it likely that missingness in the outcome depended on its true value? | | | | | | | | NA |  |  |
|  | **Risk of bias judgement** | | | | | | | | **Low** |  |  |
| **Bias in measurement of the outcome** | 4.1 Was the method of measuring the outcome inappropriate? | | | | | | | | PN | The methods used for measuring the outcome were appropriate. |  |
|  | 4.2 Could measurement or ascertainment of the outcome have differed between intervention groups? | | | | | | | | N | No difference in measurement was detected between the groups. |  |
|  | 4.3 Were outcome assessors aware of the intervention received by study participants? | | | | | | | | NI |  |  |
|  | 4.4 If Y/PY/NI to 4.3: Could assessment of the outcome have been influenced by knowledge of intervention received? | | | | | | | | PN |  |  |
|  | 4.5 If Y/PY/NI to 4.4: Is it likely that assessment of the outcome was influenced by knowledge of intervention received? | | | | | | | | NA |  |  |
|  | **Risk of bias judgement** | | | | | | | | **Low** |  |  |
| **Bias in selection of the reported result** | 5.1 Were the data that produced this result analysed in accordance with a pre-specified analysis plan that was finalized before unblinded outcome data were available for analysis? | | | | | | | | NI |  |  |
|  | 5.2 ... multiple eligible outcome measurements (e.g. scales, definitions, time points) within the outcome domain? | | | | | | | | PN | No multiple outcomes measurements. |  |
|  | 5.3 ... multiple eligible analyses of the data? | | | | | | | | PN | No multiple analyses. |  |
|  | **Risk of bias judgement** | | | | | | | | **Some concerns** |  |  |
| **Overall bias** | **Risk of bias judgement** | | | | | | | | **Some concerns** |  |  |
|  |  | | |  | |  | | |  |  |  |
|  |  | | |  | |  | | |  |  |  |
| **Unique ID** | Mohammadzadeh et al. 2013 | | | **Study ID** | | Mohammadzadeh et al. 2013 | | | **Assessor** | Abdallah Saeed & Majd M. AlBarakat |  |
| **Ref or Label** | Mohammadzadeh et al. 2013 | | | **Aim** | | assignment to intervention (the 'intention-to-treat' effect) | | |  |  |  |
| **Domain** | **Signalling question** | | | | | | | | **Response** | **Comments** |  |
| **Bias arising from the randomization process** | 1.1 Was the allocation sequence random? | | | | | | | | NI |  |  |
|  | 1.2 Was the allocation sequence concealed until participants were enrolled and assigned to interventions? | | | | | | | | NI |  |  |
|  | 1.3 Did baseline differences between intervention groups suggest a problem with the randomization process? | | | | | | | | N | There are no significant imbalances |  |
|  | **Risk of bias judgement** | | | | | | | | **Some concerns** |  |  |
| **Bias due to deviations from intended interventions** | 2.1.Were participants aware of their assigned intervention during the trial? | | | | | | | | NI |  |  |
|  | 2.2.Were carers and people delivering the interventions aware of participants' assigned intervention during the trial? | | | | | | | | PY |  |  |
|  | 2.3. If Y/PY/NI to 2.1 or 2.2: Were there deviations from the intended intervention that arose because of the experimental context? | | | | | | | | NI |  |  |
|  | 2.4 If Y/PY to 2.3: Were these deviations likely to have affected the outcome? | | | | | | | | NA |  |  |
|  | 2.5. If Y/PY/NI to 2.4: Were these deviations from intended intervention balanced between groups? | | | | | | | | NA |  |  |
|  | 2.6 Was an appropriate analysis used to estimate the effect of assignment to intervention? | | | | | | | | NI |  |  |
|  | 2.7 If N/PN/NI to 2.6: Was there potential for a substantial impact (on the result) of the failure to analyse participants in the group to which they were randomized? | | | | | | | | PN |  |  |
|  | **Risk of bias judgement** | | | | | | | | **Some concerns** |  |  |
| **Bias due to missing outcome data** | 3.1 Were data for this outcome available for all, or nearly all, participants randomized? | | | | | | | | PY | The data for this outcome was available for nearly all participants who were randomized. |  |
|  | 3.2 If N/PN/NI to 3.1: Is there evidence that result was not biased by missing outcome data? | | | | | | | | NA |  |  |
|  | 3.3 If N/PN to 3.2: Could missingness in the outcome depend on its true value? | | | | | | | | NA |  |  |
|  | 3.4 If Y/PY/NI to 3.3: Is it likely that missingness in the outcome depended on its true value? | | | | | | | | NA |  |  |
|  | **Risk of bias judgement** | | | | | | | | **Low** |  |  |
| **Bias in measurement of the outcome** | 4.1 Was the method of measuring the outcome inappropriate? | | | | | | | | PN | The methods used for measuring the outcome were appropriate. |  |
|  | 4.2 Could measurement or ascertainment of the outcome have differed between intervention groups? | | | | | | | | PN | No difference in measurement was detected between the groups. |  |
|  | 4.3 Were outcome assessors aware of the intervention received by study participants? | | | | | | | | NI |  |  |
|  | 4.4 If Y/PY/NI to 4.3: Could assessment of the outcome have been influenced by knowledge of intervention received? | | | | | | | | PN |  |  |
|  | 4.5 If Y/PY/NI to 4.4: Is it likely that assessment of the outcome was influenced by knowledge of intervention received? | | | | | | | | NA |  |  |
|  | **Risk of bias judgement** | | | | | | | | **Low** |  |  |
| **Bias in selection of the reported result** | 5.1 Were the data that produced this result analysed in accordance with a pre-specified analysis plan that was finalized before unblinded outcome data were available for analysis? | | | | | | | | NI |  |  |
|  | 5.2 ... multiple eligible outcome measurements (e.g. scales, definitions, time points) within the outcome domain? | | | | | | | | PN | No multiple outcomes measurements. |  |
|  | 5.3 ... multiple eligible analyses of the data? | | | | | | | | PN | No multiple analyses. |  |
|  | **Risk of bias judgement** | | | | | | | | **Some concerns** |  |  |
| **Overall bias** | **Risk of bias judgement** | | | | | | | | **Some concerns** |  |  |
|  |  | | |  | |  | | |  |  |  |
|  |  | | |  | |  | | |  |  |  |
| **Unique ID** | Murkin et al. 1995 | | | **Study ID** | | Murkin et al. 1995 | | | **Assessor** | Abdallah Saeed & Majd M. AlBarakat |  |
| **Ref or Label** | Murkin et al. 1995 | | | **Aim** | | assignment to intervention (the 'intention-to-treat' effect) | | |  |  |  |
| **Domain** | **Signalling question** | | | | | | | | **Response** | **Comments** |  |
| **Bias arising from the randomization process** | 1.1 Was the allocation sequence random? | | | | | | | | NI |  |  |
|  | 1.2 Was the allocation sequence concealed until participants were enrolled and assigned to interventions? | | | | | | | | NI |  |  |
|  | 1.3 Did baseline differences between intervention groups suggest a problem with the randomization process? | | | | | | | | N | There are no significant imbalances |  |
|  | **Risk of bias judgement** | | | | | | | | **Some concerns** |  |  |
| **Bias due to deviations from intended interventions** | 2.1.Were participants aware of their assigned intervention during the trial? | | | | | | | | N |  |  |
|  | 2.2.Were carers and people delivering the interventions aware of participants' assigned intervention during the trial? | | | | | | | | N |  |  |
|  | 2.3. If Y/PY/NI to 2.1 or 2.2: Were there deviations from the intended intervention that arose because of the experimental context? | | | | | | | | NA | No apparent deviations were reported |  |
|  | 2.4 If Y/PY to 2.3: Were these deviations likely to have affected the outcome? | | | | | | | | NA |  |  |
|  | 2.5. If Y/PY/NI to 2.4: Were these deviations from intended intervention balanced between groups? | | | | | | | | NA |  |  |
|  | 2.6 Was an appropriate analysis used to estimate the effect of assignment to intervention? | | | | | | | | PY | Appropriate analysis was used. |  |
|  | 2.7 If N/PN/NI to 2.6: Was there potential for a substantial impact (on the result) of the failure to analyse participants in the group to which they were randomized? | | | | | | | | PN |  |  |
|  | **Risk of bias judgement** | | | | | | | | **Low** |  |  |
| **Bias due to missing outcome data** | 3.1 Were data for this outcome available for all, or nearly all, participants randomized? | | | | | | | | Y | The data for this outcome was available for nearly all participants who were randomized. |  |
|  | 3.2 If N/PN/NI to 3.1: Is there evidence that result was not biased by missing outcome data? | | | | | | | | NA |  |  |
|  | 3.3 If N/PN to 3.2: Could missingness in the outcome depend on its true value? | | | | | | | | NA |  |  |
|  | 3.4 If Y/PY/NI to 3.3: Is it likely that missingness in the outcome depended on its true value? | | | | | | | | NA |  |  |
|  | **Risk of bias judgement** | | | | | | | | **Low** |  |  |
| **Bias in measurement of the outcome** | 4.1 Was the method of measuring the outcome inappropriate? | | | | | | | | PN | The methods used for measuring the outcome were appropriate. |  |
|  | 4.2 Could measurement or ascertainment of the outcome have differed between intervention groups? | | | | | | | | PN | No difference in measurement was detected between the groups. |  |
|  | 4.3 Were outcome assessors aware of the intervention received by study participants? | | | | | | | | N |  |  |
|  | 4.4 If Y/PY/NI to 4.3: Could assessment of the outcome have been influenced by knowledge of intervention received? | | | | | | | | NA |  |  |
|  | 4.5 If Y/PY/NI to 4.4: Is it likely that assessment of the outcome was influenced by knowledge of intervention received? | | | | | | | | NA |  |  |
|  | **Risk of bias judgement** | | | | | | | | **Low** |  |  |
| **Bias in selection of the reported result** | 5.1 Were the data that produced this result analysed in accordance with a pre-specified analysis plan that was finalized before unblinded outcome data were available for analysis? | | | | | | | | NI |  |  |
|  | 5.2 ... multiple eligible outcome measurements (e.g. scales, definitions, time points) within the outcome domain? | | | | | | | | PN | No multiple outcomes measurements. |  |
|  | 5.3 ... multiple eligible analyses of the data? | | | | | | | | PN | No multiple analyses. |  |
|  | **Risk of bias judgement** | | | | | | | | **Some concerns** |  |  |
| **Overall bias** | **Risk of bias judgement** | | | | | | | | **Some concerns** |  |  |
|  |  | | |  | |  | | |  |  |  |
| **Unique ID** | O’Neil et al. 2012 | | | **Study ID** | | O’Neil et al. 2012 | | | **Assessor** | Abdallah Saeed & Majd M. AlBarakat |  |
| **Ref or Label** | O’Neil et al. 2012 | | | **Aim** | | assignment to intervention (the 'intention-to-treat' effect) | | |  |  |  |
| **Domain** | **Signalling question** | | | | | | | | **Response** | **Comments** |  |
| **Bias arising from the randomization process** | 1.1 Was the allocation sequence random? | | | | | | | | NI |  |  |
|  | 1.2 Was the allocation sequence concealed until participants were enrolled and assigned to interventions? | | | | | | | | Y |  |  |
|  | 1.3 Did baseline differences between intervention groups suggest a problem with the randomization process? | | | | | | | | N | There are no significant imbalances |  |
|  | **Risk of bias judgement** | | | | | | | | **Some concerns** |  |  |
| **Bias due to deviations from intended interventions** | 2.1.Were participants aware of their assigned intervention during the trial? | | | | | | | | NI |  |  |
|  | 2.2.Were carers and people delivering the interventions aware of participants' assigned intervention during the trial? | | | | | | | | NI |  |  |
|  | 2.3. If Y/PY/NI to 2.1 or 2.2: Were there deviations from the intended intervention that arose because of the experimental context? | | | | | | | | NI |  |  |
|  | 2.4 If Y/PY to 2.3: Were these deviations likely to have affected the outcome? | | | | | | | | NA |  |  |
|  | 2.5. If Y/PY/NI to 2.4: Were these deviations from intended intervention balanced between groups? | | | | | | | | NA |  |  |
|  | 2.6 Was an appropriate analysis used to estimate the effect of assignment to intervention? | | | | | | | | PY | Appropriate analysis was used. |  |
|  | 2.7 If N/PN/NI to 2.6: Was there potential for a substantial impact (on the result) of the failure to analyse participants in the group to which they were randomized? | | | | | | | | NA |  |  |
|  | **Risk of bias judgement** | | | | | | | | **Some concerns** |  |  |
| **Bias due to missing outcome data** | 3.1 Were data for this outcome available for all, or nearly all, participants randomized? | | | | | | | | PY | The data for this outcome was available for nearly all participants who were randomized. |  |
|  | 3.2 If N/PN/NI to 3.1: Is there evidence that result was not biased by missing outcome data? | | | | | | | | NA |  |  |
|  | 3.3 If N/PN to 3.2: Could missingness in the outcome depend on its true value? | | | | | | | | NA |  |  |
|  | 3.4 If Y/PY/NI to 3.3: Is it likely that missingness in the outcome depended on its true value? | | | | | | | | NA |  |  |
|  | **Risk of bias judgement** | | | | | | | | **Low** |  |  |
| **Bias in measurement of the outcome** | 4.1 Was the method of measuring the outcome inappropriate? | | | | | | | | PN | The methods used for measuring the outcome were appropriate. |  |
|  | 4.2 Could measurement or ascertainment of the outcome have differed between intervention groups? | | | | | | | | PN | No difference in measurement was detected between the groups. |  |
|  | 4.3 Were outcome assessors aware of the intervention received by study participants? | | | | | | | | PN |  |  |
|  | 4.4 If Y/PY/NI to 4.3: Could assessment of the outcome have been influenced by knowledge of intervention received? | | | | | | | | NA |  |  |
|  | 4.5 If Y/PY/NI to 4.4: Is it likely that assessment of the outcome was influenced by knowledge of intervention received? | | | | | | | | NA |  |  |
|  | **Risk of bias judgement** | | | | | | | | **Low** |  |  |
| **Bias in selection of the reported result** | 5.1 Were the data that produced this result analysed in accordance with a pre-specified analysis plan that was finalized before unblinded outcome data were available for analysis? | | | | | | | | NI |  |  |
|  | 5.2 ... multiple eligible outcome measurements (e.g. scales, definitions, time points) within the outcome domain? | | | | | | | | PN | No multiple outcomes measurements. |  |
|  | 5.3 ... multiple eligible analyses of the data? | | | | | | | | PN | No multiple analyses. |  |
|  | **Risk of bias judgement** | | | | | | | | **Some concerns** |  |  |
| **Overall bias** | **Risk of bias judgement** | | | | | | | | **Some concerns** |  |  |
| **Unique ID** | | O’Neil et al. 2012 | | | | | **Study ID** | | O’Neil et al. 2012 | **Assessor** | Mohamed R. Abdelraouf & Dina Aymen |
| **Ref or Label** | | O’Neil et al. 2012 | | | | | **Aim** | | assignment to intervention (the 'intention-to-treat' effect) |  |  |
| **Domain** | | **Signalling question** | | | | | | | | **Response** | **Comments** |
| **Bias arising from the randomization process** | | 1.1 Was the allocation sequence random? | | | | | | | | Y | This was a prospective randomized study |
|  |  | 1.2 Was the allocation sequence concealed until participants were enrolled and assigned to interventions? | | | | | | | | PY |  |
|  |  | 1.3 Did baseline differences between intervention groups suggest a problem with the randomization process? | | | | | | | | N | No significant imbalances was detected. |
|  |  | **Risk of bias judgement** | | | | | | | | **Low** |  |
| **Bias due to deviations from intended interventions** | | 2.1.Were participants aware of their assigned intervention during the trial? | | | | | | | | PN |  |
|  |  | 2.2.Were carers and people delivering the interventions aware of participants' assigned intervention during the trial? | | | | | | | | PY |  |
|  |  | 2.3. If Y/PY/NI to 2.1 or 2.2: Were there deviations from the intended intervention that arose because of the experimental context? | | | | | | | | PN | No apparent deviations were reported. |
|  |  | 2.4 If Y/PY to 2.3: Were these deviations likely to have affected the outcome? | | | | | | | | NA |  |
|  |  | 2.5. If Y/PY/NI to 2.4: Were these deviations from intended intervention balanced between groups? | | | | | | | | NA |  |
|  |  | 2.6 Was an appropriate analysis used to estimate the effect of assignment to intervention? | | | | | | | | PN | No appropriate analysis was used. |
|  |  | 2.7 If N/PN/NI to 2.6: Was there potential for a substantial impact (on the result) of the failure to analyse participants in the group to which they were randomized? | | | | | | | | PN |  |
|  |  | **Risk of bias judgement** | | | | | | | | **Low** |  |
| **Bias due to missing outcome data** | | 3.1 Were data for this outcome available for all, or nearly all, participants randomized? | | | | | | | | Y | The data for this outcome was available for nearly all participants who were randomized. |
|  |  | 3.2 If N/PN/NI to 3.1: Is there evidence that result was not biased by missing outcome data? | | | | | | | | NA |  |
|  |  | 3.3 If N/PN to 3.2: Could missingness in the outcome depend on its true value? | | | | | | | | NA |  |
|  |  | 3.4 If Y/PY/NI to 3.3: Is it likely that missingness in the outcome depended on its true value? | | | | | | | | NA |  |
|  |  | **Risk of bias judgement** | | | | | | | | **Low** |  |
| **Bias in measurement of the outcome** | | 4.1 Was the method of measuring the outcome inappropriate? | | | | | | | | N | The methods used for measuring the outcome were appropriate. |
|  |  | 4.2 Could measurement or ascertainment of the outcome have differed between intervention groups? | | | | | | | | N | No difference in measurement was detected between the groups. |
|  |  | 4.3 Were outcome assessors aware of the intervention received by study participants? | | | | | | | | PY |  |
|  |  | 4.4 If Y/PY/NI to 4.3: Could assessment of the outcome have been influenced by knowledge of intervention received? | | | | | | | | PN |  |
|  |  | 4.5 If Y/PY/NI to 4.4: Is it likely that assessment of the outcome was influenced by knowledge of intervention received? | | | | | | | | NA |  |
|  |  | **Risk of bias judgement** | | | | | | | | **Low** |  |
| **Bias in selection of the reported result** | | 5.1 Were the data that produced this result analysed in accordance with a pre-specified analysis plan that was finalized before unblinded outcome data were available for analysis? | | | | | | | | PY |  |
|  |  | 5.2 ... multiple eligible outcome measurements (e.g. scales, definitions, time points) within the outcome domain? | | | | | | | | N | No multiple outcomes measurements. |
|  |  | 5.3 ... multiple eligible analyses of the data? | | | | | | | | N | No multiple analyses. |
|  |  | **Risk of bias judgement** | | | | | | | | **Low** |  |
| **Overall bias** | | **Risk of bias judgement** | | | | | | | | **Low** |  |
|  | |  | | | | |  | |  |  |  |
|  | |  | | | | |  | |  |  |  |
| **Unique ID** | | O’Neil et al. 2018 | | | | | **Study ID** | | O’Neil et al. 2018 | **Assessor** | Mohamed R. Abdelraouf & Dina Aymen |
| **Ref or Label** | | O’Neil et al. 2018 | | | | | **Aim** | | assignment to intervention (the 'intention-to-treat' effect) |  |  |
| **Domain** | | **Signalling question** | | | | | | | | **Response** | **Comments** |
| **Bias arising from the randomization process** | | 1.1 Was the allocation sequence random? | | | | | | | | Y | this prospective, randomized cohort study of 20 high-risk cardiac surgical patients |
|  |  | 1.2 Was the allocation sequence concealed until participants were enrolled and assigned to interventions? | | | | | | | | PY |  |
|  |  | 1.3 Did baseline differences between intervention groups suggest a problem with the randomization process? | | | | | | | | N | There are no significant imbalances |
|  |  | **Risk of bias judgement** | | | | | | | | **Low** |  |
| **Bias due to deviations from intended interventions** | | 2.1.Were participants aware of their assigned intervention during the trial? | | | | | | | | PN |  |
|  |  | 2.2.Were carers and people delivering the interventions aware of participants' assigned intervention during the trial? | | | | | | | | PN |  |
|  |  | 2.3. If Y/PY/NI to 2.1 or 2.2: Were there deviations from the intended intervention that arose because of the experimental context? | | | | | | | | NA | No apparent deviations were reported. |
|  |  | 2.4 If Y/PY to 2.3: Were these deviations likely to have affected the outcome? | | | | | | | | NA |  |
|  |  | 2.5. If Y/PY/NI to 2.4: Were these deviations from intended intervention balanced between groups? | | | | | | | | NA |  |
|  |  | 2.6 Was an appropriate analysis used to estimate the effect of assignment to intervention? | | | | | | | | PN | No appropriate analysis was used. |
|  |  | 2.7 If N/PN/NI to 2.6: Was there potential for a substantial impact (on the result) of the failure to analyse participants in the group to which they were randomized? | | | | | | | | PN |  |
|  |  | **Risk of bias judgement** | | | | | | | | **Low** |  |
| **Bias due to missing outcome data** | | 3.1 Were data for this outcome available for all, or nearly all, participants randomized? | | | | | | | | Y | The data for this outcome was available for nearly all participants who were randomized. |
|  |  | 3.2 If N/PN/NI to 3.1: Is there evidence that result was not biased by missing outcome data? | | | | | | | | NA |  |
|  |  | 3.3 If N/PN to 3.2: Could missingness in the outcome depend on its true value? | | | | | | | | NA |  |
|  |  | 3.4 If Y/PY/NI to 3.3: Is it likely that missingness in the outcome depended on its true value? | | | | | | | | NA |  |
|  |  | **Risk of bias judgement** | | | | | | | | **Low** |  |
| **Bias in measurement of the outcome** | | 4.1 Was the method of measuring the outcome inappropriate? | | | | | | | | N | The methods used for measuring the outcome were appropriate. |
|  |  | 4.2 Could measurement or ascertainment of the outcome have differed between intervention groups? | | | | | | | | N | No difference in measurement was detected between the groups. |
|  |  | 4.3 Were outcome assessors aware of the intervention received by study participants? | | | | | | | | PY |  |
|  |  | 4.4 If Y/PY/NI to 4.3: Could assessment of the outcome have been influenced by knowledge of intervention received? | | | | | | | | PN |  |
|  |  | 4.5 If Y/PY/NI to 4.4: Is it likely that assessment of the outcome was influenced by knowledge of intervention received? | | | | | | | | NA |  |
|  |  | **Risk of bias judgement** | | | | | | | | **Low** |  |
| **Bias in selection of the reported result** | | 5.1 Were the data that produced this result analysed in accordance with a pre-specified analysis plan that was finalized before unblinded outcome data were available for analysis? | | | | | | | | NI |  |
|  |  | 5.2 ... multiple eligible outcome measurements (e.g. scales, definitions, time points) within the outcome domain? | | | | | | | | N | No multiple outcomes measurements. |
|  |  | 5.3 ... multiple eligible analyses of the data? | | | | | | | | N | No multiple analyses. |
|  |  | **Risk of bias judgement** | | | | | | | | **Some concerns** |  |
| **Overall bias** | | **Risk of bias judgement** | | | | | | | | **Some concerns** |  |
|  | |  | | | | |  | |  |  |  |
|  | |  | | | | |  | |  |  |  |
|  | |  | | | | |  | |  |  |  |
|  | |  | | | | |  | |  |  |  |
| **Unique ID** | | Onorati et al. 2007 | | | | | **Study ID** | | Onorati et al. 2007 | **Assessor** | Mohamed R. Abdelraouf & Dina Aymen |
| **Ref or Label** | | Onorati et al. 2007 | | | | | **Aim** | | assignment to intervention (the 'intention-to-treat' effect) |  |  |
| **Domain** | | **Signalling question** | | | | | | | | **Response** | **Comments** |
| **Bias arising from the randomization process** | | 1.1 Was the allocation sequence random? | | | | | | | | Y | Patients were always randomly assigned to group A or  group B by the same operator, using numbered containers  for randomization |
|  |  | 1.2 Was the allocation sequence concealed until participants were enrolled and assigned to interventions? | | | | | | | | NI |  |
|  |  | 1.3 Did baseline differences between intervention groups suggest a problem with the randomization process? | | | | | | | | N | There are no significant imbalances |
|  |  | **Risk of bias judgement** | | | | | | | | **Some concerns** |  |
| **Bias due to deviations from intended interventions** | | 2.1.Were participants aware of their assigned intervention during the trial? | | | | | | | | PY |  |
|  |  | 2.2.Were carers and people delivering the interventions aware of participants' assigned intervention during the trial? | | | | | | | | PY |  |
|  |  | 2.3. If Y/PY/NI to 2.1 or 2.2: Were there deviations from the intended intervention that arose because of the experimental context? | | | | | | | | PN | No apparent deviations were reported. |
|  |  | 2.4 If Y/PY to 2.3: Were these deviations likely to have affected the outcome? | | | | | | | | NA |  |
|  |  | 2.5. If Y/PY/NI to 2.4: Were these deviations from intended intervention balanced between groups? | | | | | | | | NA |  |
|  |  | 2.6 Was an appropriate analysis used to estimate the effect of assignment to intervention? | | | | | | | | PY |  |
|  |  | 2.7 If N/PN/NI to 2.6: Was there potential for a substantial impact (on the result) of the failure to analyse participants in the group to which they were randomized? | | | | | | | | NA |  |
|  |  | **Risk of bias judgement** | | | | | | | | **Low** |  |
| **Bias due to missing outcome data** | | 3.1 Were data for this outcome available for all, or nearly all, participants randomized? | | | | | | | | Y | The data for this outcome was available for nearly all participants who were randomized. |
|  |  | 3.2 If N/PN/NI to 3.1: Is there evidence that result was not biased by missing outcome data? | | | | | | | | NA |  |
|  |  | 3.3 If N/PN to 3.2: Could missingness in the outcome depend on its true value? | | | | | | | | NA |  |
|  |  | 3.4 If Y/PY/NI to 3.3: Is it likely that missingness in the outcome depended on its true value? | | | | | | | | NA |  |
|  |  | **Risk of bias judgement** | | | | | | | | **Low** |  |
| **Bias in measurement of the outcome** | | 4.1 Was the method of measuring the outcome inappropriate? | | | | | | | | N | The methods used for measuring the outcome were appropriate. |
|  |  | 4.2 Could measurement or ascertainment of the outcome have differed between intervention groups? | | | | | | | | N | No difference in measurement was detected between the groups. |
|  |  | 4.3 Were outcome assessors aware of the intervention received by study participants? | | | | | | | | NI |  |
|  |  | 4.4 If Y/PY/NI to 4.3: Could assessment of the outcome have been influenced by knowledge of intervention received? | | | | | | | | PN |  |
|  |  | 4.5 If Y/PY/NI to 4.4: Is it likely that assessment of the outcome was influenced by knowledge of intervention received? | | | | | | | | NA |  |
|  |  | **Risk of bias judgement** | | | | | | | | **Low** |  |
| **Bias in selection of the reported result** | | 5.1 Were the data that produced this result analysed in accordance with a pre-specified analysis plan that was finalized before unblinded outcome data were available for analysis? | | | | | | | | PY |  |
|  |  | 5.2 ... multiple eligible outcome measurements (e.g. scales, definitions, time points) within the outcome domain? | | | | | | | | N | No multiple outcomes measurements. |
|  |  | 5.3 ... multiple eligible analyses of the data? | | | | | | | | N | No multiple analyses. |
|  |  | **Risk of bias judgement** | | | | | | | | **Low** |  |
| **Overall bias** | | **Risk of bias judgement** | | | | | | | | **Some concerns** |  |
|  | |  | | | | |  | |  |  |  |
|  | |  | | | | |  | |  |  |  |
| **Unique ID** | | Onorati et al. 2009 a | | | | | **Study ID** | | Onorati et al. 2009 a | **Assessor** | Mohamed R. Abdelraouf & Dina Aymen |
| **Ref or Label** | | Onorati et al. 2009 a | | | | | **Aim** | | assignment to intervention (the 'intention-to-treat' effect) |  |  |
| **Domain** | | **Signalling question** | | | | | | | | **Response** | **Comments** |
| **Bias arising from the randomization process** | | 1.1 Was the allocation sequence random? | | | | | | | | Y | Patients were randomized by lottery, drawing pre-prepared sealed envelopes containing the Group Assignment |
|  |  | 1.2 Was the allocation sequence concealed until participants were enrolled and assigned to interventions? | | | | | | | | NI |  |
|  |  | 1.3 Did baseline differences between intervention groups suggest a problem with the randomization process? | | | | | | | | N | There are no significant imbalances |
|  |  | **Risk of bias judgement** | | | | | | | | **Some concerns** |  |
| **Bias due to deviations from intended interventions** | | 2.1.Were participants aware of their assigned intervention during the trial? | | | | | | | | PN |  |
|  |  | 2.2.Were carers and people delivering the interventions aware of participants' assigned intervention during the trial? | | | | | | | | PY |  |
|  |  | 2.3. If Y/PY/NI to 2.1 or 2.2: Were there deviations from the intended intervention that arose because of the experimental context? | | | | | | | | PN | No apparent deviations were reported. |
|  |  | 2.4 If Y/PY to 2.3: Were these deviations likely to have affected the outcome? | | | | | | | | NA |  |
|  |  | 2.5. If Y/PY/NI to 2.4: Were these deviations from intended intervention balanced between groups? | | | | | | | | NA |  |
|  |  | 2.6 Was an appropriate analysis used to estimate the effect of assignment to intervention? | | | | | | | | Y | . |
|  |  | 2.7 If N/PN/NI to 2.6: Was there potential for a substantial impact (on the result) of the failure to analyse participants in the group to which they were randomized? | | | | | | | | NA |  |
|  |  | **Risk of bias judgement** | | | | | | | | **Low** |  |
| **Bias due to missing outcome data** | | 3.1 Were data for this outcome available for all, or nearly all, participants randomized? | | | | | | | | Y | The data for this outcome was available for nearly all participants who were randomized. |
|  |  | 3.2 If N/PN/NI to 3.1: Is there evidence that result was not biased by missing outcome data? | | | | | | | | NA |  |
|  |  | 3.3 If N/PN to 3.2: Could missingness in the outcome depend on its true value? | | | | | | | | NA |  |
|  |  | 3.4 If Y/PY/NI to 3.3: Is it likely that missingness in the outcome depended on its true value? | | | | | | | | NA |  |
|  |  | **Risk of bias judgement** | | | | | | | | **Low** |  |
| **Bias in measurement of the outcome** | | 4.1 Was the method of measuring the outcome inappropriate? | | | | | | | | N | The methods used for measuring the outcome were appropriate. |
|  |  | 4.2 Could measurement or ascertainment of the outcome have differed between intervention groups? | | | | | | | | N | No difference in measurement was detected between the groups. |
|  |  | 4.3 Were outcome assessors aware of the intervention received by study participants? | | | | | | | | PY |  |
|  |  | 4.4 If Y/PY/NI to 4.3: Could assessment of the outcome have been influenced by knowledge of intervention received? | | | | | | | | PN |  |
|  |  | 4.5 If Y/PY/NI to 4.4: Is it likely that assessment of the outcome was influenced by knowledge of intervention received? | | | | | | | | NA |  |
|  |  | **Risk of bias judgement** | | | | | | | | **Low** |  |
| **Bias in selection of the reported result** | | 5.1 Were the data that produced this result analysed in accordance with a pre-specified analysis plan that was finalized before unblinded outcome data were available for analysis? | | | | | | | | NI |  |
|  |  | 5.2 ... multiple eligible outcome measurements (e.g. scales, definitions, time points) within the outcome domain? | | | | | | | | N | No multiple outcomes measurements. |
|  |  | 5.3 ... multiple eligible analyses of the data? | | | | | | | | N | No multiple analyses. |
|  |  | **Risk of bias judgement** | | | | | | | | **Some concerns** |  |
| **Overall bias** | | **Risk of bias judgement** | | | | | | | | **Some concerns** |  |
|  | |  | | | | |  | |  |  |  |
|  | |  | | | | |  | |  |  |  |
| **Unique ID** | | Onorati et al. 2009 b | | | | | **Study ID** | | Onorati et al. 2009 b | **Assessor** | Mohamed R. Abdelraouf & Dina Aymen |
| **Ref or Label** | | Onorati et al. 2009 b | | | | | **Aim** | | assignment to intervention (the 'intention-to-treat' effect) |  |  |
| **Domain** | | **Signalling question** | | | | | | | | **Response** | **Comments** |
| **Bias arising from the randomization process** | | 1.1 Was the allocation sequence random? | | | | | | | | Y | the patients were randomized by lottery, drawing pre-prepared sealed envelopes containing the group assignment |
|  |  | 1.2 Was the allocation sequence concealed until participants were enrolled and assigned to interventions? | | | | | | | | PY |  |
|  |  | 1.3 Did baseline differences between intervention groups suggest a problem with the randomization process? | | | | | | | | N | There are no significant imbalances |
|  |  | **Risk of bias judgement** | | | | | | | | **Low** |  |
| **Bias due to deviations from intended interventions** | | 2.1.Were participants aware of their assigned intervention during the trial? | | | | | | | | PN |  |
|  |  | 2.2.Were carers and people delivering the interventions aware of participants' assigned intervention during the trial? | | | | | | | | PY |  |
|  |  | 2.3. If Y/PY/NI to 2.1 or 2.2: Were there deviations from the intended intervention that arose because of the experimental context? | | | | | | | | PN | No apparent deviations were reported. |
|  |  | 2.4 If Y/PY to 2.3: Were these deviations likely to have affected the outcome? | | | | | | | | NA |  |
|  |  | 2.5. If Y/PY/NI to 2.4: Were these deviations from intended intervention balanced between groups? | | | | | | | | NA |  |
|  |  | 2.6 Was an appropriate analysis used to estimate the effect of assignment to intervention? | | | | | | | | PN | No appropriate analysis was used. |
|  |  | 2.7 If N/PN/NI to 2.6: Was there potential for a substantial impact (on the result) of the failure to analyse participants in the group to which they were randomized? | | | | | | | | N |  |
|  |  | **Risk of bias judgement** | | | | | | | | **Low** |  |
| **Bias due to missing outcome data** | | 3.1 Were data for this outcome available for all, or nearly all, participants randomized? | | | | | | | | Y | The data for this outcome was available for nearly all participants who were randomized. |
|  |  | 3.2 If N/PN/NI to 3.1: Is there evidence that result was not biased by missing outcome data? | | | | | | | | NA |  |
|  |  | 3.3 If N/PN to 3.2: Could missingness in the outcome depend on its true value? | | | | | | | | NA |  |
|  |  | 3.4 If Y/PY/NI to 3.3: Is it likely that missingness in the outcome depended on its true value? | | | | | | | | NA |  |
|  |  | **Risk of bias judgement** | | | | | | | | **Low** |  |
| **Bias in measurement of the outcome** | | 4.1 Was the method of measuring the outcome inappropriate? | | | | | | | | N | The methods used for measuring the outcome were appropriate. |
|  |  | 4.2 Could measurement or ascertainment of the outcome have differed between intervention groups? | | | | | | | | N | No difference in measurement was detected between the groups. |
|  |  | 4.3 Were outcome assessors aware of the intervention received by study participants? | | | | | | | | PN |  |
|  |  | 4.4 If Y/PY/NI to 4.3: Could assessment of the outcome have been influenced by knowledge of intervention received? | | | | | | | | NA |  |
|  |  | 4.5 If Y/PY/NI to 4.4: Is it likely that assessment of the outcome was influenced by knowledge of intervention received? | | | | | | | | NA |  |
|  |  | **Risk of bias judgement** | | | | | | | | **Low** |  |
| **Bias in selection of the reported result** | | 5.1 Were the data that produced this result analysed in accordance with a pre-specified analysis plan that was finalized before unblinded outcome data were available for analysis? | | | | | | | | NI |  |
|  |  | 5.2 ... multiple eligible outcome measurements (e.g. scales, definitions, time points) within the outcome domain? | | | | | | | | N | No multiple outcomes measurements. |
|  |  | 5.3 ... multiple eligible analyses of the data? | | | | | | | | N | No multiple analyses. |
|  |  | **Risk of bias judgement** | | | | | | | | **Low** |  |
| **Overall bias** | | **Risk of bias judgement** | | | | | | | | **Low** |  |
|  | |  | | | | |  | |  |  |  |
|  | |  | | | | |  | |  |  |  |
| **Unique ID** | | Poswal et al. 2004 | | | | | **Study ID** | | Poswal et al. 2004 | **Assessor** | Mohamed R. Abdelraouf & Dina Aymen |
| **Ref or Label** | | Poswal et al. 2004 | | | | | **Aim** | | assignment to intervention (the 'intention-to-treat' effect) |  |  |
| **Domain** | | **Signalling question** | | | | | | | | **Response** | **Comments** |
| **Bias arising from the randomization process** | | 1.1 Was the allocation sequence random? | | | | | | | | Y |  |
|  |  | 1.2 Was the allocation sequence concealed until participants were enrolled and assigned to interventions? | | | | | | | | NI |  |
|  |  | 1.3 Did baseline differences between intervention groups suggest a problem with the randomization process? | | | | | | | | N | There are no significant imbalances |
|  |  | **Risk of bias judgement** | | | | | | | | **Some concerns** |  |
| **Bias due to deviations from intended interventions** | | 2.1.Were participants aware of their assigned intervention during the trial? | | | | | | | | PN |  |
|  |  | 2.2.Were carers and people delivering the interventions aware of participants' assigned intervention during the trial? | | | | | | | | PY |  |
|  |  | 2.3. If Y/PY/NI to 2.1 or 2.2: Were there deviations from the intended intervention that arose because of the experimental context? | | | | | | | | PN | No apparent deviations were reported. |
|  |  | 2.4 If Y/PY to 2.3: Were these deviations likely to have affected the outcome? | | | | | | | | NA |  |
|  |  | 2.5. If Y/PY/NI to 2.4: Were these deviations from intended intervention balanced between groups? | | | | | | | | NA |  |
|  |  | 2.6 Was an appropriate analysis used to estimate the effect of assignment to intervention? | | | | | | | | PN | No appropriate analysis was used. |
|  |  | 2.7 If N/PN/NI to 2.6: Was there potential for a substantial impact (on the result) of the failure to analyse participants in the group to which they were randomized? | | | | | | | | PN |  |
|  |  | **Risk of bias judgement** | | | | | | | | **Low** |  |
| **Bias due to missing outcome data** | | 3.1 Were data for this outcome available for all, or nearly all, participants randomized? | | | | | | | | Y | The data for this outcome was available for nearly all participants who were randomized. |
|  |  | 3.2 If N/PN/NI to 3.1: Is there evidence that result was not biased by missing outcome data? | | | | | | | | NA |  |
|  |  | 3.3 If N/PN to 3.2: Could missingness in the outcome depend on its true value? | | | | | | | | NA |  |
|  |  | 3.4 If Y/PY/NI to 3.3: Is it likely that missingness in the outcome depended on its true value? | | | | | | | | NA |  |
|  |  | **Risk of bias judgement** | | | | | | | | **Low** |  |
| **Bias in measurement of the outcome** | | 4.1 Was the method of measuring the outcome inappropriate? | | | | | | | | N | The methods used for measuring the outcome were appropriate. |
|  |  | 4.2 Could measurement or ascertainment of the outcome have differed between intervention groups? | | | | | | | | N | No difference in measurement was detected between the groups. |
|  |  | 4.3 Were outcome assessors aware of the intervention received by study participants? | | | | | | | | PY |  |
|  |  | 4.4 If Y/PY/NI to 4.3: Could assessment of the outcome have been influenced by knowledge of intervention received? | | | | | | | | PN |  |
|  |  | 4.5 If Y/PY/NI to 4.4: Is it likely that assessment of the outcome was influenced by knowledge of intervention received? | | | | | | | | NA |  |
|  |  | **Risk of bias judgement** | | | | | | | | **Low** |  |
| **Bias in selection of the reported result** | | 5.1 Were the data that produced this result analysed in accordance with a pre-specified analysis plan that was finalized before unblinded outcome data were available for analysis? | | | | | | | | PY |  |
|  |  | 5.2 ... multiple eligible outcome measurements (e.g. scales, definitions, time points) within the outcome domain? | | | | | | | | N | No multiple outcomes measurements. |
|  |  | 5.3 ... multiple eligible analyses of the data? | | | | | | | | N | No multiple analyses. |
|  |  | **Risk of bias judgement** | | | | | | | | **Low** |  |
| **Overall bias** | | **Risk of bias judgement** | | | | | | | | **Some concerns** |  |

| **Unique ID** | Serraino et al. 2012 | **Study ID** | Serraino et al. 2012 |  | Abdelrahman Hassan & Basant Katamesh |
| --- | --- | --- | --- | --- | --- |
| **Ref or Label** | Serraino et al. 2012 | **Aim** | assignment to intervention (the 'intention-to-treat' effect) |  |  |
| **Domain** | **Signalling question** | | | **Response** | **Comments** |
| **Bias arising from the randomization process** | 1.1 Was the allocation sequence random? | | | Y | Patients were randomized into two groups. |
|  | 1.2 Was the allocation sequence concealed until participants were enrolled and assigned to interventions? | | | NI |  |
|  | 1.3 Did baseline differences between intervention groups suggest a problem with the randomization process? | | | PN | No significant imbalances. |
|  | **Risk of bias judgement** | | | **Low** |  |
| **Bias due to deviations from intended interventions** | 2.1. Were participants aware of their assigned intervention during the trial? | | | NI |  |
|  | 2.2. Were carers and people delivering the interventions aware of participants' assigned intervention during the trial? | | | NI |  |
|  | 2.3. If Y/PY/NI to 2.1 or 2.2: Were there deviations from the intended intervention that arose because of the experimental context? | | | PN | No apparent deviations were reported. |
|  | 2.4 If Y/PY to 2.3: Were these deviations likely to have affected the outcome? | | | NA |  |
|  | 2.5. If Y/PY/NI to 2.4: Were these deviations from the intended intervention balanced between groups? | | | PN |  |
|  | 2.6 Was an appropriate analysis used to estimate the effect of assignment to intervention? | | | PY |  |
|  | 2.7 If N/PN/NI to 2.6: Was there potential for a substantial impact (on the result) of the failure to analyze participants in the group to which they were randomized? | | | PN |  |
|  | **Risk of bias judgement** | | | **Low** |  |
| **Bias due to missing outcome data** | 3.1 Were data for this outcome available for all, or nearly all, participants randomized? | | | PY | The data for this outcome was available for nearly all participants who were randomized. |
|  | 3.2 If N/PN/NI to 3.1: Is there evidence that result was not biased by missing outcome data? | | | NA |  |
|  | 3.3 If N/PN to 3.2: Could missingness in the outcome depend on its true value? | | | NA |  |
|  | 3.4 If Y/PY/NI to 3.3: Is it likely that missingness in the outcome depended on its true value? | | | NA |  |
|  | **Risk of bias judgement** | | | **Low** |  |
| **Bias in measurement of the outcome** | 4.1 Was the method of measuring the outcome inappropriate? | | | PN | The methods used for measuring the outcome were appropriate. |
|  | 4.2 Could measurement or ascertainment of the outcome have differed between intervention groups? | | | PN | No difference in measurement was detected between the groups. |
|  | 4.3 Were outcome assessors aware of the intervention received by study participants? | | | NI |  |
|  | 4.4 If Y/PY/NI to 4.3: Could assessment of the outcome have been influenced by knowledge of intervention received? | | | PN |  |
|  | 4.5 If Y/PY/NI to 4.4: Is it likely that assessment of the outcome was influenced by knowledge of intervention received? | | | NA |  |
|  | **Risk of bias judgement** | | | **Low** |  |
| **Bias in selection of the reported result** | 5.1 Were the data that produced this result analysed in accordance with a pre-specified analysis plan that was finalized before unblinded outcome data were available for analysis? | | | NI |  |
|  | 5.2 ... multiple eligible outcome measurements (e.g. scales, definitions, time points) within the outcome domain? | | | PN | No multiple outcomes measurements. |
|  | 5.3 ... multiple eligible analyses of the data? | | | PN | No multiple analyses. |
|  | **Risk of bias judgement** | | | **Low** |  |
| **Overall bias** | **Risk of bias judgement** | | | **Low** |  |
|  |  |  |  |  |  |
|  |  |  |  |  |  |
| **Unique ID** | Sezai et al. 2005 | **Study ID** | Sezai et al. 2005 |  | Abdelrahman Hassan & Basant Katamesh |
| **Ref or Label** | Sezai et al. 2005 | **Aim** | assignment to intervention (the 'intention-to-treat' effect) |  |  |
| **Domain** | **Signalling question** | | | **Response** | **Comments** |
| **Bias arising from the randomization process** | 1.1 Was the allocation sequence random? | | | Y | Patients were randomized |
|  | 1.2 Was the allocation sequence concealed until participants were enrolled and assigned to interventions? | | | NI |  |
|  | 1.3 Did baseline differences between intervention groups suggest a problem with the randomization process? | | | N | There are no significant imbalances |
|  | **Risk of bias judgement** | | | **Some concerns** |  |
| **Bias due to deviations from intended interventions** | 2.1. Were participants aware of their assigned intervention during the trial? | | | NI |  |
|  | 2.2. Were carers and people delivering the interventions aware of participants' assigned intervention during the trial? | | | NI |  |
|  | 2.3. If Y/PY/NI to 2.1 or 2.2: Were there deviations from the intended intervention that arose because of the experimental context? | | | PN | No apparent deviations were reported. |
|  | 2.4 If Y/PY to 2.3: Were these deviations likely to have affected the outcome? | | | NA |  |
|  | 2.5. If Y/PY/NI to 2.4: Were these deviations from the intended intervention balanced between groups? | | | NA |  |
|  | 2.6 Was an appropriate analysis used to estimate the effect of assignment to intervention? | | | PY |  |
|  | 2.7 If N/PN/NI to 2.6: Was there potential for a substantial impact (on the result) of the failure to analyse participants in the group to which they were randomized? | | | PN |  |
|  | **Risk of bias judgement** | | | **Low** |  |
| **Bias due to missing outcome data** | 3.1 Were data for this outcome available for all, or nearly all, participants randomized? | | | PY | The data for this outcome was available for nearly all participants who were randomized. |
|  | 3.2 If N/PN/NI to 3.1: Is there evidence that result was not biased by missing outcome data? | | | NA |  |
|  | 3.3 If N/PN to 3.2: Could missingness in the outcome depend on its true value? | | | NA |  |
|  | 3.4 If Y/PY/NI to 3.3: Is it likely that missingness in the outcome depended on its true value? | | | NA |  |
|  | **Risk of bias judgement** | | | **Low** |  |
| **Bias in measurement of the outcome** | 4.1 Was the method of measuring the outcome inappropriate? | | | PN | The methods used for measuring the outcome were appropriate. |
|  | 4.2 Could measurement or ascertainment of the outcome have differed between intervention groups? | | | PN | No difference in measurement was detected between the groups. |
|  | 4.3 Were outcome assessors aware of the intervention received by study participants? | | | NI |  |
|  | 4.4 If Y/PY/NI to 4.3: Could assessment of the outcome have been influenced by knowledge of intervention received? | | | PN |  |
|  | 4.5 If Y/PY/NI to 4.4: Is it likely that assessment of the outcome was influenced by knowledge of intervention received? | | | NA |  |
|  | **Risk of bias judgement** | | | **Low** |  |
| **Bias in selection of the reported result** | 5.1 Were the data that produced this result analysed in accordance with a pre-specified analysis plan that was finalized before unblinded outcome data were available for analysis? | | | PY |  |
|  | 5.2 ... multiple eligible outcome measurements (e.g. scales, definitions, time points) within the outcome domain? | | | PN | No multiple outcomes measurements. |
|  | 5.3 ... multiple eligible analyses of the data? | | | PN | No multiple analyses. |
|  | **Risk of bias judgement** | | | **Low** |  |
| **Overall bias** | **Risk of bias judgement** | | | **Some concerns** |  |
|  |  |  |  |  |  |
|  |  |  |  |  |  |
| **Unique ID** | Shahandashti et al. 2023 | **Study ID** | Shahandashti et al. 2023 |  | Abdelrahman Hassan & Basant Katamesh |
| **Ref or Label** | Shahandashti et al. 2023 | **Aim** | assignment to intervention (the 'intention-to-treat' effect) |  |  |
| **Domain** | **Signalling question** | | | **Response** | **Comments** |
| **Bias arising from the randomization process** | 1.1 Was the allocation sequence random? | | | NI | Patients were randomly divided into two groups patients. |
|  | 1.2 Was the allocation sequence concealed until participants were enrolled and assigned to interventions? | | | NI |  |
|  | 1.3 Did baseline differences between intervention groups suggest a problem with the randomization process? | | | PN | There are no significant imbalances |
|  | **Risk of bias judgement** | | | **Some concerns** |  |
| **Bias due to deviations from intended interventions** | 2.1.Were participants aware of their assigned intervention during the trial? | | | NI |  |
|  | 2.2.Were carers and people delivering the interventions aware of participants' assigned intervention during the trial? | | | NI |  |
|  | 2.3. If Y/PY/NI to 2.1 or 2.2: Were there deviations from the intended intervention that arose because of the experimental context? | | | PN | No apparent deviations were reported. |
|  | 2.4 If Y/PY to 2.3: Were these deviations likely to have affected the outcome? | | | NA |  |
|  | 2.5. If Y/PY/NI to 2.4: Were these deviations from intended intervention balanced between groups? | | | NA |  |
|  | 2.6 Was an appropriate analysis used to estimate the effect of assignment to intervention? | | | PY |  |
|  | 2.7 If N/PN/NI to 2.6: Was there potential for a substantial impact (on the result) of the failure to analyse participants in the group to which they were randomized? | | | PN |  |
|  | **Risk of bias judgement** | | | **Low** |  |
| **Bias due to missing outcome data** | 3.1 Were data for this outcome available for all, or nearly all, participants randomized? | | | PY | The data for this outcome was available for nearly all participants who were randomized. |
|  | 3.2 If N/PN/NI to 3.1: Is there evidence that result was not biased by missing outcome data? | | | NA |  |
|  | 3.3 If N/PN to 3.2: Could missingness in the outcome depend on its true value? | | | NA |  |
|  | 3.4 If Y/PY/NI to 3.3: Is it likely that missingness in the outcome depended on its true value? | | | NA |  |
|  | **Risk of bias judgement** | | | **Low** |  |
| **Bias in measurement of the outcome** | 4.1 Was the method of measuring the outcome inappropriate? | | | PN | The methods used for measuring the outcome were appropriate. |
|  | 4.2 Could measurement or ascertainment of the outcome have differed between intervention groups? | | | PN | No difference in measurement was detected between the groups. |
|  | 4.3 Were outcome assessors aware of the intervention received by study participants? | | | NI |  |
|  | 4.4 If Y/PY/NI to 4.3: Could assessment of the outcome have been influenced by knowledge of intervention received? | | | PN |  |
|  | 4.5 If Y/PY/NI to 4.4: Is it likely that assessment of the outcome was influenced by knowledge of intervention received? | | | NA |  |
|  | **Risk of bias judgement** | | | **Low** |  |
| **Bias in selection of the reported result** | 5.1 Were the data that produced this result analysed in accordance with a pre-specified analysis plan that was finalized before unblinded outcome data were available for analysis? | | | PY |  |
|  | 5.2 ... multiple eligible outcome measurements (e.g. scales, definitions, time points) within the outcome domain? | | | PN | No multiple outcomes measurements. |
|  | 5.3 ... multiple eligible analyses of the data? | | | PN | No multiple analyses. |
|  | **Risk of bias judgement** | | | **Low** |  |
| **Overall bias** | **Risk of bias judgement** | | | **Some concerns** |  |
|  |  |  |  |  |  |
|  |  |  |  |  |  |
| **Unique ID** | Tarcan et al. 2004 | **Study ID** | Tarcan et al. 2004 |  | Abdelrahman Hassan & Basant Katamesh |
| **Ref or Label** | Tarcan et al. 2004 | **Aim** | assignment to intervention (the 'intention-to-treat' effect) |  |  |
| **Domain** | **Signalling question** | | | **Response** | **Comments** |
| **Bias arising from the randomization process** | 1.1 Was the allocation sequence random? | | | NI | The patients were assigned to the  two groups randomly |
|  | 1.2 Was the allocation sequence concealed until participants were enrolled and assigned to interventions? | | | NI |  |
|  | 1.3 Did baseline differences between intervention groups suggest a problem with the randomization process? | | | PN | There are no significant imbalances |
|  | **Risk of bias judgement** | | | **Some concerns** |  |
| **Bias due to deviations from intended interventions** | 2.1.Were participants aware of their assigned intervention during the trial? | | | NI |  |
|  | 2.2.Were carers and people delivering the interventions aware of participants' assigned intervention during the trial? | | | NI |  |
|  | 2.3. If Y/PY/NI to 2.1 or 2.2: Were there deviations from the intended intervention that arose because of the experimental context? | | | PN | No apparent deviations were reported. |
|  | 2.4 If Y/PY to 2.3: Were these deviations likely to have affected the outcome? | | | NA |  |
|  | 2.5. If Y/PY/NI to 2.4: Were these deviations from intended intervention balanced between groups? | | | NA |  |
|  | 2.6 Was an appropriate analysis used to estimate the effect of assignment to intervention? | | | PY |  |
|  | 2.7 If N/PN/NI to 2.6: Was there potential for a substantial impact (on the result) of the failure to analyse participants in the group to which they were randomized? | | | PN |  |
|  | **Risk of bias judgement** | | | **Low** |  |
| **Bias due to missing outcome data** | 3.1 Were data for this outcome available for all, or nearly all, participants randomized? | | | PY | The data for this outcome was available for nearly all participants who were randomized. |
|  | 3.2 If N/PN/NI to 3.1: Is there evidence that result was not biased by missing outcome data? | | | NA |  |
|  | 3.3 If N/PN to 3.2: Could missingness in the outcome depend on its true value? | | | NA |  |
|  | 3.4 If Y/PY/NI to 3.3: Is it likely that missingness in the outcome depended on its true value? | | | NA |  |
|  | **Risk of bias judgement** | | | **Low** |  |
| **Bias in measurement of the outcome** | 4.1 Was the method of measuring the outcome inappropriate? | | | PN | The methods used for measuring the outcome were appropriate. |
|  | 4.2 Could measurement or ascertainment of the outcome have differed between intervention groups? | | | PN | No difference in measurement was detected between the groups. |
|  | 4.3 Were outcome assessors aware of the intervention received by study participants? | | | NI |  |
|  | 4.4 If Y/PY/NI to 4.3: Could assessment of the outcome have been influenced by knowledge of intervention received? | | | PN |  |
|  | 4.5 If Y/PY/NI to 4.4: Is it likely that assessment of the outcome was influenced by knowledge of intervention received? | | | NA |  |
|  | **Risk of bias judgement** | | | **Low** |  |
| **Bias in selection of the reported result** | 5.1 Were the data that produced this result analysed in accordance with a pre-specified analysis plan that was finalized before unblinded outcome data were available for analysis? | | | PY |  |
|  | 5.2 ... multiple eligible outcome measurements (e.g. scales, definitions, time points) within the outcome domain? | | | PN | No multiple outcomes measurements. |
|  | 5.3 ... multiple eligible analyses of the data? | | | PN | No multiple analyses. |
|  | **Risk of bias judgement** | | | **Low** |  |
| **Overall bias** | **Risk of bias judgement** | | | **Some concerns** |  |
|  |  |  |  |  |  |
|  |  |  |  |  |  |
| **Unique ID** | Ulus et al. 2023 | **Study ID** | Ulus et al. 2023 | **Assessor** | Abdelrahman Hassan & Basant Katamesh |
| **Ref or Label** | Ulus et al. 2023 | **Aim** | assignment to intervention (the 'intention-to-treat' effect) |  |  |
| **Domain** | **Signalling question** | | | **Response** | **Comments** |
| **Bias arising from the randomization process** | 1.1 Was the allocation sequence random? | | | NI | Twelve patients who had open heart surgery were randomly  divided into two groups. |
|  | 1.2 Was the allocation sequence concealed until participants were enrolled and assigned to interventions? | | | NI |  |
|  | 1.3 Did baseline differences between intervention groups suggest a problem with the randomization process? | | | PN | There are no significant imbalances |
|  | **Risk of bias judgement** | | | **Some concerns** |  |
| **Bias due to deviations from intended interventions** | 2.1.Were participants aware of their assigned intervention during the trial? | | | NI |  |
|  | 2.2.Were carers and people delivering the interventions aware of participants' assigned intervention during the trial? | | | NI |  |
|  | 2.3. If Y/PY/NI to 2.1 or 2.2: Were there deviations from the intended intervention that arose because of the experimental context? | | | PN | No apparent deviations were reported. |
|  | 2.4 If Y/PY to 2.3: Were these deviations likely to have affected the outcome? | | | NA |  |
|  | 2.5. If Y/PY/NI to 2.4: Were these deviations from intended intervention balanced between groups? | | | NA |  |
|  | 2.6 Was an appropriate analysis used to estimate the effect of assignment to intervention? | | | PY |  |
|  | 2.7 If N/PN/NI to 2.6: Was there potential for a substantial impact (on the result) of the failure to analyse participants in the group to which they were randomized? | | | PN |  |
|  | **Risk of bias judgement** | | | **Low** |  |
| **Bias due to missing outcome data** | 3.1 Were data for this outcome available for all, or nearly all, participants randomized? | | | PY | The data for this outcome was available for nearly all participants who were randomized. |
|  | 3.2 If N/PN/NI to 3.1: Is there evidence that result was not biased by missing outcome data? | | | NA |  |
|  | 3.3 If N/PN to 3.2: Could missingness in the outcome depend on its true value? | | | NA |  |
|  | 3.4 If Y/PY/NI to 3.3: Is it likely that missingness in the outcome depended on its true value? | | | NA |  |
|  | **Risk of bias judgement** | | | **Low** |  |
| **Bias in measurement of the outcome** | 4.1 Was the method of measuring the outcome inappropriate? | | | PN | The methods used for measuring the outcome were appropriate. |
|  | 4.2 Could measurement or ascertainment of the outcome have differed between intervention groups? | | | PN | No difference in measurement was detected between the groups. |
|  | 4.3 Were outcome assessors aware of the intervention received by study participants? | | | NI |  |
|  | 4.4 If Y/PY/NI to 4.3: Could assessment of the outcome have been influenced by knowledge of intervention received? | | | PN |  |
|  | 4.5 If Y/PY/NI to 4.4: Is it likely that assessment of the outcome was influenced by knowledge of intervention received? | | | NA |  |
|  | **Risk of bias judgement** | | | **Low** |  |
| **Bias in selection of the reported result** | 5.1 Were the data that produced this result analysed in accordance with a pre-specified analysis plan that was finalized before unblinded outcome data were available for analysis? | | | PY |  |
|  | 5.2 ... multiple eligible outcome measurements (e.g. scales, definitions, time points) within the outcome domain? | | | PN | No multiple outcomes measurements. |
|  | 5.3 ... multiple eligible analyses of the data? | | | PN | No multiple analyses. |
|  | **Risk of bias judgement** | | | **Low** |  |
| **Overall bias** | **Risk of bias judgement** | | | **Some concerns** |  |
|  |  |  |  |  |  |
|  |  |  |  |  |  |
| **Unique ID** | Ündar et al. 2022 | **Study ID** | Ündar et al. 2022 | **Assessor** | Abdelrahman Hassan & Basant Katamesh |
| **Ref or Label** | Ündar et al. 2022 | **Aim** | assignment to intervention (the 'intention-to-treat' effect) |  |  |
| **Domain** | **Signalling question** | | | **Response** | **Comments** |
| **Bias arising from the randomization process** | 1.1 Was the allocation sequence random? | | | Y |  |
|  | 1.2 Was the allocation sequence concealed until participants were enrolled and assigned to interventions? | | | NI |  |
|  | 1.3 Did baseline differences between intervention groups suggest a problem with the randomization process? | | | N | There are no significant imbalances |
|  | **Risk of bias judgement** | | | **Some concerns** |  |
| **Bias due to deviations from intended interventions** | 2.1.Were participants aware of their assigned intervention during the trial? | | | NI |  |
|  | 2.2.Were carers and people delivering the interventions aware of participants' assigned intervention during the trial? | | | NI |  |
|  | 2.3. If Y/PY/NI to 2.1 or 2.2: Were there deviations from the intended intervention that arose because of the experimental context? | | | PN | No apparent deviations were reported. |
|  | 2.4 If Y/PY to 2.3: Were these deviations likely to have affected the outcome? | | | NA |  |
|  | 2.5. If Y/PY/NI to 2.4: Were these deviations from intended intervention balanced between groups? | | | NA |  |
|  | 2.6 Was an appropriate analysis used to estimate the effect of assignment to intervention? | | | PY |  |
|  | 2.7 If N/PN/NI to 2.6: Was there potential for a substantial impact (on the result) of the failure to analyse participants in the group to which they were randomized? | | | PN |  |
|  | **Risk of bias judgement** | | | **Low** |  |
| **Bias due to missing outcome data** | 3.1 Were data for this outcome available for all, or nearly all, participants randomized? | | | PY | The data for this outcome was available for nearly all participants who were randomized. |
|  | 3.2 If N/PN/NI to 3.1: Is there evidence that result was not biased by missing outcome data? | | | NA |  |
|  | 3.3 If N/PN to 3.2: Could missingness in the outcome depend on its true value? | | | NA |  |
|  | 3.4 If Y/PY/NI to 3.3: Is it likely that missingness in the outcome depended on its true value? | | | NA |  |
|  | **Risk of bias judgement** | | | **Low** |  |
| **Bias in measurement of the outcome** | 4.1 Was the method of measuring the outcome inappropriate? | | | PN | The methods used for measuring the outcome were appropriate. |
|  | 4.2 Could measurement or ascertainment of the outcome have differed between intervention groups? | | | PN | No difference in measurement was detected between the groups. |
|  | 4.3 Were outcome assessors aware of the intervention received by study participants? | | | NI |  |
|  | 4.4 If Y/PY/NI to 4.3: Could assessment of the outcome have been influenced by knowledge of intervention received? | | | PN |  |
|  | 4.5 If Y/PY/NI to 4.4: Is it likely that assessment of the outcome was influenced by knowledge of intervention received? | | | NA |  |
|  | **Risk of bias judgement** | | | **Low** |  |
| **Bias in selection of the reported result** | 5.1 Were the data that produced this result analysed in accordance with a pre-specified analysis plan that was finalized before unblinded outcome data were available for analysis? | | | PY |  |
|  | 5.2 ... multiple eligible outcome measurements (e.g. scales, definitions, time points) within the outcome domain? | | | PN | No multiple outcomes measurements. |
|  | 5.3 ... multiple eligible analyses of the data? | | | PN | No multiple analyses. |
|  | **Risk of bias judgement** | | | **Low** |  |
| **Overall bias** | **Risk of bias judgement** | | | **Some concerns** |  |
|  |  |  |  |  |  |
|  |  |  |  |  |  |
| **Unique ID** | Zavareh et al. 2018 | **Study ID** | Zavareh et al. 2018 | **Assessor** | Abdelrahman Hassan & Basant Katamesh |
| **Ref or Label** | Zavareh et al. 2018 | **Aim** | assignment to intervention (the 'intention-to-treat' effect) |  |  |
| **Domain** | **Signalling question** | | | **Response** | **Comments** |
| **Bias arising from the randomization process** | 1.1 Was the allocation sequence random? | | | Y |  |
|  | 1.2 Was the allocation sequence concealed until participants were enrolled and assigned to interventions? | | | NI |  |
|  | 1.3 Did baseline differences between intervention groups suggest a problem with the randomization process? | | | N | There are no significant imbalances |
|  | **Risk of bias judgement** | | | **Some concerns** |  |
| **Bias due to deviations from intended interventions** | 2.1.Were participants aware of their assigned intervention during the trial? | | | NI |  |
|  | 2.2.Were carers and people delivering the interventions aware of participants' assigned intervention during the trial? | | | NI |  |
|  | 2.3. If Y/PY/NI to 2.1 or 2.2: Were there deviations from the intended intervention that arose because of the experimental context? | | | PN | No apparent deviations were reported. |
|  | 2.4 If Y/PY to 2.3: Were these deviations likely to have affected the outcome? | | | NA |  |
|  | 2.5. If Y/PY/NI to 2.4: Were these deviations from intended intervention balanced between groups? | | | NA |  |
|  | 2.6 Was an appropriate analysis used to estimate the effect of assignment to intervention? | | | PY |  |
|  | 2.7 If N/PN/NI to 2.6: Was there potential for a substantial impact (on the result) of the failure to analyse participants in the group to which they were randomized? | | | PN |  |
|  | **Risk of bias judgement** | | | **Low** |  |
| **Bias due to missing outcome data** | 3.1 Were data for this outcome available for all, or nearly all, participants randomized? | | | PY | The data for this outcome was available for nearly all participants who were randomized. |
|  | 3.2 If N/PN/NI to 3.1: Is there evidence that result was not biased by missing outcome data? | | | NA |  |
|  | 3.3 If N/PN to 3.2: Could missingness in the outcome depend on its true value? | | | NA |  |
|  | 3.4 If Y/PY/NI to 3.3: Is it likely that missingness in the outcome depended on its true value? | | | NA |  |
|  | **Risk of bias judgement** | | | **Low** |  |
| **Bias in measurement of the outcome** | 4.1 Was the method of measuring the outcome inappropriate? | | | PN | The methods used for measuring the outcome were appropriate. |
|  | 4.2 Could measurement or ascertainment of the outcome have differed between intervention groups? | | | PN | No difference in measurement was detected between the groups. |
|  | 4.3 Were outcome assessors aware of the intervention received by study participants? | | | NI |  |
|  | 4.4 If Y/PY/NI to 4.3: Could assessment of the outcome have been influenced by knowledge of intervention received? | | | PN |  |
|  | 4.5 If Y/PY/NI to 4.4: Is it likely that assessment of the outcome was influenced by knowledge of intervention received? | | | NA |  |
|  | **Risk of bias judgement** | | | **Low** |  |
| **Bias in selection of the reported result** | 5.1 Were the data that produced this result analysed in accordance with a pre-specified analysis plan that was finalized before unblinded outcome data were available for analysis? | | | PY |  |
|  | 5.2 ... multiple eligible outcome measurements (e.g. scales, definitions, time points) within the outcome domain? | | | PN | No multiple outcomes measurements. |
|  | 5.3 ... multiple eligible analyses of the data? | | | PN | No multiple analyses. |
|  | **Risk of bias judgement** | | | **Low** |  |
| **Overall bias** | **Risk of bias judgement** | | | **Some concerns** |  |
|  |  |  |  |  |  |
|  |  |  |  |  |  |
| **Unique ID** | Zhao et al. 2011 | **Study ID** | Zhao et al. 2011 | **Assessor** | Abdelrahman Hassan & Basant Katamesh |
| **Ref or Label** | Zhao et al. 2011 | **Aim** | assignment to intervention (the 'intention-to-treat' effect) |  |  |
| **Domain** | **Signalling question** | | | **Response** | **Comments** |
| **Bias arising from the randomization process** | 1.1 Was the allocation sequence random? | | | Y |  |
|  | 1.2 Was the allocation sequence concealed until participants were enrolled and assigned to interventions? | | | NI |  |
|  | 1.3 Did baseline differences between intervention groups suggest a problem with the randomization process? | | | N | There are no significant imbalances |
|  | **Risk of bias judgement** | | | **Some concerns** |  |
| **Bias due to deviations from intended interventions** | 2.1.Were participants aware of their assigned intervention during the trial? | | | NI |  |
|  | 2.2.Were carers and people delivering the interventions aware of participants' assigned intervention during the trial? | | | NI |  |
|  | 2.3. If Y/PY/NI to 2.1 or 2.2: Were there deviations from the intended intervention that arose because of the experimental context? | | | PN | No apparent deviations were reported. |
|  | 2.4 If Y/PY to 2.3: Were these deviations likely to have affected the outcome? | | | NA |  |
|  | 2.5. If Y/PY/NI to 2.4: Were these deviations from intended intervention balanced between groups? | | | NA |  |
|  | 2.6 Was an appropriate analysis used to estimate the effect of assignment to intervention? | | | PY |  |
|  | 2.7 If N/PN/NI to 2.6: Was there potential for a substantial impact (on the result) of the failure to analyse participants in the group to which they were randomized? | | | PN |  |
|  | **Risk of bias judgement** | | | **Low** |  |
| **Bias due to missing outcome data** | 3.1 Were data for this outcome available for all, or nearly all, participants randomized? | | | PY | The data for this outcome was available for nearly all participants who were randomized. |
|  | 3.2 If N/PN/NI to 3.1: Is there evidence that result was not biased by missing outcome data? | | | NA |  |
|  | 3.3 If N/PN to 3.2: Could missingness in the outcome depend on its true value? | | | NA |  |
|  | 3.4 If Y/PY/NI to 3.3: Is it likely that missingness in the outcome depended on its true value? | | | NA |  |
|  | **Risk of bias judgement** | | | **Low** |  |
| **Bias in measurement of the outcome** | 4.1 Was the method of measuring the outcome inappropriate? | | | PN | The methods used for measuring the outcome were appropriate. |
|  | 4.2 Could measurement or ascertainment of the outcome have differed between intervention groups? | | | PN | No difference in measurement was detected between the groups. |
|  | 4.3 Were outcome assessors aware of the intervention received by study participants? | | | NI |  |
|  | 4.4 If Y/PY/NI to 4.3: Could assessment of the outcome have been influenced by knowledge of intervention received? | | | PN |  |
|  | 4.5 If Y/PY/NI to 4.4: Is it likely that assessment of the outcome was influenced by knowledge of intervention received? | | | NA |  |
|  | **Risk of bias judgement** | | | **Low** |  |
| **Bias in selection of the reported result** | 5.1 Were the data that produced this result analysed in accordance with a pre-specified analysis plan that was finalized before unblinded outcome data were available for analysis? | | | PY |  |
|  | 5.2 ... multiple eligible outcome measurements (e.g. scales, definitions, time points) within the outcome domain? | | | PN | No multiple outcomes measurements. |
|  | 5.3 ... multiple eligible analyses of the data? | | | PN | No multiple analyses. |
|  | **Risk of bias judgement** | | | **Low** |  |
| **Overall bias** | **Risk of bias judgement** | | | **Some concerns** |  |
