# Supplementary material for: The impact of pulsatile vs. non-pulsatile perfusion in patients undergoing cardiopulmonary bypass: A comprehensive systematic review and meta-analysis of 33 randomized controlled trials
Source: PLoS One. 2025 Oct 14;20(10):e0333495. doi: 10.1371/journal.pone.0333495 (PMC12520390; doi:10.1371/journal.pone.0333495)
Supplement: S1 Table — (DOCX) [file pone.0333495.s005.docx]

**Table S1. Search strategy**

| Database | Search Terms | Search  Field | Search  Results |
| --- | --- | --- | --- |
| PubMed | (“pulsatile” OR “pulsatile flow*” OR “pump flow*” OR “pulsating flow*“ OR “pulsatile perfusion*” OR “oscillatory flow*” OR “Blood Flow Dynamics” OR “non pulsatile flow”) AND ((coronary artery bypass surgery) OR (CABG) OR “coronary surgery” OR “bypass graft*” OR “cardiopulmonary bypass*” OR “cardiac surgery” OR “valve replacement*” ) | All Fields | 194 |
| Central | (“pulsatile” OR “pulsatile flow*” OR “pump flow*” OR “pulsating flow*“OR “pulsatile perfusion*” OR “oscillatory flow*” OR “Blood Flow Dynamics” OR “non pulsatile flow”) AND ((coronary artery bypass surgery) OR (CABG) OR “coronary surgery” OR “bypass graft*” OR “cardiopulmonary bypass*” OR “cardiac surgery” OR “valve replacement*”) | All Fields | 301 |
| WOS | (“pulsatile” OR “pulsatile flow*” OR “pump flow*” OR “pulsating flow*“OR “pulsatile perfusion*” OR “oscillatory flow*” OR “Blood Flow Dynamics” OR “non pulsatile flow”) AND ((coronary artery bypass surgery) OR (CABG) OR “coronary surgery” OR “bypass graft*” OR “cardiopulmonary bypass*” OR “cardiac surgery” OR “valve replacement*”) | All Fields | 1429 |
| SCOPUS | (“pulsatile” OR “pulsatile flow*” OR “pump flow*” OR “pulsating flow*“OR “pulsatile perfusion*” OR “oscillatory flow*” OR “Blood Flow Dynamics” OR “non pulsatile flow”) AND ((coronary artery bypass surgery) OR (CABG) OR “coronary surgery” OR “bypass graft*” OR “cardiopulmonary bypass*” OR “cardiac surgery” OR “valve replacement*”) | Title, Abstract, Keywords | 1394 |
| Embase | (“pulsatile” OR “pulsatile flow*” OR “pump flow*” OR “pulsating flow*“OR “pulsatile perfusion*” OR “oscillatory flow*” OR “Blood Flow Dynamics” OR “non pulsatile flow”) AND ((coronary artery bypass surgery) OR (CABG) OR “coronary surgery” OR “bypass graft*” OR “cardiopulmonary bypass*” OR “cardiac surgery” OR “valve replacement*”) | All Fields | 1441 |
