# Supplementary figures and images for: The impact of pulsatile vs. non-pulsatile perfusion in patients undergoing cardiopulmonary bypass: A comprehensive systematic review and meta-analysis of 33 randomized controlled trials
Source: PLoS One. 2025 Oct 14;20(10):e0333495. doi: 10.1371/journal.pone.0333495 (PMC12520390; doi:10.1371/journal.pone.0333495)

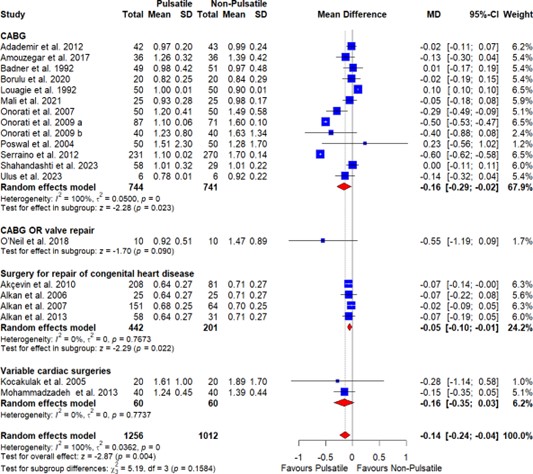

Supplement: S1 Fig — (JPG) [file pone.0333495.s006.jpg]

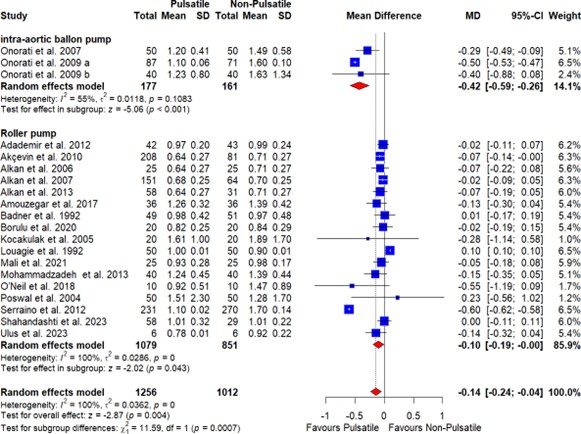

Supplement: S2 Fig — (JPG) [file pone.0333495.s007.jpg]

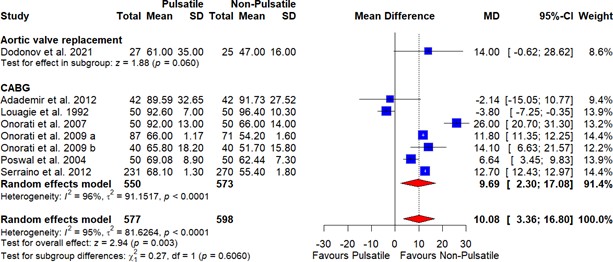

Supplement: S3 Fig — (JPG) [file pone.0333495.s008.jpg]

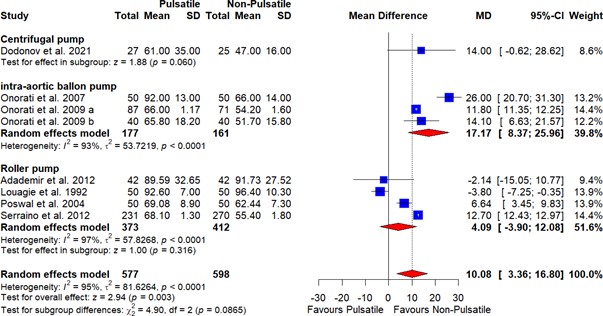

Supplement: S4 Fig — (JPG) [file pone.0333495.s009.jpg]

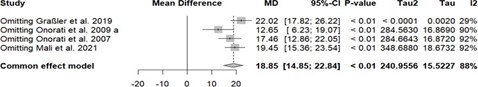

Supplement: S5 Fig — (JPG) [file pone.0333495.s010.jpg]

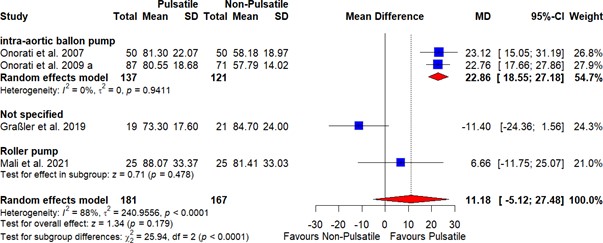

Supplement: S6 Fig — (JPG) [file pone.0333495.s011.jpg]

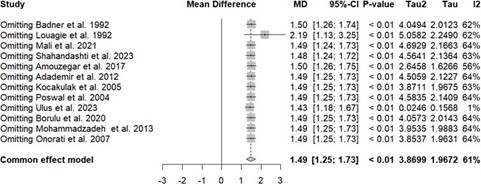

Supplement: S7 Fig — (JPG) [file pone.0333495.s012.jpg]

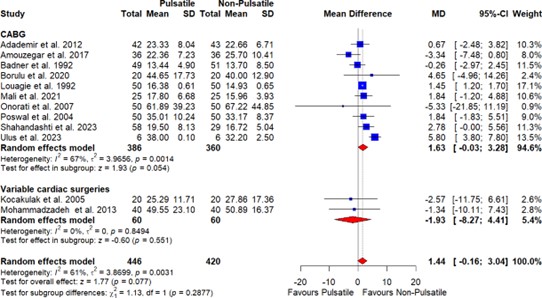

Supplement: S8 Fig — (JPG) [file pone.0333495.s013.jpg]

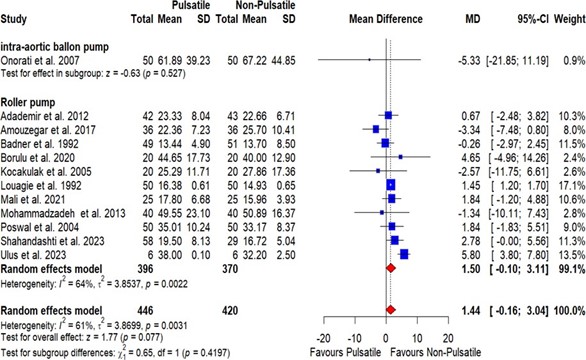

Supplement: S9 Fig — (JPG) [file pone.0333495.s014.jpg]

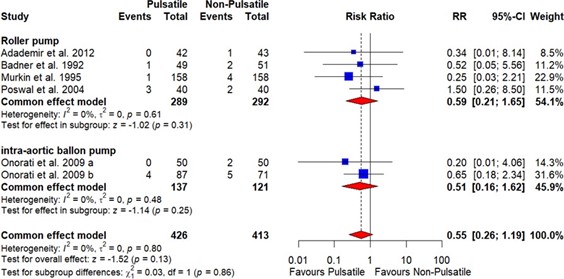

Supplement: S10 Fig — (JPG) [file pone.0333495.s015.jpg]

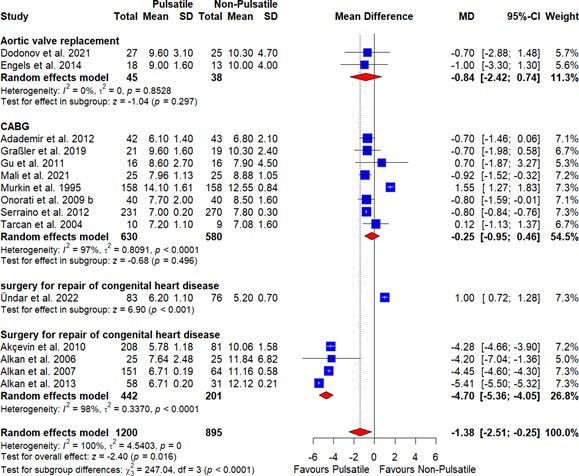

Supplement: S11 Fig — (JPG) [file pone.0333495.s016.jpg]

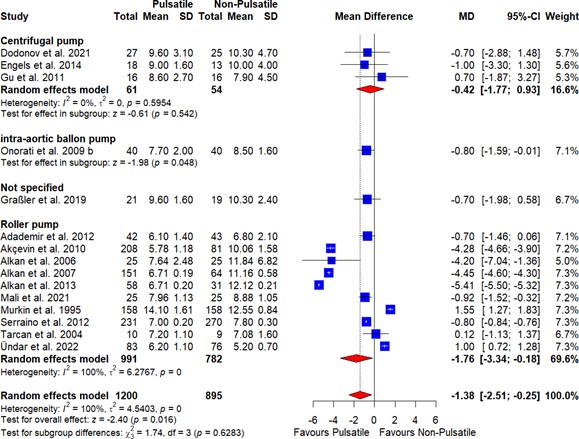

Supplement: S12 Fig — (JPG) [file pone.0333495.s017.jpg]

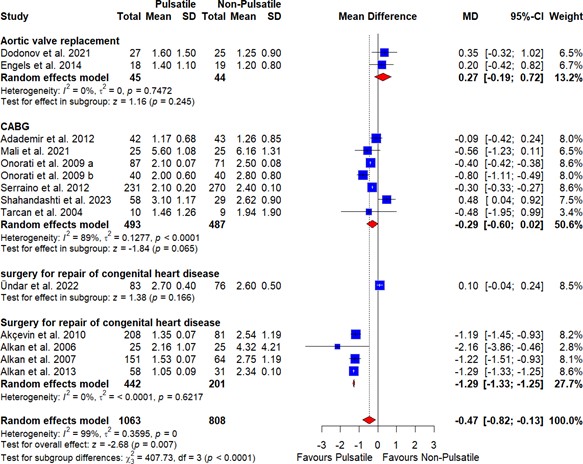

Supplement: S13 Fig — (JPG) [file pone.0333495.s018.jpg]

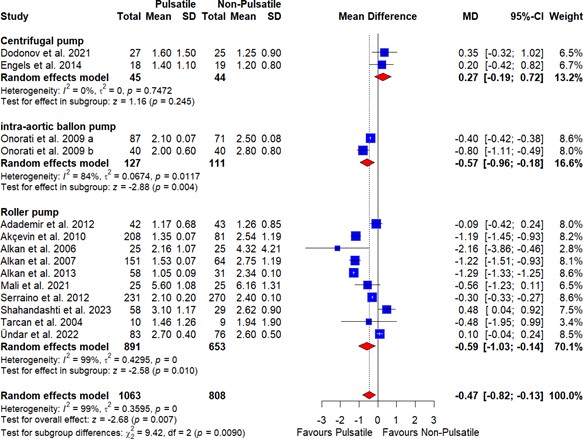

Supplement: S14 Fig — (JPG) [file pone.0333495.s019.jpg]

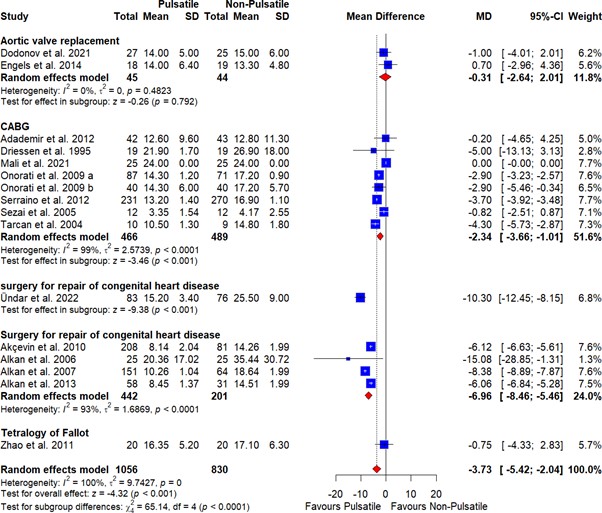

Supplement: S15 Fig — (JPG) [file pone.0333495.s020.jpg]

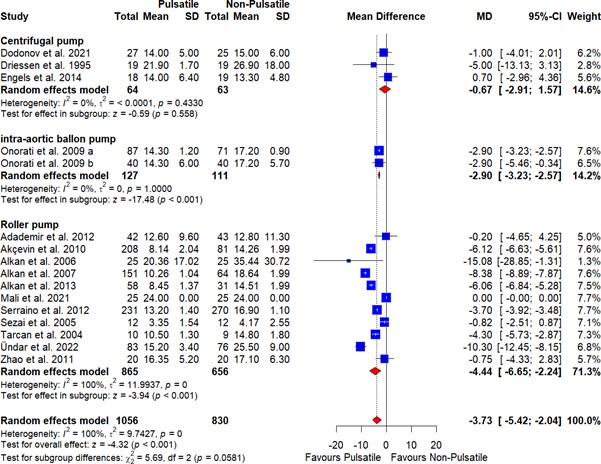

Supplement: S16 Fig — (JPG) [file pone.0333495.s021.jpg]

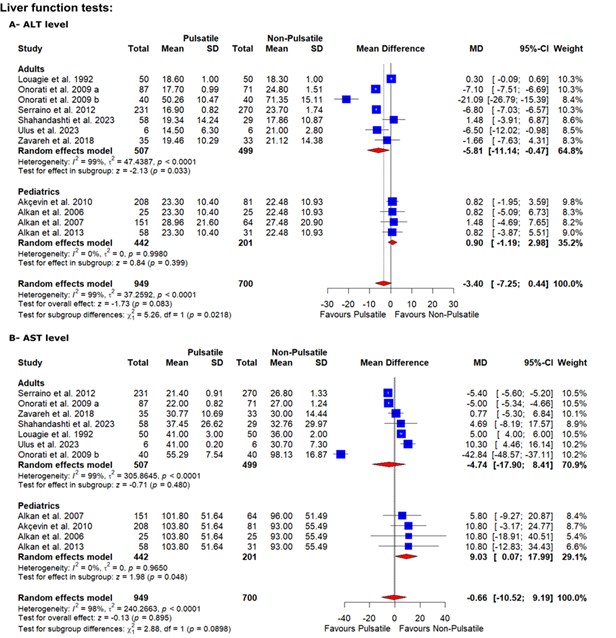

Supplement: S17 Fig — (JPG) [file pone.0333495.s022.jpg]

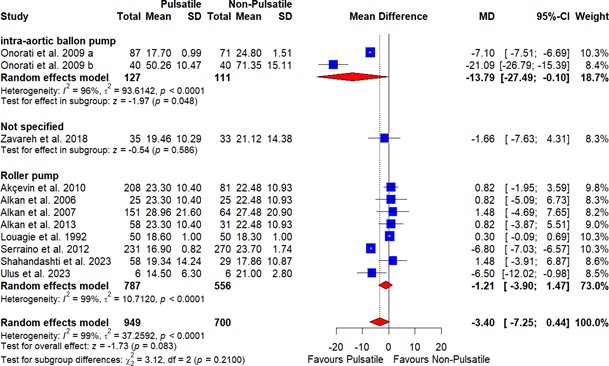

Supplement: S19 Fig — (JPG) [file pone.0333495.s024.jpg]

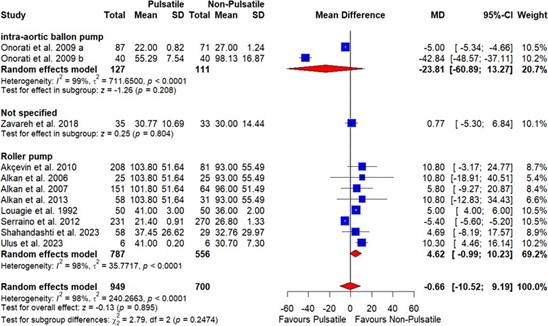

Supplement: S20 Fig — (JPG) [file pone.0333495.s025.jpg]

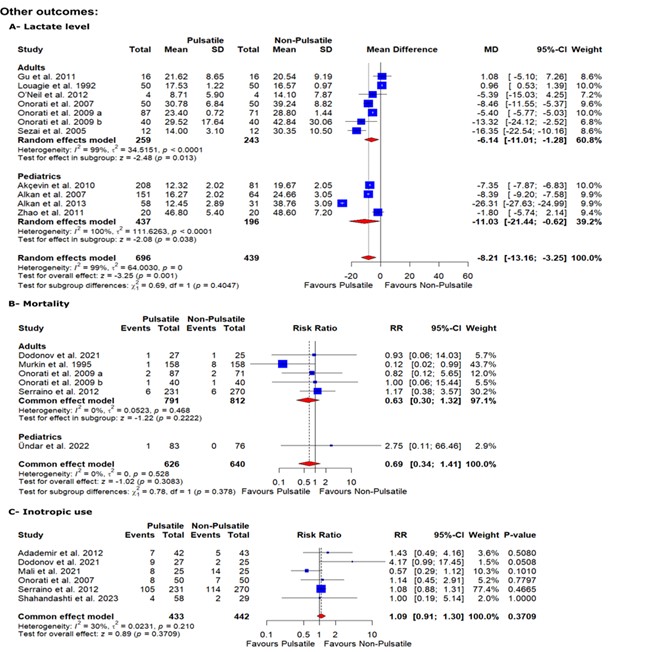

Supplement: S22 Fig — (JPG) [file pone.0333495.s027.jpg]

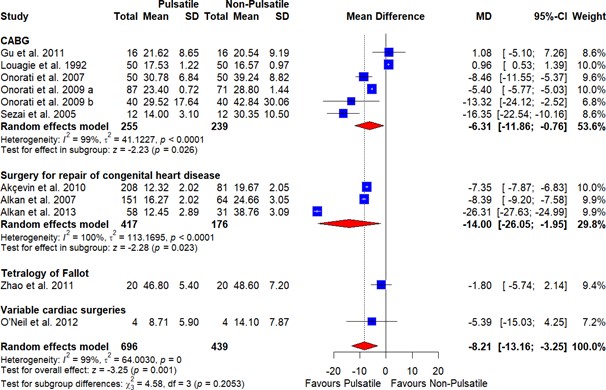

Supplement: S23 Fig — (JPG) [file pone.0333495.s028.jpg]

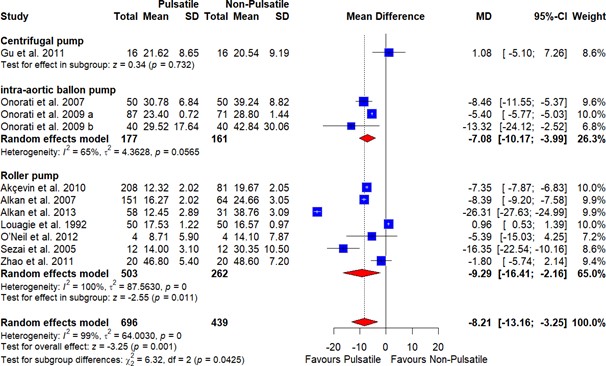

Supplement: S24 Fig — (JPG) [file pone.0333495.s029.jpg]

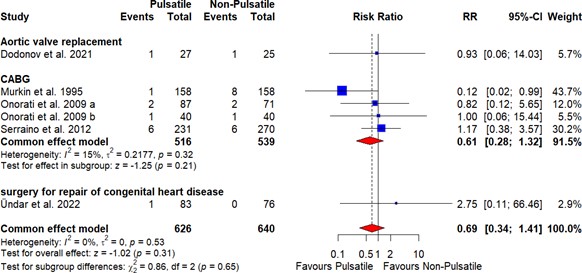

Supplement: S25 Fig — (JPG) [file pone.0333495.s030.jpg]

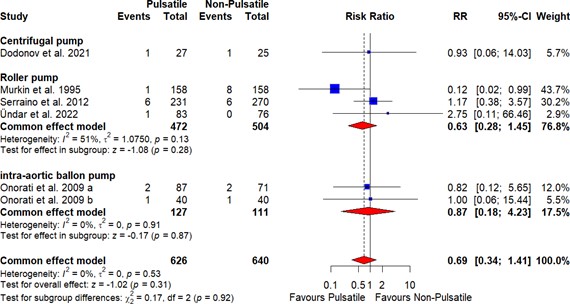

Supplement: S26 Fig — (JPG) [file pone.0333495.s031.jpg]

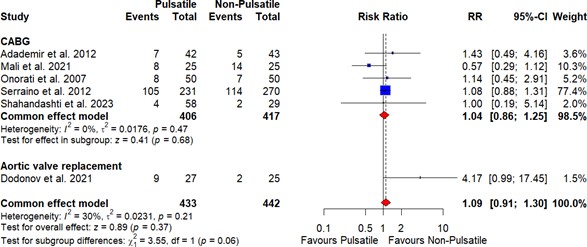

Supplement: S27 Fig — (JPG) [file pone.0333495.s032.jpg]

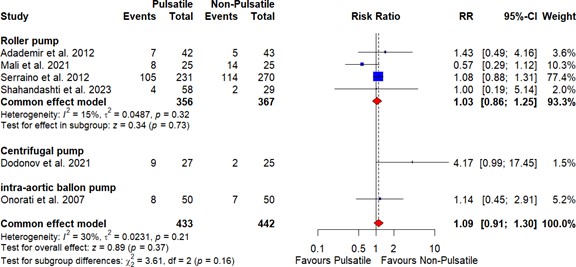

Supplement: S28 Fig — (JPG) [file pone.0333495.s033.jpg]

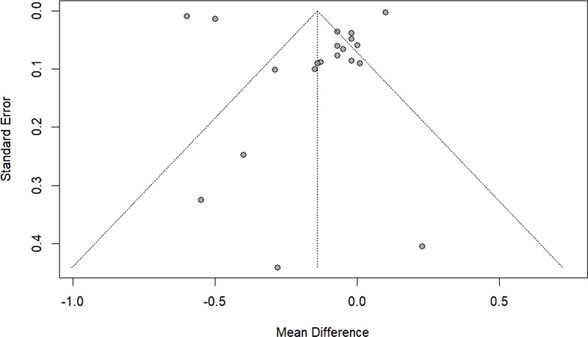

Supplement: S29 Fig — (JPG) [file pone.0333495.s034.jpg]

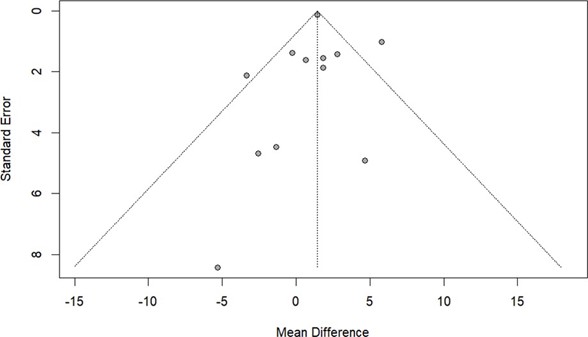

Supplement: S30 Fig — (JPG) [file pone.0333495.s035.jpg]

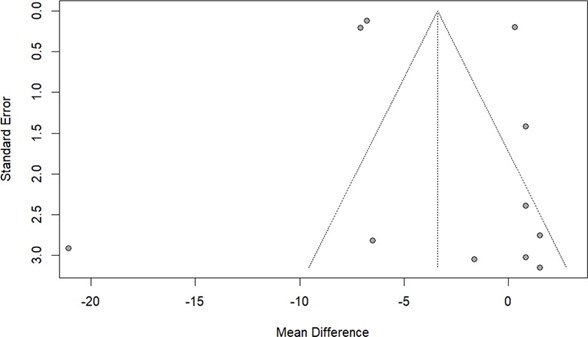

Supplement: S31 Fig — (JPG) [file pone.0333495.s036.jpg]

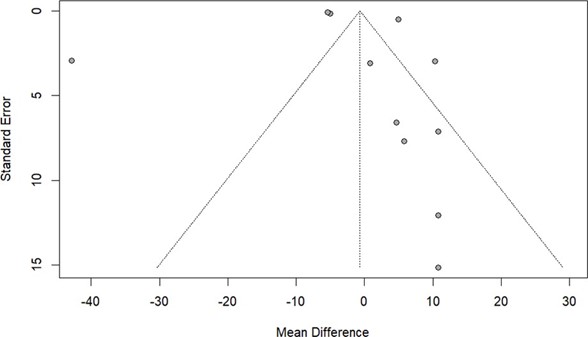

Supplement: S32 Fig — (JPG) [file pone.0333495.s037.jpg]

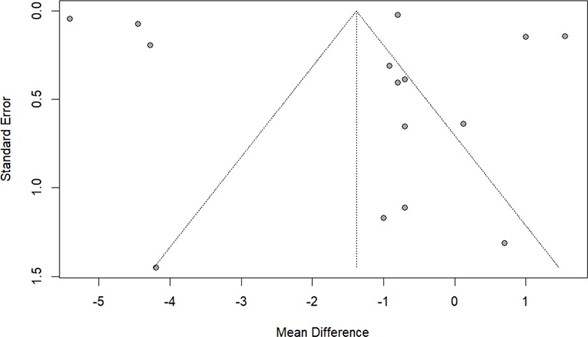

Supplement: S33 Fig — (JPG) [file pone.0333495.s038.jpg]

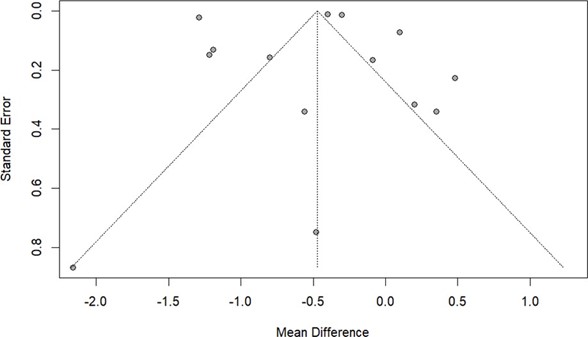

Supplement: S34 Fig — (JPG) [file pone.0333495.s039.jpg]

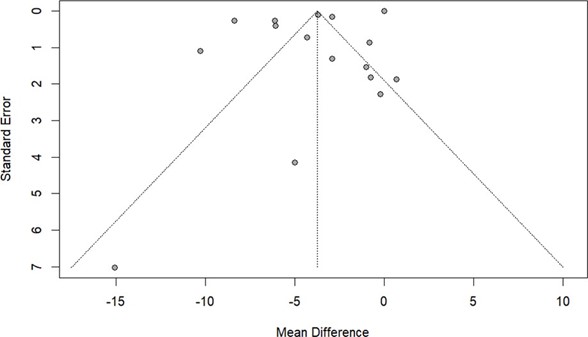

Supplement: S35 Fig — (JPG) [file pone.0333495.s040.jpg]

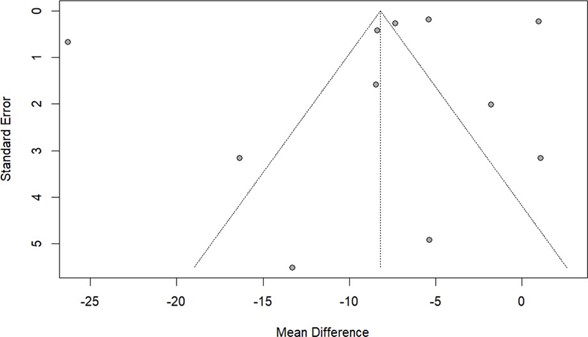

Supplement: S36 Fig — (JPG) [file pone.0333495.s041.jpg]
